# Peptidomimetic inhibitors of *N*-myristoyltransferase from human malaria and leishmaniasis parasites†
†Electronic supplementary information (ESI) available: Experimental procedures, characterization of intermediates and target compounds, description of biological assays, and crystallographic information are available in the Supplementary Information. The coordinates and structure factor files have been deposited in the Protein Data Bank with the accession codes: **4c68** (PvNMT-NHM-**10**), **4c7h** (LmNMT-MyrCoA-**10**) and **4c7i** (LmNMT-MyrCoA-**46**). See DOI: 10.1039/c4ob01669f



**DOI:** 10.1039/c4ob01669f

**Published:** 2014-09-18

**Authors:** Tayo O. Olaleye, James A. Brannigan, Shirley M. Roberts, Robin J. Leatherbarrow, Anthony J. Wilkinson, Edward W. Tate

**Affiliations:** a Department of Chemistry , Imperial College London , London , SW7 2AZ , UK . Email: e.tate@imperial.ac.uk ; Tel: +44 (0)20 7594 3752; b Structural Biology Laboratory , Department of Chemistry , University of York , York , YO10 5DD , UK

## Abstract

*N*-Myristoyltransferase (NMT) has been shown to be essential in *Leishmania* and subsequently validated as a drug target in *Plasmodium*. Herein, we discuss the use of antifungal NMT inhibitors as a basis for inhibitor development resulting in the first sub-micromolar peptidomimetic inhibitors of *Plasmodium* and *Leishmania* NMTs. High-resolution structures of these inhibitors with *Plasmodium* and *Leishmania* NMTs permit a comparative analysis of binding modes, and provide the first crystal structure evidence for a ternary NMT-Coenzyme A/myristoylated peptide product complex.

## Abstract 2

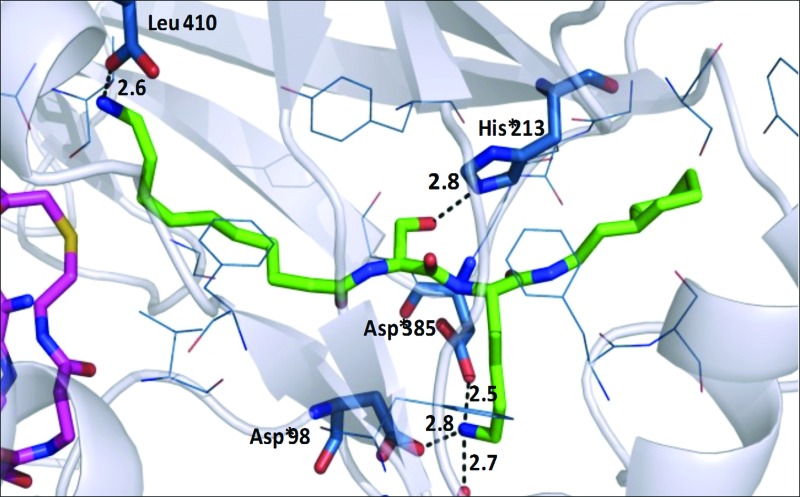
Peptidomimetic inhibitors of *N*-myristoyltransferase from malaria and leishmaniasis parasites have been designed with nanomolar potency, and reveal the first direct structural evidence for a ternary NMT/CoA/myristoyl peptide product complex.

Malaria and leishmaniases, infectious diseases caused by *Plasmodium* and *Leishmania* respectively, rank among the world's most important public health challenges. These diseases result in high mortality and morbidity; moreover, they impose a severe economic burden on affected countries, mainly in Africa. Of the *Plasmodium* genus, *P. falciparum* is the most deadly, accounting for over 1 million deaths in 2010, and *P. vivax* is the most widespread.^1^ Although malaria-related deaths have reduced by 30% in recent years, resistance to current anti-malarial drugs presents an ongoing challenge, highlighting the need to identify and develop safer and preferably less resistance-prone treatments.^2^ Visceral leishmaniasis, the most deadly of the leishmaniases, is caused by *L. donovani*, and accounts for ∼50 000 deaths per annum.^3^ Currently available treatments can be expensive, possess a relatively narrow therapeutic window or are subject to resistance, highlighting the need for better drugs.^4^

*N*-Myristoyltransferase (NMT), an enzyme that modifies protein substrates by attaching myristate (14 : 0) to an N-terminal glycine *via* an amide bond, has been proposed as a potential therapeutic target in both malaria and leishmaniasis^5,6^ and has recently been validated as viable drug target for human malaria.^7^ Catalysis is thought to commence with ordered binding of *S*-myristoyl-coenzyme A (Myr-CoA) followed by the protein substrate, transfer of myristate and ordered release of myristoylated protein and free coenzyme A.^8^ Myristoylation is important for protein–protein and protein–membrane interactions; the essentiality of NMT in the viability of both fungal and protozoan organisms^9,10^ makes NMT an interesting target for the development of antifungal and anti-parasitic drugs.^11,12^

Peptidomimetic inhibitors based on peptide substrates of NMT have previously been developed against *Candida albicans* NMT (CaNMT),^13,14^ but have yet to be reported in the context of parasitic NMT inhibition. CaNMT shares 44% and 43% sequence identity with *P. vivax* and *L. donovani* NMTs (PvNMT, LdNMT) respectively; we reasoned that inhibitors of *Plasmodium* and *Leishmania* NMTs might be acquired through a ‘piggy-back’ approach, using CaNMT peptidomimetics as a platform.^15^ Reported CaNMT peptidomimetic inhibitors were based on residues 1–7 at the N-terminus of *C. albicans* ADP ribosylation factor protein, GLYASKL. Subsequently, the N-terminal amine and Ser5-Lys6 dipeptide, a motif also seen in known substrates of *Plasmodium* and *Leishmania* NMTs, were identified as making important binding contributions.^5,7^ We therefore chose to employ a similar peptidomimetic scaffold based on the Ser-Lys motif, substituting the first four amino acids with an alkyl chain capped by a group that mimics the N-terminal amine, and the C-terminal leucine with a hydrophobic motif (Fig. 1). Our inhibitor library design incorporated modifications at the C- and N-termini with the objective of exploring contacts at both ends of the scaffold. Peptidomimetics were synthesized through a combination of solid and solution phase chemistries. *N*-Boc-protected amino and 1*H*-imidazol-1-yl acids were coupled to an *O-t*-butyl serine/*N*-Boc lysine dipeptide linked *via* a chlorotrityl (Route A, Scheme 1) or hydrazinobenzoyl linker (Route B, Scheme 1) to polystyrene resin. In the case of chlorotrityl resins, intermediates were cleaved from the resin with 0.5% TFA–DCM and coupled to the requisite amine (Scheme 1). C-terminal amide and acid analogs were synthesized using similar chemistry on Rink amide and Wang resins, respectively.

**Fig. 1:**
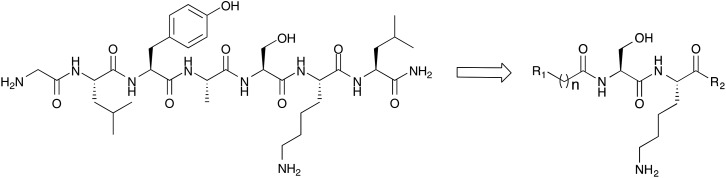
Peptidomimetic scaffold targeting parasite NMTs. R_1_ and R_2_ represent points of variation at the N- and C-termini.

**Scheme 1:**
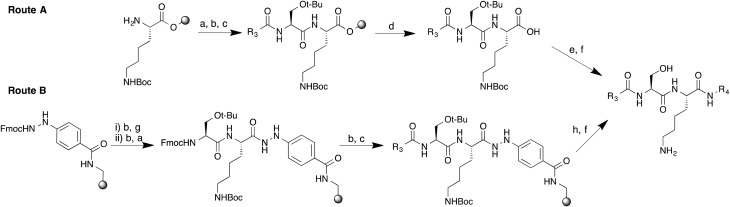
Synthetic routes to peptidomimetics. Reagents and conditions. (a) Fmoc-Ser(*t*-Bu)-OH, HBTU, DIPEA, DMF; (b) 20% piperidine–DMF; (c) R_3_COOH, HATU, DIPEA, DMF; (d) 0.5% TFA in DCM; (e) R_4_NH_2_, HATU, DIPEA, DMF; (f) TFA–TIPS–H_2_O (95 : 2.5 : 2.5); (g) Fmoc-Lys(Boc)-OH, HBTU, DIPEA, DMF; (h) Cu(OAc)_2_, pyridine, DCM, R_4_NH_2_.

Switching to a hydrazinobenzoyl linker eliminated the need for solution phase chemistry to couple the C-terminal amine;^16^ thus one-pot treatment of the peptidyl-resin in the presence of copper(ii) acetate and excess of the appropriate amine led to simultaneous cleavage of the peptide from the resin and formation of the corresponding protected peptidomimetic.

Final products were deprotected by treatment with TFA and purified by semi-preparative RP-HPLC-MS to give a small library of peptidomimetic inhibitors. Modifications at the N-terminus were approached by two distinct structural permutations: varying the N-terminal motif (R_1_, Fig. 1) to optimize basicity, and varying the alkyl linker length (*n*) to determine the optimal distance between the N-terminal amide of the serine and the N-terminal nitrogen atom of R_1_ (Fig. 1). The N-terminal motif of the peptidomimetic was expected to interact directly with the C-terminal carboxylate leucine of the enzyme, a critical residue for catalytic activity responsible for the activation of the N-terminal amine of the protein/peptide substrate.^17^

2-Methylimidazolyl analogues were synthesized building on the hypothesis that 2-methylimidazole (p*K*_a_ ∼ 7.9)^18^ is a reasonable mimetic for N-terminal Gly in the peptide substrate (p*K*_a_ ∼ 8.0).^19^ To cover a range of basicity, imidazolyl (p*K*_a_ ∼ 7.0) and amine analogues (p*K*_a_ ∼ 10.0) were also synthesized, each exploring a range of chain lengths (*n*) (Table 1). To probe versatility at the C-terminus, primary amides, carboxylic acids, 2-cyclohexylethyl and 2-(1-cyclohexenyl)ethyl amides were incorporated. Using a previously reported fluorogenic assay,^20^ inhibitors were tested against the parasitic enzymes and HsNMT1 in order to explore their selectivity against a relevant host enzyme. Previously reported imidazolyl-derived peptidomimetics (**1–4**) shown to possess low-μM activities against CaNMT^14^ displayed little or no activity against the parasitic enzymes used in this study. Slightly improved potency was observed for 2-methylimidazolyl analogues (**5–8**) across all chain lengths tested, and particularly **6**, which provided sub-micromolar inhibition for *L. donovani* NMT. However, amine **9** showed markedly improved inhibition against the NMTs of *P. vivax* (PvNMT), *L. donovani* (LdNMT) and *H. sapiens* (HsNMT1) (Table 1). Reduction of the alkyl chain length from *n* = 10 to 9 gave compound **10**, which is the most potent *L. donovani* NMT inhibitor reported to date (LdNMT IC_50_ = 24 nM). It also showed somewhat lower activity against HsNMT1 (IC_50_ = 60 nM) and PvNMT (680 nM). Further reduction of the chain length (**11** and **12**, *n* = 8 and 7, respectively) led to loss of detectable activity against *Plasmodium* NMTs and significant loss of activity against LdNMT and HsNMT1. Comparing N-terminal variations with similar chain length, the potency of amine **10** against LdNMT was over 400- and 20-fold higher than **2** (1*H*-imidazol-1-yl) and **6** (2-methyl-1*H*-imidazol-1-yl), respectively.

**Table 1:** Structures and enzyme affinities for peptidomimetics synthesized in this study^*a*^

	R_1_	*n*	R_2_	IC_50_ (μM)			
				PvNMT	PfNMT	LdNMT	HsNMT1
**1**	1*H*-Imidazol-1-yl	10	2-Cyclohexylethanamine	>100	>100	25.8 ± 8.2	47.6 ± 3.8
**2**	1*H*-Imidazol-1-yl	9	2-Cyclohexylethanamine	>100	>100	10.6 ± 1.6	44.0 ± 7.1
**3**	1*H*-Imidazol-1-yl	8	2-Cyclohexylethanamine	>100	>100	16.7 ± 2.5	>100
**4**	1*H*-Imidazol-1-yl	7	2-Cyclohexylethanamine	>100	>100	34.8 ± 3.7	>100
**5**	2-Methyl-1*H*-Imidazol-1-yl	10	2-Cyclohexylethanamine	>100	>100	>100	21.8 ± 0.8
**6**	2-Methyl-1*H*-Imidazol-1-yl	9	2-Cyclohexylethanamine	>100	>100	0.63 ± 0.01	7.92 ± 0.83
**7**	2-Methyl-1*H*-Imidazol-1-yl	8	2-Cyclohexylethanamine	>100	>100	3.42 ± 0.34	49.9 ± 10.6
**8**	2-Methyl-1*H*-Imidazol-1-yl	7	2-Cyclohexylethanamine	>100	>100	1.46 ± 0.22	82.2 ± 14.0
**9**	H_2_N–	10	2-Cyclohexylethanamine	1.04 ± 0.01	>100	0.14 ± 0.01	0.34 ± 0.03
**10**	H_2_N–	9	2-Cyclohexylethanamine	0.68 ± 0.08	24.3 ± 3.4	0.024 ± 0.003	0.06 ± 0.003
**11**	H_2_N–	8	2-Cyclohexylethanamine	>100	>100	2.01 ± 0.30	7.68 ± 0.86
**12**	H_2_N–	7	2-Cyclohexylethanamine	>100	>100	1.39 ± 0.18	6.75 ± 0.45
**13**	MeNH–	9	2-Cyclohexylethanamine	>100	>100	0.21 ± 0.01	0.73 ± 0.10
**14**	H_3_C–	0	2-Cyclohexylethanamine	>100	>100	>100	>100
**15**	H_2_N–	10	2-(1-Cyclohexenyl)-ethanamine	12.9 ± 1.07	>100	6.60 ± 1.28	2.30 ± 0.14
**16**	H_2_N–	9	2-(1-Cyclohexenyl)-ethanamine	3.55 ± 0.38	>100	0.44 ± 0.04	0.67 ± 0.04
**17**	1*H*-Imidazol-1-yl	10	2-(1-Cyclohexenyl)-ethanamine	>100	>100	>100	>100
**18**	1*H*-Imidazol-1-yl	9	2-(1-Cyclohexenyl)-ethanamine	>100	>100	>100	>100
**19**	2-Methyl-1*H*-Imidazol-1-yl	10	2-(1-Cyclohexenyl)-ethanamine	>100	>100	>100	>100
**20**	2-Methyl-1*H*-Imidazol-1-yl	9	2-(1-Cyclohexenyl)-ethanamine	>100	>100	28.0 ± 2.5	>100
**21**	H_2_N–	10	–NH_2_	>100	>100	13.3 ± 2.0	5.40 ± 0.41
**22**	H_2_N–	9	–NH_2_	22.9 ± 3.5	>100	1.36 ± 0.20	1.36 ± 0.29
**23**	H_2_N–	8	–NH_2_	>100	>100	27.1 ± 2.6	33.1 ± 2.5
**24**	H_2_N–	7	–NH_2_	>100	>100	61.9 ± 4.7	>100
**25**	H_2_N–	10	–OH	>100	>100	86.7 ± 10.4	39.2 ± 2.0
**26**	H_2_N–	9	–OH	>100	>100	>100	92.1 ± 17.6

^*a*^Enzymatic activities of recombinant *P. vivax*, *P. falciparum* and *L. donovani* NMT in the presence of peptidomimetic inhibitors expressed as IC_50_ values. These values are a mean of duplicate or triplicate experiments.

We next probed the SAR around the amino group of **10**, and found that N-methylation (to give **13**) led to significant reduction in potency, whilst replacing the flexible N-terminal chain with an acetyl group (to give **14**) resulted in no observable activity.

We further probed the importance of charge at the N-terminus by substituting a hydroxyl for the amine and observed a more modest loss in activity of >100 and 1000 folds in *Leishmania* and Human NMTs respectively (**46**, ESI,† accession code: 4c7i). These observations are consistent with our expectation that the N-terminal moiety of the inhibitor is involved in a strong electrostatic interaction with the C-terminal carboxylate of the enzyme, an interaction likely to be sensitive to changes in inhibitor structure and charge.^21^ Amongst inhibitors with a C-terminal 2-(1-cyclohexenyl)ethanamide (**15–20**, Table 1), **16** showed fair activity against LdNMT, HsNMT1 and PvNMT, whilst others showed little (**15**) or no activity (**17–20**) against the tested enzymes up to the highest concentration tested (100 μM). This 10–20 fold drop in activity relative to 2-cyclohexylethanamide suggests that the presence of a single unsaturated bond in the pocket occupied by the cyclohexenyl ring deters important interactions with the enzyme, presumably by modifying ring conformation. Inhibitors with C-terminal carboxamides and carboxylic acids (**21–26**) showed minimal activity across the enzymes tested, with the exception of **22** (Table 1) with an *n* = 9 chain length. Overall, an ideal chain length of *n* = 9 and a C-terminal cyclohexyl ring was observed to be the most potent combination irrespective of the enzyme tested, and at the N-terminus, inhibitor potencies increased in the order: 1*H*-imidazol-1-yl < 2-methyl-1*H*-imidazol-1-yl < –NH_2_.

To determine the binding mode of **10**, a ternary complex with PvNMT and *S*-(2-oxo)pentadecyl-CoA (NHM, a non-hydrolysable Myr-CoA analogue) was crystallized and solved to a high resolution of 1.38 Å (accession code: 4c68, Fig. 2A). **10** sits in the peptide-binding pocket, and mimics the key recognition elements involved in binding the parent peptide (GLYASKL, Fig. 2B and C). The N-terminal amine electrostatically interacts with the carboxylate of Leu410 whilst the hydroxyl group of the serine hydrogen bonds to the His213 side chain. In addition, there are ionic interactions between the amino group of the lysine and three neighboring aspartic acid residues (Asp98, Asp100, Asp385), all of which are conserved in equivalent residues CaNMT. The aliphatic chain on the N-terminus and the N-terminal amino group of the inhibitor are guided by the peptide binding channel to the C-terminal residue of PvNMT–Leu410. For comparison, a ternary structure of **10** with the native substrate, Myr-CoA, in *L. major* NMT (97% sequence identity to *L. donovani*) was solved and refined to a resolution of 1.41 Å (accession code: 4c7h). As expected, the mode of binding of **10** in *L. major* is very similar to that in *P. vivax*.

**Fig. 2:**
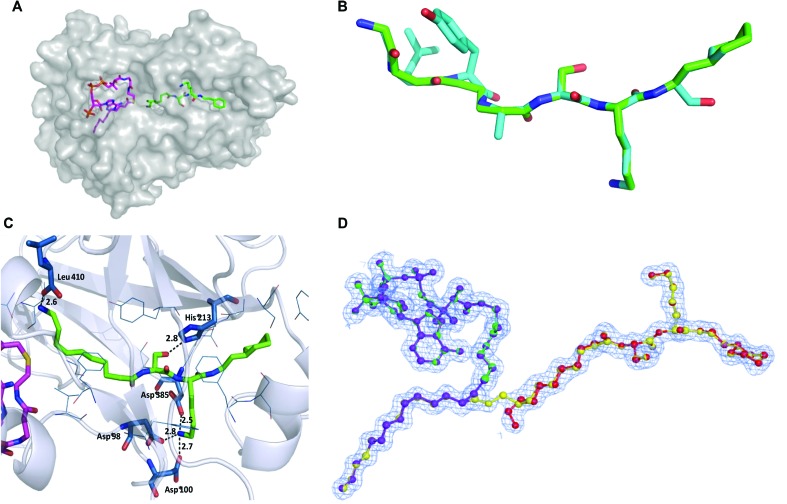
(A) Co-crystal structure of PvNMT (chain C; grey surface) with bound NHM (magenta) and **10** (green), colored by atom. (B) Superimposition of peptide substrate (GLYASKL, blue) with **10** (green, 4c68) in PvNMT. The peptide was modeled to maximize overall structural similarity while maintaining peptide geometric restraints. (C) Ternary structure of **10** (green, sticks) and non-hydrolysable Myr-CoA (partially shown; magenta; sticks) in PvNMT (4c68) showing main recognition interactions between **10** and the enzyme. Residues within 4 Å of **10** are shown in blue. Polar interactions and their distances (in Å) are shown as dashed lines. (D) Refined electron density map showing the mixture of structures of **10** and myr-**10** (yellow) in a ball and stick representation observed in LmNMT (4c7h). The figure shows the mixture of ligands present in the final refined model (80% reactants – purple and red and 20% products – green and yellow) shown in a ball-and-stick representation to aid identification. Electron density figures were made using the program CCP4 mg.^22^ See Fig. S2 (ESI†) for more details.

Unexpectedly, the electron maps indicated a mixture of ligand structures such that ∼20% of the electron density represents a *N*-myristoylated **10** product complex with the CoA by-product in LmNMT (Fig. 2D). This is the only structural evidence for a product complex in any *N*-myristoyltransferase, and captures and supports the proposed mechanism of action of NMT just prior to regeneration of the active enzyme. Kinetic experiments were performed to study **10** both as a potential substrate and as an NMT inhibitor. As anticipated from the structures above, **10** appeared to behave as a peptide-competitive inhibitor but no substrate characteristics were detected in the assay up to 40 μM (Fig. S3 and S4, ESI†). The N-myristoylation of **10** observed above therefore presumably occurs within the crystal where all reagents are present at high effective concentrations, and turnover is blocked by limited egress of products.

To probe the degree to which **10** acts as a true peptidomimetic, we investigated the potential to switch from inhibitor to a peptidomimetic substrate. A glycine (**27**) or alanine (**28**) residue was incorporated in the N-terminal extension; **27** was found to be a substrate for *Leishmania* NMT with *K*_M_ = 1.3 μM, which compares favorably with the affinity of a CaNMT octapeptide substrate (GLYASKLS-NH_2_, *K*_M_ = 0.6 μM)^13^ and the model peptide substrate used for inhibitor assays (GSNKSKPK-NH_2_ *H. sapiens* p60*src*(2–9), *K*_M_ = 22.6 μM), although with a considerably reduced *V*_max_ (Fig. S4, ESI†). **28**, on the other hand, showed no detectable substrate activity. In native NMT substrates, N-terminal Gly is preferred to the exclusion of all other residues, with relatively non-sterically demanding residues such as alanine. The substrate properties of **27** and **28** suggest a highly peptidomimetic binding conformation, and imply that steric factors play a key role in the progression of the catalytic cycle, dictating the approach of the N-terminal amine to the C-terminal leucine of the enzyme.

The potent inhibitors discovered in this study showed low specificity for the *Plasmodium* NMTs in comparison to *Leishmania* and human NMTs. A sequence alignment of residues within 6 Å of the catalytic sites of *Leishmania* and Human NMTs (see ESI†) showed a 74% similarity between both enzymes. In comparison, a similar alignment of Human and *Plasmodium* NMTs showed a 53% similarity between residues. The catalytic site of *Leishmania* NMT is, therefore, thought to show more comparability to the Human enzyme than the *Plasmodium* NMTs. Additionally, there are key residues, such as Arg231 (LdNMT) and Lys308 (Human NMT), capable of forming hydrogen bond interactions which would otherwise be impossible in the *Plasmodium* NMTs (Gly225). It is thought that the presence of such basic centres could increase the binding and subsequently, potencies of these inhibitors for *Leishmania* and human NMTs.

Unusually, the inhibitors in this study showed a higher specificity for *P. vivax* NMT in comparison to *P. falciparum* NMT. An alignment of the primary sequences of both enzymes (see ESI†) highlighted four differing residues within 6 Å of the peptide binding pocket: Ile102, Tyr212, Ser228, Tyr334 (PvNMT) and Val102, Phe212, Cys228 and Phe334 (PfNMT). Of these differences, the replacement of Y334 to F334 in PfNMT constitutes a structural change in the peptide pocket that could lead to a loss of potential hydrogen bond interactions within the catalytic site. The reason for the specificity for PvNMT is unclear; however, we propose that the loss of such vital hydrogen bonds in PfNMT could be responsible for this observation.

## Conclusions

Using antifungal NMT inhibitors as a platform, we developed inhibitors of *Plasmodium* and *Leishmania* NMTs, resulting in the identification of an inhibitor, **10**, with sub-micromolar potency against *P. vivax* and *L. donovani* NMTs, and the most potent *Leishmania donovani* NMT inhibitor reported to date. High-resolution ternary structures of compound **10** were achieved in both PvNMT and LmNMT, with the latter structure revealing the first structural evidence supporting the proposed catalytic cycle of NMT. Although **10** shows moderate selectivity against human NMT, the structures presented here may serve as a useful tool to enable a structure-guided design of more potent and selective parasitic inhibitors.

## Funding sources and acknowledgements

This work was supported by the Medical Research Council (MRC; grants G0900278 and U117532067), the Wellcome Trust (grant 087792), and the Biotechnology and Biological Sciences Research Council (David Phillips Research Fellowship to E.W.T., grant BB/D02014X/1). We are also grateful to Imperial College for an Imperial College President and Rector's award and the Student Opportunities Fund for funding. We are grateful to Prof. Deborah Smith (University of York), Dr J. Hutton (Imperial College) and colleagues for helpful discussions and Diamond Light Source (Harwell, UK) for provision of excellent synchrotron radiation facilities.
