# Supplementary material for: Peptidomimetic inhibitors of N-myristoyltransferase from human malaria and leishmaniasis parasites
Source: Org Biomol Chem. 2014 Sep 18;12(41):8132–7. doi: 10.1039/c4ob01669f (PMC4224572; doi:10.1039/c4ob01669f)
Supplement: Supplementary file 1 [file OB-041-C4OB01669F-s001.pdf]

This journal is © The Royal Society of Chemistry 2014

Supplementary Information

Peptidomimetic Inhibitors of N-myristoyltransferase from Human Malaria and Leishmaniasis Parasites

Tayo O. Olaleye,[a] James A. Brannigan,[b] Shirley M. Roberts,[b] Robin J. Leatherbarrow,[a] Anthony J. Wilkinson,[b] and Edward W. Tate[a*]

[a] Department of Chemistry, Imperial College London, London, SW7 2AZ, United Kingdom
[b] Structural Biology Laboratory, Department of Chemistry, University of York, York, YO10 5DD, United Kingdom

e.tate@imperial.ac.uk

Table of Contents.

1. Synthesis and analysis of peptidomimetics S1
1.1. Chemistry S1
1.2. ^{1}H NMR of peptidomimetics S17
1.3. Sequence alignments of NMTs from different species. S43
2. X-ray crystallography S43
2.1. Protein purification and crystallization S43
2.2. Data collection and refinement. S44
3. Kinetic studies of Compound **10**. S47
4. References S49

1. Synthesis and analysis of peptidomimetics.

1.1. Chemistry

Unless otherwise stated, all chemicals and reagents were purchased and used without further purification from Sigma-Aldrich, Alfa-Aesar, Merck Chemicals, VWR or Acros Organics. The peptidomimetics were purified using a RP-HPLC Waters 2767 system fitted with a photodiode array and an ESI mass spectrometer. Separation was achieved using an XBridge prep C_{18} (5 µm, 19 mm x 100 mm) column with an elution gradient of water and methanol both containing 0.1% formic acid at a flow rate of 20 mL/min.

All final compounds were greater than 95% pure, unless otherwise stated. Nuclear magnetic resonance ^{1}H and ^{13}C NMR, were recorded using a Bruker AV spectrometer at 400 or 500 MHz. Accurate mass data were obtained by John Barton and Lisa Haigh at the Department of Chemistry, Imperial College London.

Synthesis of compound 10.

Fmoc-Ser(*t*-Bu)-Lys(Boc)-hydrazinobenzoyl resin, 29. In a syringe fitted with a frit, Fmoc-hydrazinobenzoyl AM NovaGel resin (814.7 mg, Loading: 0.49 mmol/g) was treated with

20% piperidine/DMF (3×5 mL) for 10 min per treatment. The resin was subsequently washed with DMF (3×3 mL), DCM (3×3 mL) and DMF (3×3 mL). Fmoc-Lys(Boc)-OH was coupled to the deprotected resin using a pre-made coupling cocktail of DIPEA (10 eq.), HBTU (5 eq.) and Fmoc-Lys(Boc)-OH (5 eq.) in DMF (2 mL per 100 mg of resin). The resin was left shaking in this cocktail for 1-2 h at room temperature, drained and double coupled for the same amount of time. The resin was washed with DMF (5×2 mL), DCM (3×2 mL), MeOH (3×2 mL) and Et_2O (3×2 mL). This procedure was repeated for the coupling of Fmoc-Ser(*t*-Bu)-OH to the resin to provide the dipeptide on resin, Fmoc-Ser(*t*-Bu)-Lys(Boc)-hydrazinobenzoyl which was stored in the desiccator for use in subsequent reactions.

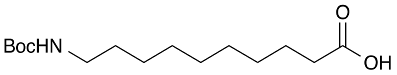

10-(*Tert*-butoxycarbonyl)aminodecanoic acid, 30.[1]
To commercially available 10-bromodecanoic acid (1 g, 3.98 mmol), NH_4OH (40 mL, 25% in H_2O) was added and stirred overnight. Upon completion, the mixture was evaporated to dryness. Water (2.1 mL), *tert*-butanol (2.1 mL, 22.3 mmol), NaOH (0.17 g, 4.18 mmol) and di-*tert*-butyl dicarbonate (0.91 g, 4.18 mmol) were added to the crude amine and stirred for 2 days at room temperature. The reaction was quenched by adding water (4.8 mL) and 1 M HCl (2 mL), extracted into ethyl acetate (3×10 mL), washed with brine, dried over Na_2SO_4 and concentrated to provide **30** as a white solid (1.02 g, 3.55 mmol, Yield = 89%). ^{1}H NMR (400 MHz, $CDCl_3$) δ 4.60 (1H, s), 3.09 (2H, t, J = 7.0 Hz), 2.34 (2H, t, J = 7.5 Hz), 1.63 (2H, p, J = 7.5 Hz), 1.48 – 1.45 (11H, m), 1.32 – 1.23 (10H, m). HRMS (ESI) *m/z* calcd for $C_{15}H_{29}NO_4$ $[M-H]^-$ 286.2018, found 286.2017.

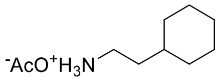

2-Cyclohexylethanaminium acetate, 31.[2] To commercially available 2-(1-cyclohexenyl)ethanamine (1.11 mL, 7.97 mmol) in methanol (83 mL), 10% palladium on carbon (0.50 g, 4.70 mmol) and acetic acid (0.83 mL, 14.5 mmol) were added. The suspension was stirred at room temperature and atmospheric pressure under an atmosphere of hydrogen for 3 days. The reaction mixture was filtered through Celite®, washed with methanol and the filtrate was concentrated to provide **31** as a golden yellow viscous oil in quantitative yield (1.81 g). ^{1}H NMR (400 MHz, $CDCl_3$) δ 4.92 (2H, s), 2.93 – 2.82 (2H, m), 1.95 (3H, s), 1.73 –1.59 (5H, m), 1.57 – 1.45 (2H, m), 1.36 – 1.04 (5H, m), 0.98 – 0.83 (2H, m). HRMS (CI) *m/z* calcd for $C_8H_{17}N$ $[M+H]^+$ 128.1395, found 128.1443.

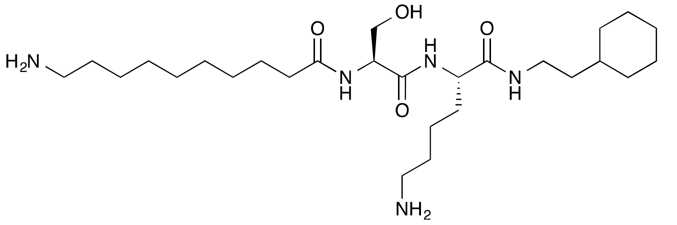

(10-Aminodecanoyl)-Ser-Lys-2-cyclohexylethylamide, 10. Fmoc-Ser(*t*-Bu)-Lys(Boc)-hydrazinobenzoyl resin (**29**, 100 mg) was swelled in DMF for at least 1 h and treated with 20% piperidine/DMF (3×3 mL) for 10 min per treatment. After the third treatment, the resin

was washed with DMF (3×3 mL), DCM (3×3mL) and DMF (3×3 mL). A coupling solution of DIPEA (85.3 µL, 0.49 mmol), HATU (93.2 mg, 0.245 mmol) and **30** (70.4 mg, 0.245 mmol) dissolved in DMF (2 mL) was added to the deprotected resin. Coupling was repeated twice for 1 h each time. The coupling mixture was drained and the resin was washed with DMF (5×2 mL), DCM (3×2 mL), MeOH (3×2 mL) and Et_2O (3×2 mL) and left to dry in a desiccator overnight. Oxidative cleavage occurred by suspending the resin in DCM (2 mL), $Cu(OAc)_2.H_2O$ (4.4 mg, 0.022 mmol), pyridine (53.9 µL, 0.67 mmol) and **31** (43.1 mg, 0.34 mmol). The reaction mixture was shaken in an open syringe at room temperature for 4 h. The resin was drained and washed with DCM (4×3 mL); the filtrate was washed with 1 M HCl and brine and concentrated under a flow of nitrogen. Deprotection using TFA/TIPS/H_2O for 1 h (2 mL, 95:2.5:2.5, *v/v*) led to the formation of **10** which was purified by preparative LCMS to provide a yellow oil (9 mg, Yield = 30%). ^{1}H NMR (500 MHz, MeOD) δ ppm 8.22 (1H, s), 4.37 – 4.27 (2H, m), 3.82 (1H, dd, *J* = 10.5, 5.6 Hz), 3.72 – 3.65 (1H, m), 3.17 (2H, t, *J* = 7.2 Hz), 2.93 – 2.83 (4H, m), 2.27 – 2.20 (2H, m), 1.98 – 1.85 (1H, m), 1.76 – 1.54 (11H, m), 1.52 – 1.09 (19H, m), 1.00 – 0.87 (2H, m). HRMS (ESI) *m/z* calcd for $C_{27}H_{53}N_5O_4$ $[M+H]^+$ 512.4131, found 512.4167. Rt: 10.73 min.

Synthesis of peptidomimetics 1-28, excluding 10.

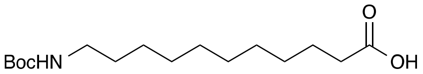

11-(*tert*-butoxycarbonylamino)undecanoic acid (32).[1]

Commercially available 11-aminoundecanoic acid (1.00 g, 4.98 mmol) was added to a round bottom flask containing water (2.4 mL), *tert*-butanol (2.4 mL), NaOH (0.21 g, 5.23 mmol, 1.05 eq) and Boc- anhydride (1.14 g, 5.22 mmol, 1.05 eq). The reaction mixture was stirred for 2 days at room temperature. The reaction was quenched by adding water (5.4 mL) and acidified using 1 M HCl (2.3 mL). The reaction mixture was extracted into ethyl acetate; the organic layer washed with brine, dried over Na_2SO_4 and concentrated to generate the desired product as a yellow oil (0.84 g, 2.77 mmol, Yield = 56%). ^{1}H NMR (400 MHz, $CDCl_3$) δ 4.54 (1H, br. s), 3.13 (2H, q, *J* = 6.2 Hz), 2.37 (2H, t, *J* = 7.4 Hz), 1.66 (2H, p, *J* = 7.4 Hz), 1.47 (11H, s), 1.39 (12H, s). HRMS (ESI) *m/z* calcd for $C_{16}H_{31}NO_4$ $[M-H]^-$ 300.2175, found 300.2170.

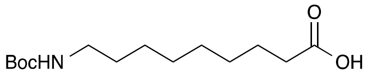

9-(*tert*-butoxycarbonylamino)nonanoic acid (33).[1]

Using a literature procedure,[3] 9-bromononanoic acid (0.46 g, Yield = 21.4%) was synthesised using Jones reagent (2M, 18 mL) in acetone (18 mL) from commercially sourced 9-bromononan-1-ol (2 g, 0.45 mol). *Tert*-butoxycarbonyl protection was carried out as described above to afford **33** which was isolated as an off-white solid (0.49g, Yield = 64%). ^{1}H NMR (400 MHz, $CDCl_3$) δ 4.52 (1H, s), 3.10 (2H, q, *J* = 6.8 Hz), 2.33 (2H, t, *J* = 7.5 Hz), 1.66 – 1.56 (2H, m), 1.51 – 1.41 (11H, m), 1.37 – 1.27 (8H, m). ^{13}C NMR (100 MHz, $CDCl_3$) δ 40.72, 34.11, 30.11, 29.16, 28.56, 28.36, 26.82, 24.83, 14.34. HRMS *m/z* calcd for $C_{14}H_{27}NO_4$ $[M-H]^-$ 272.1940 found 272.1861.

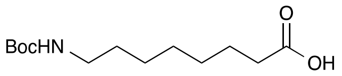

8-(*tert*-butoxycarbonylamino)octanoic acid (34).[1, 4]

The product was synthesized according to a previously reported literature procedure and was isolated as a colourless oil (1.17 g, Yield = 59.7%). ^{1}H NMR (400 MHz, $CDCl_3$) δ 4.54 (1H, s), 3.09 (2H, q, *J* = 6.1 Hz), 2.32 (2H, t, *J* = 7.4 Hz), 1.69 – 1.53 (2H, m), 1.49 – 1.44 (11H, m), 1.31 (6H, s). HRMS *m/z* calcd for $C_{13}H_{25}NO_4$ $[M-H]^-$ 258.1784, found 258.1716.

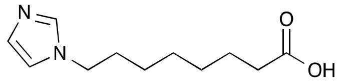

8-(1H-imidazol-1-yl)octanoic acid (35).[5]

Following a previously reported literature procedure,[6] *tert*-butyl 8-bromooctanoate, was synthesized from 8-bromooctanoic acid using *t*-BuOH (3 eq), DCC (1.15 eq), DMAP (0.1 eq) in DCM (6 mL) and stirred for 2 days at room temperature. The resulting ester was treated with sodium imidazolide (2 eq) in the presence of 18-crown-6 (2.1 eq) and DMF (12 mL) for 4.5 h at room temperature under nitrogen as described by Devadas *et al.*[7] Water (12 mL) was added and extracted into ethyl acetate (3×15 mL). The organic layer was washed with brine (2×15 mL), dried over $MgSO_4$ and concentrated under reduced pressure to give *tert*-butyl-8-(1*H*-imidazol-1-yl)octanoate in quantitative yields. Following treatment with TFA/DCM/TIS (8:11:1) for 1 h and cold TMBE, **35** was obtained as a white solid in quantitative yields. ^{1}H NMR (400 MHz, MeOD) δ 8.96 (1H, s), 7.70 – 7.63 (1H, m), 7.60 – 7.54 (1H, m), 4.25 (2H, t, *J* = 7.3 Hz), 2.29 (2H, t, *J* = 7.4 Hz), 1.91 (2H, p, *J* = 7.5 Hz), 1.60 (2H, p, *J* = 7.3 Hz), 1.46 – 1.27 (6H, m). HRMS *m/z* calcd for $C_{11}H_{18}N_2O_2$ $[M+H]^+$ 211.1402, found 211.1451.

This synthesis was applied in the synthesis of compounds **36-38** below.

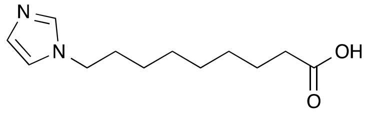

9-(1H-imidazol-1-yl)nonanoic acid (36).[4]

The product was obtained as a pale yellow oil (1.12 g, Yield = quant.) ^{1}H NMR (400 MHz, MeOD) δ 8.98 (1H, s), 7.70 – 7.65 (1H, m), 7.60 – 7.56 (1H, m), 4.26 (2H, t, *J* = 7.3 Hz), 2.29 (2H, t, *J* = 7.4 Hz), 1.91 (2H, p, *J* = 7.5 Hz), 1.65 – 1.50 (2H, m), 1.45 – 1.27 (8H, m). HRMS *m/z* calcd for $C_{12}H_{20}N_2O_2$ $[M+H]^+$ 225.1558 found 225.1609.

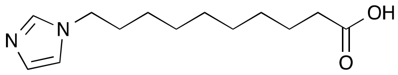

10-(1H-imidazol-1-yl)decanoic acid (37)

The product was obtained as a white solid (0.25 g, Yield = quant.). ^{1}H NMR (400 MHz, DMSO) δ 11.95 (1H, s), 9.06 (1H, s), 7.75 (1H, s), 7.64 (1H, s), 4.14 (2H, t, *J* = 7.2 Hz), 2.15 (2H, t, *J* = 7.3 Hz), 1.76 (2H, p, *J* = 7.3 Hz), 1.52 – 1.37 (2H, m), 1.21 (10H, s). HRMS *m/z* calcd for $C_{13}H_{22}N_2O_2$ $[M+H]^+$ 239.1715, found 239.1754.

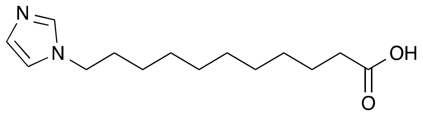

11-(1H-imidazol-1-yl)undecanoic acid (38).[7]

The product was obtained as a white solid (0.32 g, Yield = quant.). ^{1}H NMR (400 MHz DMSO) *δ* 9.11 (1H, s), 7.81 – 7.77 (1H, m), 7.70 – 7.68 (1H, m), 4.17 (2H, t, *J* = 7.3 Hz), 2.18 (2H, t, *J* = 7.3 Hz), 1.84 – 1.74 (2H, m), 1.53 – 1.37 (2H, m), 1.24 (12H, s). HRMS *m*/*z* calcd for $C_{14}H_{24}N_2O_2$ $[M-H]^-$ 251.1760, found 251.1762.

Compounds **39-42** were synthesised, in quantitative yields, in the same manner as **35-38** using 2-methylimidazole (2 eq), DMF, NaH (3 eq.) and 18-crown-6 (3.1 eq.). The compounds were used without further purification.

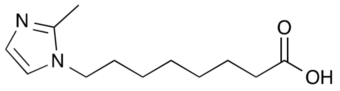

8-(2-methyl-1H-imidazol-1-yl)octanoic acid (39).

The product was obtained as a pale yellow oil (1.48 g, quant.). ^{1}H NMR (400 MHz, MeOD) δ 7.52 (1H, d, *J* = 2.1 Hz), 7.43 (1H, d, *J* = 2.2 Hz), 4.13 (2H, t), 2.64 (3H, s), 2.29 (2H, t, *J* = 7.3 Hz), 1.85 (2H, p, *J* = 7.7 Hz), 1.61 (2H, p, *J* = 7.3 Hz), 1.42 – 1.32 (6H, m). HRMS *m*/*z* calcd for $C_{12}H_{20}N_2O_2$ $[M+H]^+$ 225.1558, found 225.1606.

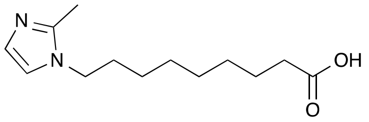

9-(2-methyl-1H-imidazol-1-yl)nonanoic acid (40).

The product was obtained as a pale yellow oil (0.77 g, Yield = quant.). ^{1}H NMR (400 MHz, MeOD) δ 7.52 (1H, d, *J* = 2.1 Hz), 7.42 (1H, d, *J* = 2.0 Hz), 4.17 – 4.06 (2H, m), 2.63 (3H, s), 2.28 (2H, t, *J* = 7.4 Hz), 1.84 (3H, p, *J* = 7.4 Hz), 1.64 – 1.53 (2H, m), 1.44 – 1.30 (8H, m). HRMS *m*/*z* calcd for $C_{13}H_{22}N_2O_2$ $[M+H]^+$ 239.1715, found 239.1757.

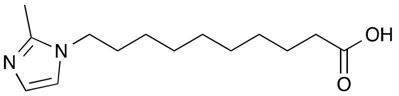

10-(2-methyl-1H-imidazol-1-yl)decanoic acid (41).

The product was obtained as an off-white solid (2.2 g, Yield = quant.). ^{1}H NMR (400 MHz, $CDCl_3$) δ 7.34 (1H, d, *J* = 2.1 Hz), 7.04 (1H, d, *J* = 2.1 Hz), 4.01 (2H, t, *J* = 7.4 Hz), 2.68 (3H, s), 2.38 (2H, t, *J* = 7.3 Hz), 1.84 (2H, p, *J* = 7.2 Hz), 1.63 (2H, p, *J* = 7.3 Hz), 1.41 – 1.27 (10H, m). HRMS *m*/*z* calcd for $C_{14}H_{24}N_2O_2$ $[M+H]^+$ 253.1871, found 253.1918.

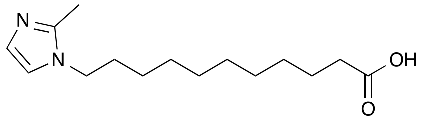

11-(2-methyl-1H-imidazol-1-yl)undecanoic acid (42).

The product was obtained as a pale yellow oil (1.4 g, Yield = quant.). ^{1}H NMR (400 MHz, $CDCl_3$) δ 7.33 (1H, s), 7.03 (1H, s), 4.00 (2H, t, *J* = 6.8 Hz), 2.72 (3H, s), 2.35 (2H, t,

J = 7.3 Hz), 1.92 – 1.72 (2H, m), 1.69 – 1.52 (2H, m), 1.43 – 1.16 (12H, m). HRMS *m/z* calcd for $C_{15}H_{26}N_2O_2$ $[M+H]^+$ 267.2028, found 267.2072.

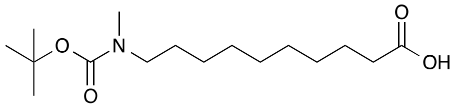

10-(*Tert*-butoxycarbonyl)methylaminodecanoic acid (43)
To 10-bromodecanoic acid (0.5 g, 1.99 mmol), methylamine (10 mL, 252.7 mmol) was added and left stirring at room temperature overnight. The reaction mixture was concentrated under reduced pressure to give an off-white solid. The crude product was *tert*-butoxycarbonyl protected as described above to afford a pale yellow oil. The crude product was purified by preparative LCMS to give a colourless oil (0.27 g, Yield = 45%). ^{1}H NMR (400 MHz, $CDCl_3$) δ 3.22 (2H, t, J = 6.8 Hz), 2.83 (3H, s), 2.28 (2H, t, J = 7.4 Hz), 1.59 (2H, m), 1.52 (2H, m), 1.45 (9H, s), 1.33 (10H, m). HRMS (ESI) *m/z* calcd for $C_{16}H_{31}NO_4$ $[M+Na]^+$ 324.2145, found 324.2138.

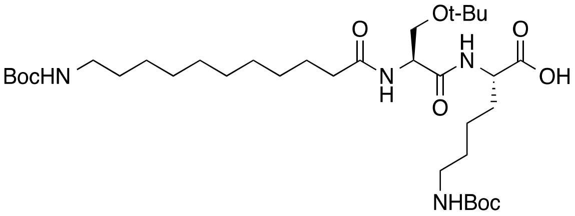

11-N-Boc-aminoundecanoic acid-Ser(*t*-Bu)-Lys(Boc)-H (44). The Fmoc protecting group was removed from Fmoc-Ser(*t*-Bu)-Lys(Boc)-2-ClTrt (Loading 0.61 mmol/g, 103 mg) following treatment with 20% piperidine/DMF (3 mL). A coupling solution of DIPEA (109 μL, 0.63 mmol), HBTU (119 mg, 0.31 mmol) and **32** (94.7 mg, 0.31 mmol) dissolved in DMF (2 mL) was added to the deprotected resin. Coupling was repeated twice for 1 h each time. The coupling mixture was drained and the resin was washed with DMF (5×2 mL), DCM (3×2 mL), MeOH (3×2 mL) and Et_2O (3×2 mL) and dried in a desiccator overnight. The peptide was cleaved off the chlorotrityl resin following treatment with 0.5% TFA/DCM (2×3 mL) for 30 min each time to provide 11-N-Boc-aminoundecanoic acid- Ser(*t*-Bu)-Lys(Boc)-OH which was purified using preparative LCMS to provide a yellow oil (19 mg, yield = 45%) and used in the subsequent reaction. HRMS (ESI) *m/z* calcd for $C_{34}H_{64}N_4O_9$ $[M+H]^+$ 673.4707, found 673.4749. Retention time (Rt): 8.21 min.

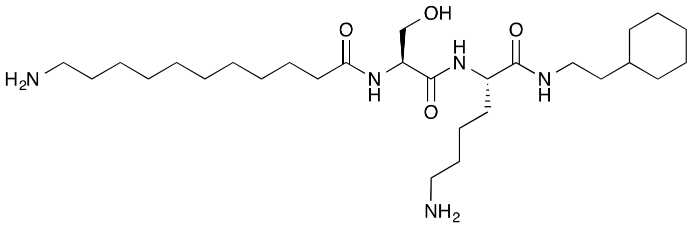

(11-Aminoundecanoyl)-Ser-Lys-2-cyclohexylethylamide (9).[7] To **44** (19 mg) in DMF (1 mL), DIPEA (20.7 μL), EDCI·HCl (7.04 mg, 0.037 mmol, 1.3 eq), HOAt (5.0 mg, 0.037 mmol, 1.3 eq) and **31** (10.6 mg, 0.057 mmol, 2 eq) were added. The suspension was stirred for 4 days at room temperature. Water (2 mL) was added, the reaction mixture was extracted into ethyl acetate (3×10 mL) and washed with brine. The organic layer was

concentrated and protecting groups were removed following treatment with TFA/TIPS/H_2O (1 mL, 95:2.5:2.5) for 1 h. Purification was achieved using preparative LCMS. The resulting product was **9** as a glassy translucent formic acid salt (11 mg, Yield = 28%). ^{1}H NMR (400 MHz, MeOD) δ ppm 4.39 – 4.30 (2H, m), 3.80 (1H, dd, *J* = 10.4, 5.6 Hz), 3.77 – 3.67 (1H, m), 3.19 (2H, t, *J* = 7.2 Hz), 2.98 – 2.85 (4H, m), 2.29 – 2.22 (2H, m), 2.03 – 1.88 (1H, m), 1.81 – 1.56 (13H, m), 1.56 – 1.44 (2H, m), 1.45 –1.12 (21H, m), 1.00 – 0.86 (2H, m). HRMS (ESI) *m/z* calcd for $C_{28}H_{55}N_5O_4$ $[M+H]^-$ 526.4288, found 526.3240.

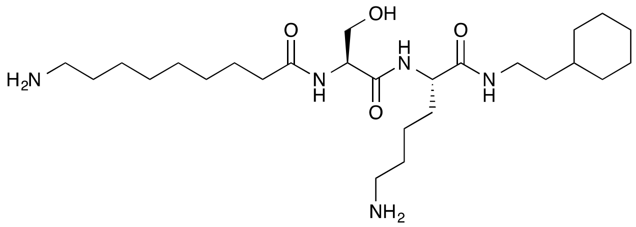

(9-Aminononanoyl)-Ser-Lys-2-cyclohexylethylamide (11).

This product was synthesised from **33** using the same method as compound **10** described above. The final product was a yellow oil (23 mg, Yield = 64%). ^{1}H NMR (500 MHz, MeOD) δ ppm 4.42 – 4.28 (2H, m), 3.87 (1H, dd, *J* = 10.5, 5.5 Hz), 3.78 – 3.72 (1H, m), 3.27 – 3.15 (2H, m), 3.00 – 2.90 (4H, m), 2.34 – 2.26 (2H, m), 1.99 – 1.92 (1H, m), 1.76 – 1.63 (11H, m), 1.59 – 1.47 (2H, m), 1.46 – 1.35 (10H, m), 1.33 –1.15 (5H, m), 1.01 – 0.89 (2H, m). HRMS (ESI) *m/z* calcd for $C_{26}H_{51}N_5O_4$ $[M+H]^+$ 498.3975, found 498.4029. Rt: 10.94 min.

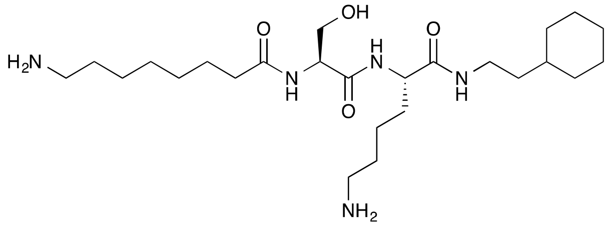

(8-Aminooctanoyl)-Ser-Lys-2-cyclohexylethylamide (12)

This product was synthesised from **34** using the same method as compound **10** described above. The final product was a yellow oil (6 mg, Yield = 17%). ^{1}H NMR (400 MHz, MeOD) δ ppm 8.51 (1H, s) 4.40 – 4.26 (2H, m), 3.81 (1H, dd, *J* = 10.4, 5.6 Hz), 3.73 – 3.64 (1H, m), 3.17 (2H, t, *J* = 7.3 Hz), 2.94 – 2.83 (4H, m), 2.25 (2H, t, *J* = 7.6 Hz), 1.98 – 1.85 (1H, m), 1.75 – 1.55 (12H, m), 1.54 – 1.40 (3H, m), 1.40 – 1.31 (8H, m), 1.31 – 1.08 (7H, m), 0.95 – 0.83 (2H, m). HRMS (ESI) *m/z* calcd for $C_{25}H_{49}N_5O_4$ $[M+H]^+$ 484.3818, found 484.3856. Rt: 9.64 min.

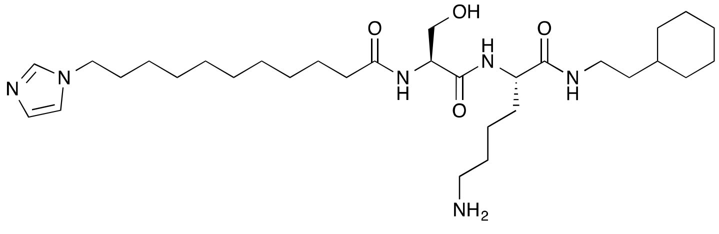

11-(1H-imidazol-1-yl)undecanoyl-Ser-Lys-2-cyclohexylethylamide (1)[7]

This product was synthesised from **38** using the same method as compound **9** but using HATU (93.2 mg, 3 eq), DIPEA (85.6 μL, 6 eq) and **31** over 2 days. The final product was a

pale pink oil (16.3 mg, Yield = 32%). ^{1}H NMR (400 MHz, MeOD) δ 8.75 (1H, s), 7.61 – 7.55 (1H, m), 7.51–7.45 (1H, m), 4.40–4.27 (2H, m), 4.21 (2H, t, *J* = 7.2 Hz), 3.83 (1H, dd, *J* = 10.6, 5.4 Hz), 3.82 – 3.67 (2H, m), 3.25 – 3.15 (2H, m), 2.97 – 2.84 (2H, m), 2.34 – 2.19 (2H, m), 1.99 – 1.82 (3H, m), 1.78 – 1.55 (11H, m), 1.55 – 1.42 (2H, m), 1.43 – 1.11 (20H, m), 1.00 – 0.83 (2H, m). HRMS (ESI*) m/z* calcd for $C_{31}H_{56}N_6O_4$ $[M+H]^+$ 577.4397, found 577.4404. Rt: 11.21 min.

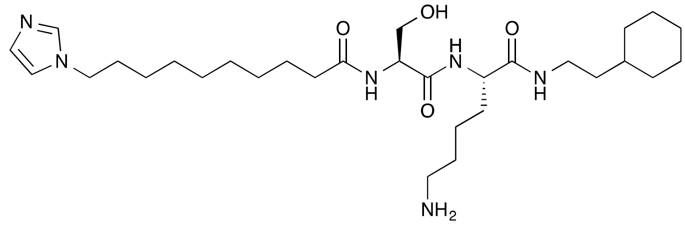

10-(1H-imidazol-1-yl)decanoyl-Ser-Lys-2-cyclohexylethylamide (2)

This product was synthesised from **37** using the same method as compound **10** described above. The final product was a yellow oil (2.9 mg, Yield = 10%). ^{1}H NMR (500 MHz, MeOD) δ 8.97 (1H, s), 7.70 – 7.64 (1H, m), 7.60 – 7.54 (1H, m), 4.39 – 4.33 (2H, m), 4.26 (2H, t, *J* = 7.3 Hz), 3.86 (1H, dd, *J* = 10.6, 5.6 Hz), 3.78 – 3.70 (1H, m), 3.20 (2H, t, *J* = 7.3 Hz), 2.94 (2H, t, *J* = 7.0 Hz), 2.27 (2H, t, *J* = 7.6 Hz), 2.02 – 1.86 (3H, m), 1.79 – 1.58 (11H, m), 1.56 – 1.43 (2H, m), 1.41 – 1.13 (18H, m), 0.99 – 0.86 (2H, m). HRMS (ESI) *m*/*z* calcd for $C_{30}H_{54}N_6O_4$ $[M+H]^+$ 563.4240, found 563.4275. Rt: 10.98 min.

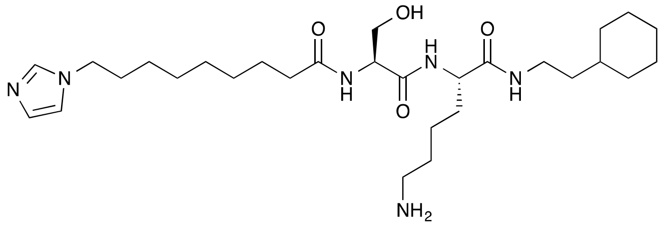

9-(1H-imidazol-1-yl)nonanoyl-Ser-Lys-2-cyclohexylethylamide (3)

This product was synthesised from **36** using the same method as compound **10** described above. The final product was a yellow oil (8 mg, Yield = 22%). ^{1}H NMR (500 MHz, MeOD) δ 8.98 (1H, s), 7.69 (1H, t, *J* = 1.8 Hz), 7.59 (1H, t, *J* = 1.8 Hz), 4.41–4.32 (2H, m), 4.32 – 4.24 (2H, m), 3.86 (1H, dd, *J* = 10.5, 5.6 Hz), 3.83 – 3.71 (2H, m), 3.28 – 3.18 (2H, m), 3.01 – 2.89 (2H, m), 2.33 – 2.25 (2H, m), 2.01 – 1.88 (3H, m), 1.80 – 1.60 (11H, m), 1.58 – 1.44 (2H, m), 1.44 – 1.14 (16H, m), 1.02 – 0.88 (2H, m). HRMS (ESI) *m*/*z* calcd for $C_{29}H_{52}N_6O_4$ $[M+H]^+$ 549.4084, found 549.4141. Rt: 10.24 min.

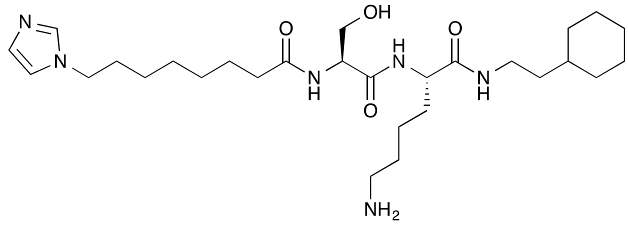

8-(1H-imidazol-1-yl)octanoyl-Ser-Lys-2-cyclohexylethylamide (4)

This product was synthesised from **35** using the same method as compound **10** described above. The final product was generated as a yellow oil (22 mg, Yield = 62%). ^{1}H NMR

(400 MHz, MeOD) δ 8.96 (1H, s), 7.67 (1H, br. s), 7.58 (1H, br. s), 4.41 – 4.20 (4H, m), 3.87 – 3.60 (2H, m), 3.25 – 3.12 (2H, m), 2.99 – 2.85 (2H, m), 2.33 – 2.21 (2H, m), 1.99 – 1.85 (3H, m), 1.80 – 1.58 (11H, m), 1.55 – 1.12 (16H, m), 1.01 – 0.84 (2H, m). HRMS (ESI) *m/z* calcd for $C_{28}H_{50}N_6O_4$ $[M+H]^+$ 535.3927, found 535.3992. Rt: 9.91 min.

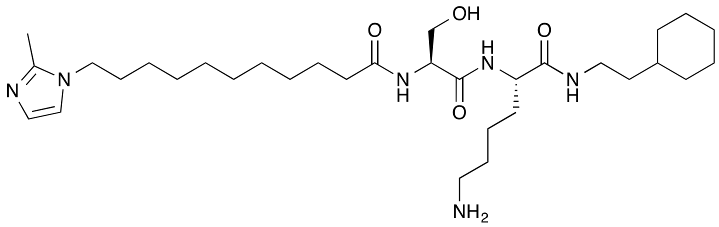

11-(2-Methyl-1H-imidazol-1-yl)undecanoyl-Ser-Lys-2-cyclohexylethylamide (5)
This product was synthesised from **42** using the same method as compound **10** described above. The final product was generated as a yellow oil (11 mg, Yield = 35%). ^{1}H NMR (400 MHz, MeOD) δ 8.29 (2H, s), 7.41 – 7.31 (1H, m), 7.30 – 7.16 (1H, m), 4.36 – 4.26 (2H, m), 4.05 (2H, t, *J* = 7.4 Hz), 3.81 (1H, dd, *J* = 10.4, 5.6 Hz), 3.76 – 3.63 (1H, m), 3.17 (2H, t, *J* = 7.3 Hz), 2.90 (2H, t, *J* = 6.8 Hz), 2.54 (3H, s), 2.27 – 2.18 (2H, m), 1.94 – 1.85 (1H, m), 1.84 – 1.75 (2H, m), 1.74 – 1.54 (10H, m), 1.51 – 1.41 (2H, m), 1.38 – 1.06 (19H, m), 0.98 – 0.81 (2H, m). HRMS (ESI) *m/z* calcd for $C_{32}H_{58}N_6O_4$ $[M+H]^+$ 591.4553, found 591.4589. Rt: 10.91 min.

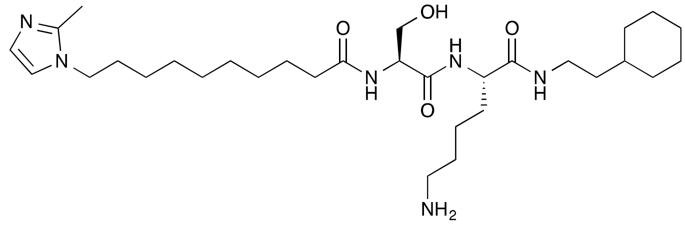

10-(2-Methyl-1H-imidazol-1-yl)decanoyl-Ser-Lys-2-cyclohexylethylamide (6)
This product was synthesised from **41** using the same method as compound **10** described above. The final product was generated as a yellow oil (5 mg, Yield = 12%). ^{1}H NMR (500 MHz, MeOD) δ 8.36 (1H, s), 7.31 (1H, s), 7.19 (1H, s), 4.39 – 4.32 (2H, m), 4.05 (2H, t, *J* = 7.3 Hz), 3.84 (1H, dd, *J* = 10.4, 5.6 Hz), 3.76 – 3.71 (2H, m), 3.24 – 3.17 (2H, m), 2.94 (2H, td, *J* = 7.8, 2.7 Hz), 2.53 (3H, s), 2.30 – 2.23 (2H, m), 2.02 – 1.92 (1H, m), 1.91 – 1.85 (1H, m), 1.86 – 1.77 (2H, m), 1.76 – 1.58 (11H, m), 1.54 – 1.43 (2H, m), 1.41 – 1.12 (18H, m), 0.97 – 0.88 (2H, m). HRMS (ESI) *m/z* calcd for $C_{31}H_{56}N_6O_4$ $[M+H]^+$ 577.4397, found 577.4424. Rt: 10.68 min.

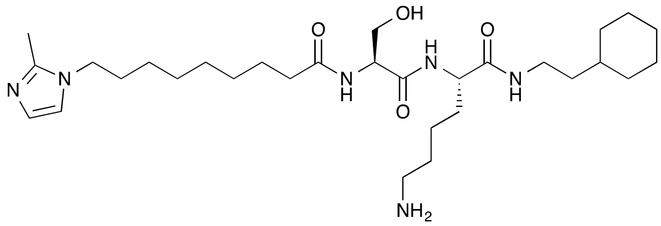

9-(2-Methyl-1H-imidazol-1-yl)nonanoyl-Ser-Lys-2-cyclohexylethylamide (7)

This product was synthesised from **40** using the same method as compound **10** described above. The final product was generated as a yellow oil (11 mg, Yield = 30%). HRMS (ESI) *m*/*z* calcd for $C_{30}H_{54}N_6O_4$ $[M+H]^+$ 563.4240, found 563.4313, Rt: 10.45 min.

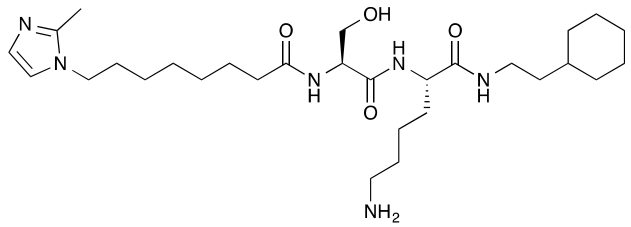

8-(2-Methyl-1H-imidazol-1-yl)octanoyl-Ser-Lys-2-cyclohexylethylamide (8)

This product was synthesised from **39** using the same procedure as compound **10** described above. The final product was generated as a yellow oil (1.72 mg, Yield = 5%). ^{1}H NMR (400 MHz, MeOD) δ 8.37 (1H, s), 7.29 (1H, d, *J* = 1.9 Hz), 7.17 (1H, d, *J* = 1.9 Hz), 4.37 – 4.26 (2H, m), 4.02 (2H, t, *J* = 7.4 Hz), 3.81 (1H, dd, *J* = 10.4, 5.6 Hz), 3.73 – 3.64 (1H, m), 3.17 (2H, t, *J* = 7.3 Hz), 2.94 – 2.85 (2H, m), 2.50 (3H, s), 2.24 (2H, t, *J* = 7.5 Hz), 1.99 – 1.86 (1H, m), 1.84 – 1.54 (14H, m), 1.53 – 1.10 (17H, m), 0.96 – 0.81 (2H, m). HRMS (ESI) *m*/*z* calcd for $C_{29}H_{52}N_6O_4$ $[M+H]^+$ 549.4084, found 549.4131. Rt: 9.77 min.

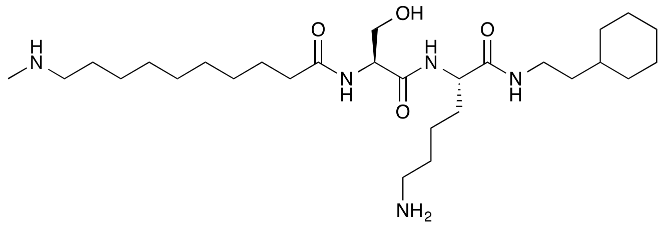

(10-Methylaminodecanoyl)-Ser-Lys-2-cyclohexylethylamide (13)

Using **43** (61.4 mg) and HATU (116 mg), **13** was synthesized using the same procedure as compound **10**. The final product (**13**) was a yellow oil (4.6 mg, Yield = 12%). ^{1}H NMR (500 MHz, MeOD) δ 8.13 (1H, s), 4.35 (1H, dd, *J* = 9.9, 4.5 Hz), 4.30 (1H, t, *J* = 5.7 Hz), 3.87 – 3.76 (3H, m), 3.24 (2H, t, *J* = 7.4 Hz), 3.04 – 2.97 (2H, m), 2.98 – 2.90 (2H, m), 2.72 (3H, s), 2.30 (2H, t, *J* = 7.6 Hz), 2.05 – 1.92 (1H, m), 1.81 – 1.60 (12H, m), 1.56 – 1.46 (2H, m), 1.46 –1.15 (18H, m), 1.01 – 0.88 (2H, m). HRMS (ESI) *m*/*z* calcd for $C_{29}H_{52}N_6O_4$ $[M+H]^+$ 526.4288, found 526.4322. Rt: 7.47 min.

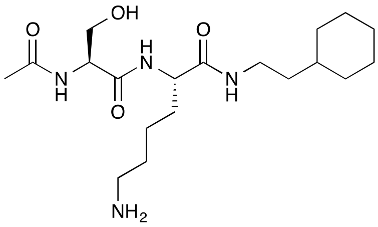

Acetyl-Ser-Lys-2-cyclohexenylethylamide (14)

Following the removal of the Fmoc group using 20% piperidine/DMF, Ser(*t*-Bu)-Lys(Boc)-hydrazinobenzoyl resin was treated with a mixture of acetic anhydride (10% *v*/*v*), DIPEA (20% *v*/*v*) in DMF (3 mL) for 1 h at room temperature. The resin was washed, dried and cleaved oxidatively using the same method as compound **10** to afford compound **14**. Following purification, **14** was generated as a yellow oil (8 mg, Yield = 38%,). ^{1}H NMR (400 MHz, MeOD) δ 8.46 (1H, s), 4.37 – 4.25 (2H, m), 3.81 (1H, dd, *J* = 10.4, 5.6 Hz), 3.74 – 3.64 (2H, m), 3.17 (2H, t, *J* = 7.3 Hz), 2.90 (1H, td, *J* = 7.8, 2.4 Hz), 2.02 – 1.87 (4H, m), 1.75 – 1.57 (8H, m), 1.54 – 1.41 (2H, m), 1.39 – 1.30 (2H, m), 1.30 – 1.07 (5H, m), 0.97 – 0.79 (2H, m). HRMS (ESI) *m*/*z* calcd for $C_{19}H_{36}N_4O_4$ [M+H]$^+$ 385.2770, found 385.2811. Rt: 11.02 min.

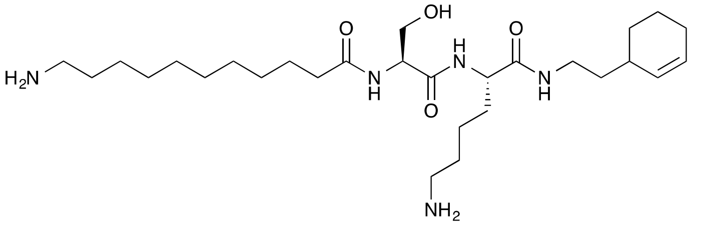

(11-Aminoundecanoyl)-Ser-Lys-2-cyclohexenylethylamide (15)

This product was synthesised from commercially available 2-(1-cyclohexenyl)- ethanamine using the same method as compound **9**. The final product was a yellow oil (10.2 mg, Yield = 15%). ^{1}H NMR (400 MHz, MeOD) δ 5.52 (1H, m), 4.67 – 4.56 (1H, m), 4.34 (1H, td, *J* = 5.8, 2.1 Hz), 3.81 (1H, dd, *J* = 10.6, 5.7 Hz), 3.79 – 3.70 (1H, m), 3.67 – 3.51 (2H, m), 2.95 – 2.84 (4H, m), 2.24 (3H, t, *J* = 7.7 Hz), 2.12 – 2.07 (1H, m), 2.04 – 1.97 (2H, m), 1.97 – 1.84 (4H, m), 1.74 – 1.52 (14H, m), 1.50 – 1.40 (2H, m), 1.38 – 1.24 (14H, m). HRMS (ESI) *m*/*z* calcd for $C_{28}H_{53}N_5O_4$ [M+H]$^+$ 524.4131, found 524.4177. Rt: 8.39 min.

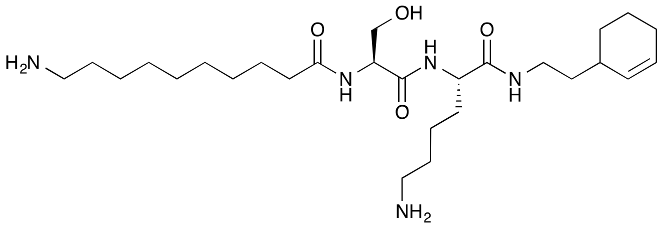

(10-Aminononanoyl)-Ser-Lys-2-cyclohexenylethylamide (16)

This product was synthesised from commercially available 2-(1-cyclohexenyl)- ethanamine using the same method as compound **10** described above. The final product was generated as a yellow oil (11 mg, Yield = 37%). ^{1}H NMR (500 MHz, MeOD) δ 4.46 – 4.30 (2H, m), 3.87 (1H, dd*, J* = 10.8, 5.4 Hz), 3.84 – 3.58 (3H, m), 3.05 – 2.89 (5H, m), 2.36 – 2.19 (4H, m), 2.19 – 2.10 (1H, m), 2.04 – 1.92 (3H, m), 1.91 – 1.44 (21H, m), 1.44 – 1.33 (11H, m). HRMS (ESI*) m/*z calcd for $C_{27}H_{51}N_5O_4$ [M+H]$^+$ 510.3975, found 510.4022. Rt: 7.46 min.

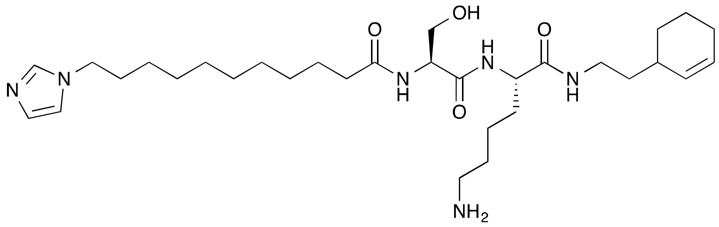

11-(1H-imidazol-1-yl)undecanoyl-Ser-Lys-2-cyclohexenylethylamide (17)

This product was synthesised from commercially available 2-(1-cyclohexenyl)- ethanamine using the same method as compound **10** described above. The final product was a brown oil (10.2 mg Yield = 33%,). ^{1}H NMR (400 MHz, MeOD) δ 8.95 (1H, s), 7.71 – 7.62 (1H, m), 7.61 – 7.50 (1H, m), 4.41 – 4.28 (1H, m), 4.25 (2H, t*, J* = 7.3 Hz), 3.86 (1H, dd*, J* = 9.7, 4.7 Hz), 3.81 – 3.70 (1H, m), 3.03 – 2.86 (4H, m), 2.29 – 2.23 (3H, m), 2.06 – 1.85 (5H, m), 1.81 – 1.42 (20H, m), 1.38 – 1.25 (14H, m). HRMS (ESI*) m/*z calcd for $C_{31}H_{54}N_6O_4$ $[M+H]^+$ 575.4240, found 575.4303, Rt: 8.28 min.

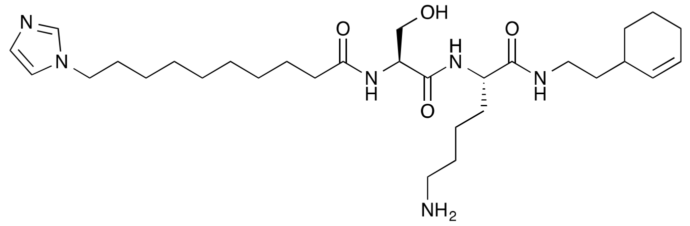

10-(1H-imidazol-1-yl)decanoyl-Ser-Lys-2-cyclohexenylethylamide (18)

This product was synthesised from commercially available 2-(1-cyclohexenyl)- ethanamine using the same method as compound **10** described above. The final product **(18**) was a yellow oil (9 mg, Yield = 30%). ^{1}H NMR (500 MHz, MeOD) δ 8.97 (1H, s), 8.10 (1H, s), 7.67 (1H, s), 7.58 (1H, s), 4.44 – 4.31 (2H, m), 4.26 (2H, t*, J* = 7.3 Hz), 3.85 (1H, dd*, J* = 11.0, 5.4 Hz), 3.85 – 3.54 (2H, m), 3.02 – 2.91 (3H, m), 2.32 – 2.02 (6H, m), 2.02 – 1.87 (5H, m), 1.86 – 1.75 (1H, m), 1.74 – 1.43 (15H, m), 1.41 – 1.31 (11H, m). HRMS (E*SI) m/z* calcd for $C_{29}H_{52}N_6O_4$ $[M+H]^+$ 561.4084, found 561.4126. Rt: 10.25 min.

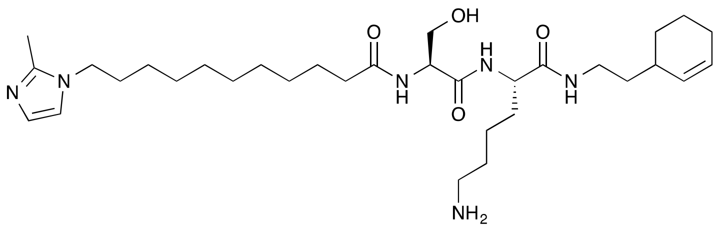

11-(2-Methyl-1H-imidazol-1-yl)undecanoyl-Ser-Lys-2-cyclohexenylethylamide (19)

This product was synthesised from commercially available 2-(1-cyclohexenyl)- ethanamine using the same method as compound **10** described above. The final produ**c**t was a yellow oil (14 mg, Yield = 43%). ^{1}H NMR (400 MHz, MeOD) δ 8.37 (2H, s), 7.39 (1H, d, *J* = 1.9 Hz), 7.28 (1*H*, d, *J* = 1.9 Hz), 4.37 – 4.28 (2H, m), 4.14 – 4.02 (2H, m), 3.82 – 3.68 (2H, m), 2.98 – 2.90 (3H, m), 2.66 (3H, s), 2.57 (2H, m), 2.35 – 2.14 (5H, m), 2.02 – 1.87 (2H, m), 1.87 – 1.76 (3H, m), 1.74 – 1.42 (14H, m), 1.40 – 1.26 (14H, m). HRMS (E*SI) m/z* calcd for $C_{32}H_{56}N_6O_4$ $[M+H]^+$ 589.4397, found 589.4436. Rt: 10.90 min.

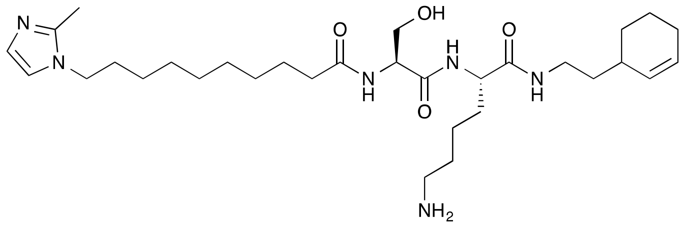

10-(2-Methyl-1H-imidazol-1-yl)decanoyl-Ser-Lys-2-cyclohexenylethylamide (20)

This product was synthesised from commercially available 2-(1-cyclohexenyl)- ethanamine using the same method as compound **10** described above. The final product was a yellow oil (13 mg, Yield = 43%). ^{1}H NMR (500 MHz, MeOD) δ 7.53 (1H, d, *J* = 1.7 Hz), 7.44 (1H, d, *J* = 1.7 Hz), 4.46 – 4.32 (2H, m), 4.15 (2H, *t*, *J* = 7.4 Hz), 3.87 (1H, dd, *J* = 10.7, 5.3 Hz), 3.85 – 3.60 (3H, m), 3.04 – 2.91 (3H, m), 2.66 (3H, s), 2.34 – 2.20 (4H, m), 2.18 – 2.03 (2H, m), 2.02 – 1.81 (6H, m), 1.79 – 1.44 (16H, m), 1.43 – 1.34 (10H, m). HRMS (E*SI) m/z* calcd for $C_{31}H_{54}N_6O_4$ $[M+H]^+$ 575.4240, found 575.4307. Rt: 10.32 min.

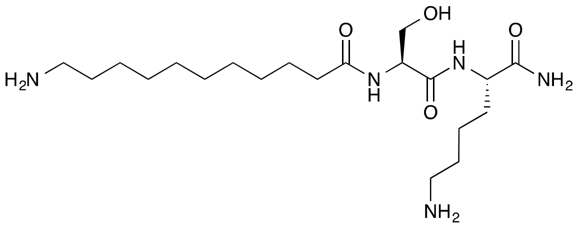

(11-Aminoundecanoyl)-Ser-Lys-NH$_2$ (21)

This product was synthesised using Rink amide resin (Loading: 0.64 mmol/g, 100 mg) using Fmoc chemistry. Removal of deprotecting groups was achieved by treatment with TFA/TIPS/H_2O for 1 h (2 mL, 95:2.5:2.5). The final product was a yellow oil (20 mg, Yield = 62%). ^{1}H NMR (400 MHz, MeOD) δ 8.54 – 8.38 (1H, s), 4.45 – 4.29 (2H, m), 3.84 (1H, dd, *J* = 10.5, 5.6 Hz), 3.79 –3.68 (1H, m), 3.24 – 3.17 (1H, q, *J* = 7.4 Hz), 2.98 – 2.85 (5H, m), 2.33 – 2.20 (2H, m), 2.03 – 1.90 (1H, m), 1.76 – 1.55 (8H, m), 1.57 – 1.44 (2H, m), 1.43 – 1.26 (16H, m). HRMS (ESI) *m/z* calcd for $C_{20}H_{41}N_5O_4$ $[M+H]^+$ 416.3192, found 416.3229. Rt: 9.90 min.

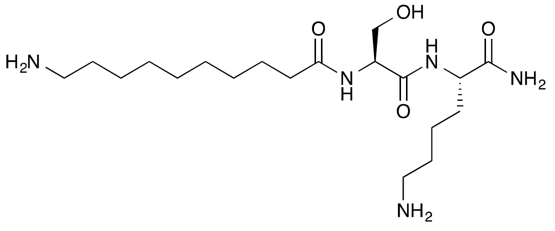

(10-Aminodecanoyl)-Ser-Lys-NH$_2$ (22)

This product was synthesised in the same manner as compound **21**. The final product was generated as a yellow oil (22 mg, Yield = 70%). ^{1}H NMR (400 MHz, MeOD) δ 4.38 – 4.31 (2H, m), 3.81 (1H, dd, *J* = 10.6, 5.6 Hz), 3.76 – 3.66 (1H, m), 2.94 – 2.85 (4H,m), 2.24 (2*H*, t, *J* = 7.6 Hz), 2.01–1.87 (1H, m), 1.75 – 1.54 (7H, m), 1.53 – 1.41 (3H, m), 1.40 – 1.18 (11H, m). HRMS (ESI) *m/z* calcd for $C_{19}H_{39}N_5O_4$ $[M+H]^+$ 402.3036, found 402.3070. Rt: 6.78 min.

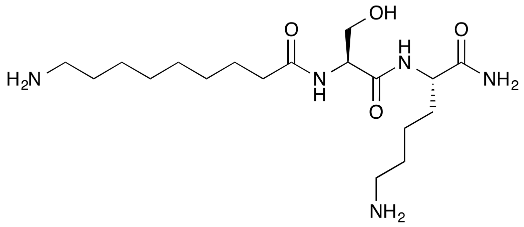

(9-Aminononanoyl)-Ser-Lys-NH$_2$ (23)

This product was synthesised in the same manner as compound **21**. The final product was generated as a yellow oil (24 mg, Yield = 78%). HRMS (ESI) *m/z* calcd for $C_{18}H_{37}N_5O_4$ $[M+H]^+$ 388.2879, found 388.2911. Rt: 1.21 min.

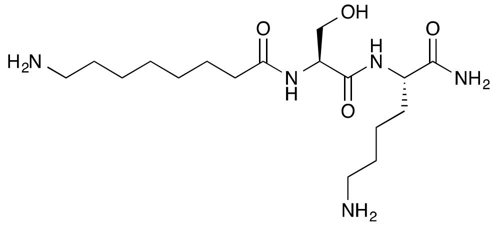

(8-Aminooctanoyl)-Ser-Lys-NH$_2$ (24)

This product was synthesised in the same manner as compound **21**. The final product was generated as a yellow oil (23 mg, Yield = 77%). ^{1}H NMR (400 MHz, MeOD) δ 8.34 (1H, s), 4.38 – 4.27 (2H, m), 3.86 – 3.75 (1H, m), 3.75 – 3.66 (1H, m), 2.95 – 2.84 (4H, m), 2.30 – 2.21 (2H, m), 1.99 –1.88 (1H, m), 1.73 – 1.56 (7H, m), 1.54 – 1.40 (2H, m), 1.41 – 1.21 (7H, m). HRMS (ESI) *m/z* calcd for $C_{17}H_{35}N_5O_4$ $[M+H]^+$ 374.2723, found 374.2763. Rt: 1.06 min.

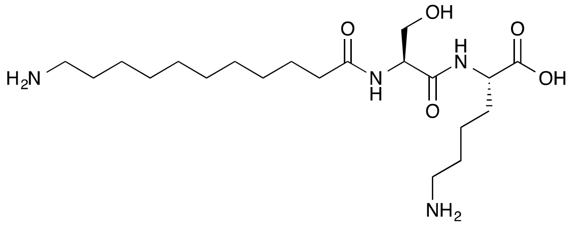

(11-Aminoundecanoyl)-Ser-Lys-OH (25)

Using Wang resin (Loading: 0.67 mmol/g, 100 mg), **32** and previously described Fmoc chemistry, this product was generated as a yellow oil. HRMS (ESI) *m/z* calcd for $C_{20}H_{40}N_4O_5$ $[M+H]^+$ 417.3032, found 417.3085. Rt: 2.68 min.

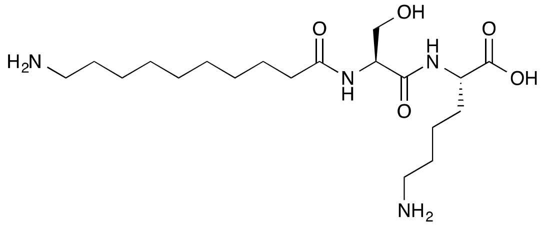

(10-Aminodecanoyl)-Ser-Lys-OH (26)

Using Wang resin (Loading: 0.67 mmol/g, 100 mg), **30** and previously described Fmoc chemistry, this product was generated as a yellow oil (9.9 mg, Yield = 30%). ^{1}H NMR (400 MHz, MeOD) δ 8.42 (1H, s), 4.40 (1H, t, *J* = 5.4 Hz), 4.24 (1H, dd, *J* = 7.5, 5.0 Hz), 3.82 (1H, dd, *J* = 11.2, 5.4 Hz), 3.76 (1H, dd, *J* = 11.2, 5.4 Hz), 2.92 (4H, t, *J* = 7.6Hz), 2.29

(2H, td, $J = 7.3$, 2.2 Hz), 1.98 – 1.83 (1H, m), 1.81 – 1.55 (7H, m), 1.52 – 1.27 (13H, m). HRMS (ESI) *m/z* calcd for $C_{19}H_{38}N_4O_5$ $[M+H]^+$ 403.2876 found, 403.2920. Rt: 4.69 min.

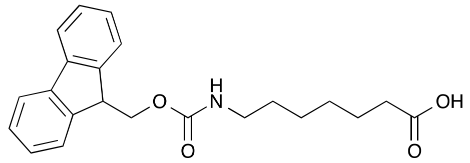

7-(9H-fluoren-9-ylmethoxycarbonylamino)heptanoic acid (45)

Commercially available 7-aminoheptanoic acid (0.5 g, 3.46 mmol) was dissolved in 10% Na_2CO_3 (36 mL) and 1,4-dioxane (24 mL); Fmoc-Cl (1.5 eq) was sunsequently added in portions. The reaction mixture was left stirring overnight at room temperature and extracted with diethylether (2×40 mL). The aqueous layer was acidified with 2 M HCl to pH1. The white solid was filtered, washed with water and dried in a desiccator to give the desired product as a white solid (1.18 g, Yield = 93%). ^{1}H NMR (400 MHz, MeOD) δ 7.77 (2H, d, $J = 7.5$ Hz), 7.63 (2H, d, $J = 7.5$ Hz), 7.37 (2H, t, $J = 7.5$ Hz), 7.28 (2H, t, $J = 7.5$ Hz), 4.51 (1H, s), 4.33 (2H, d, $J = 6.9$ Hz), 4.18 (1H, t, $J = 6.9$ Hz), 3.07 (2H, t, $J = 7.0$ Hz), 2.26 (2H, t, $J = 7.4$ Hz), 1.58 (2H, p, $J = 7.4$ Hz), 1.46 (2H, p, $J = 7.0$ Hz), 1.32 (4H, m). HRMS (ESI) *m/z* calcd for $C_{22}H_{25}NO_4$ $[M+H]^+$ 368.1817, found 368.1860.

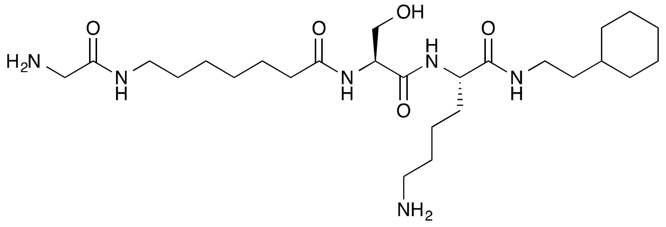

Gly-(7-aminoheptanoyl)-Ser-Lys-2-cyclohexylethylamide (27).

Using standard Fmoc chemistry, **45** (168 mg, 0.46 mmol) and Fmoc-Gly-OH (137 mg, 5 eq) were coupled to Fmoc-Ser(*t*-Bu)-Lys(Boc)-hydrazinobenzoyl resin (150 mg) as previously described. Cleavage was carried out using excess **31** (78 mg, 0.42 mmol). The final product was achieved as a yellow oil (1.8 mg, Yield = 3%). HRMS (ESI) *m/z* calcd for $C_{26}H_{50}N_6O_5$ $[M+Na]^+$ 549.3735, found 549.3724. Rt: 10.24 min.

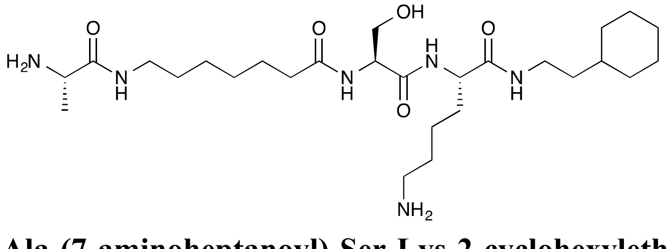

Ala-(7-aminoheptanoyl)-Ser-Lys-2-cyclohexylethylamide (28)

This product was synthesised in the same manner as **27** using Fmoc-Ala-OH (143 mg). The final product was achieved as yellow oil (2.6 mg, Yield = 5%). HRMS (ESI) *m/z* calcd for $C_{27}H_{52}N_6O_5$ $[M+Na]^+$ 563.3891, found 563.3875. Rt: 10.28 min.

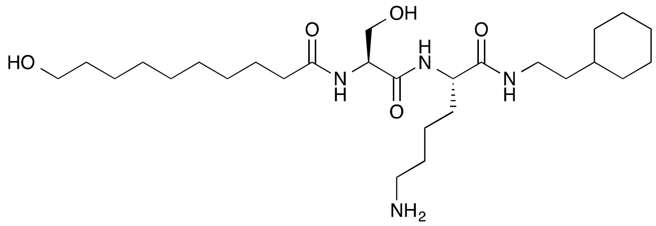

(10-Hydroxydecanoyl)-Ser-Lys-2-cyclohexylethylamide (46)

This product was synthesised from commercially available 10-hydroxydecanoic acid (86.6 mg) in the same manner as described for compound **10** above. The final product was achieved as a white solid (6.2 mg, Yield = 12%). ^{1}H NMR (400 MHz, MeOD) δ 4.39 – 4.29 (2H, m), 3.83 (1H, dd, *J* = 10.3, 5.8 Hz), 3.75 – 3.68 (1H, m), 3.64 (5H, s), 3.54 (2H, t, *J* = 6.6 Hz), 3.19 (2H, t, *J* = 7.3 Hz), 2.93 (2H, td, *J* = 7.4, 2.1 Hz), 2.26 (2H, t, *J* = 7.6 Hz), 2.04 – 1.88 (1H, m), 1.78 – 1.57 (10H, m), 1.56 – 1.41 (4H, m), 1.41 – 1.11 (16H, m), 0.99 – 0.84 (2H, m). HRMS (ESI) *m/z* calcd for $C_{27}H_{52}N_4O_5$ $[M+H]^+$ 513.3971, found 513.4004. Rt: 12.71 min.

1.2. ^{1}H NMR of peptidomimetics.

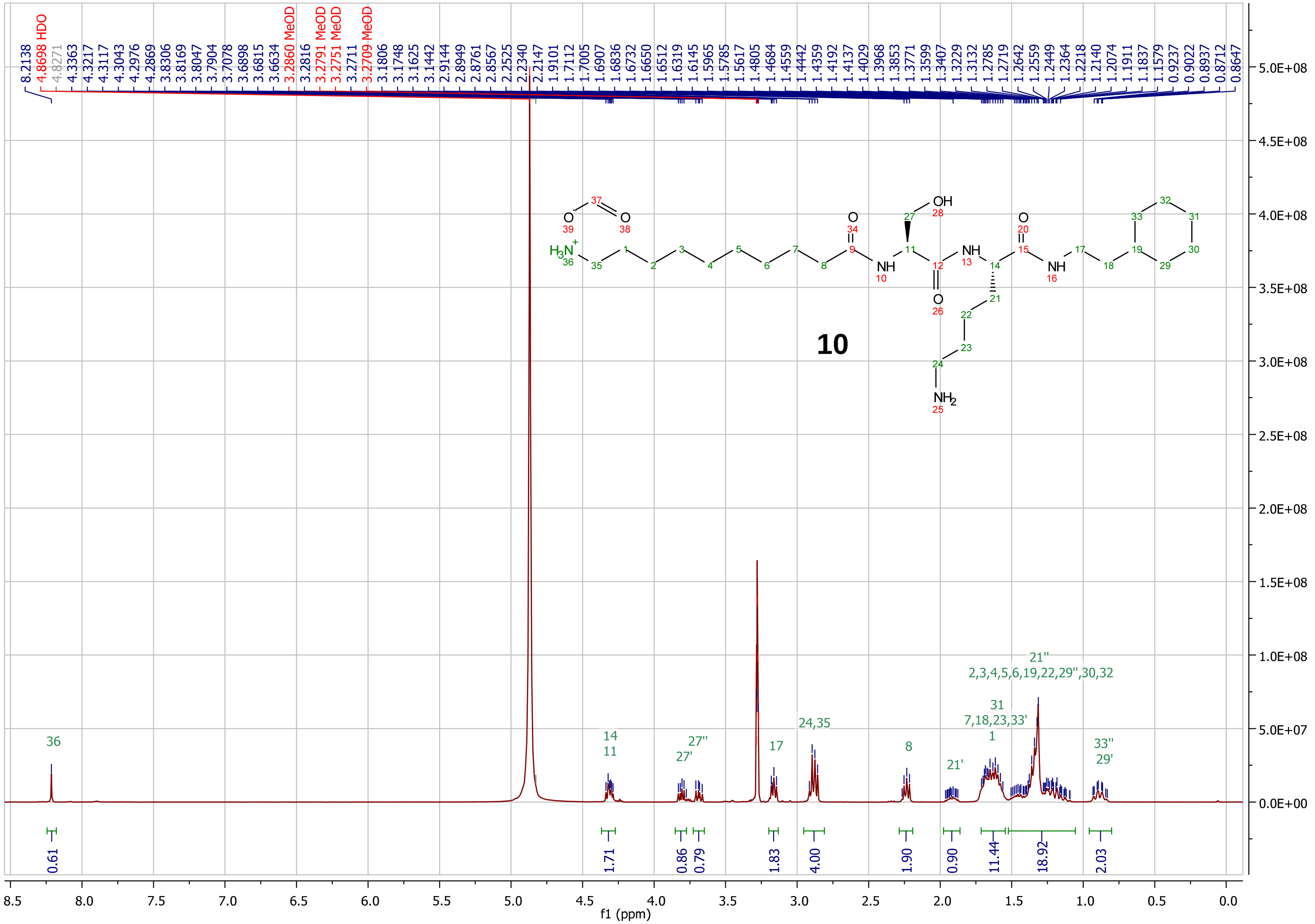

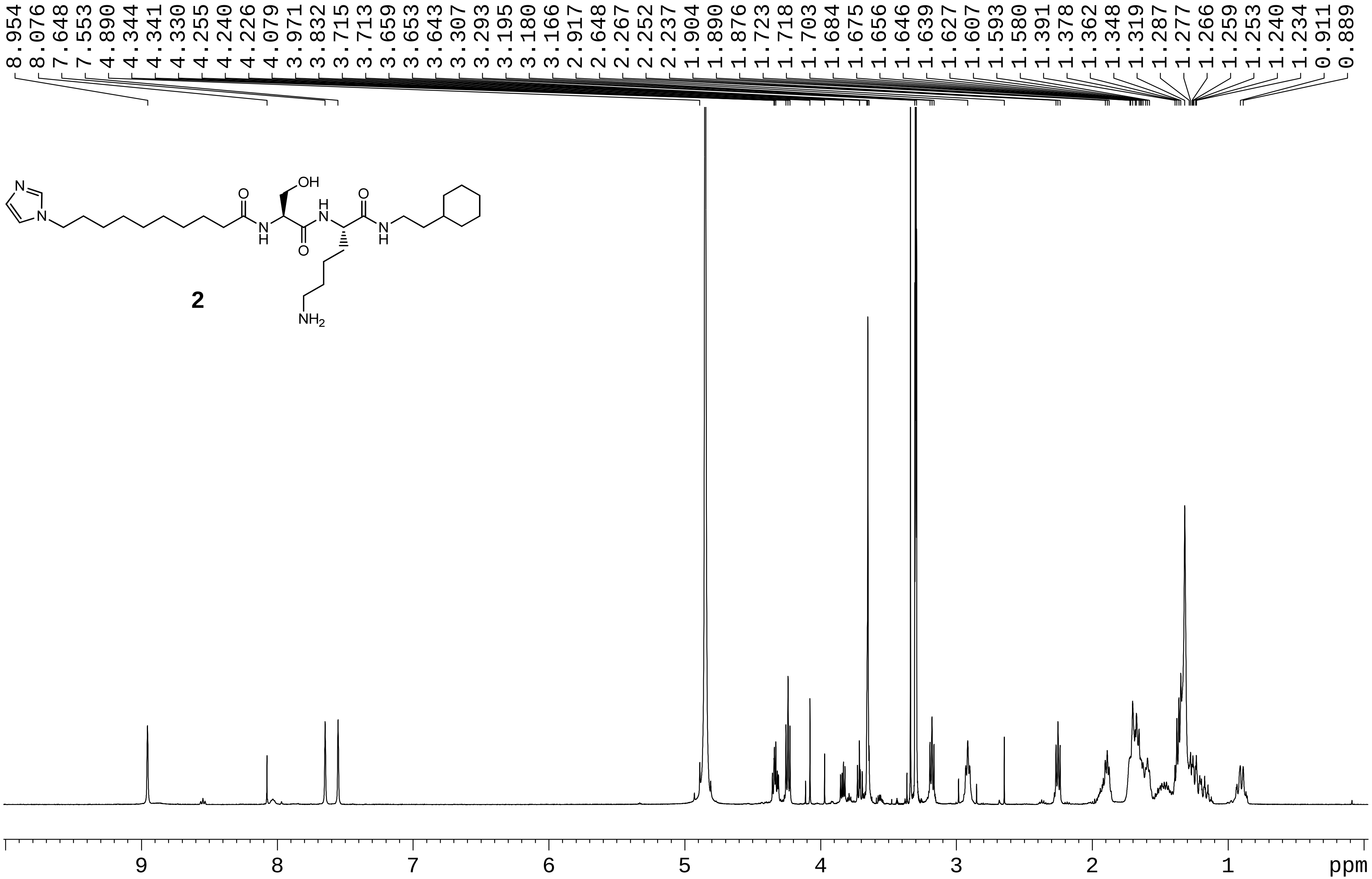
8.954 8.076 7.648 7.553 4.890 4.344 4.341 4.330 4.255 4.240 4.226 4.079 3.971 3.832 3.715 3.713 3.659 3.653 3.643 3.307 3.293 3.195 3.180 3.166 2.917 2.648 2.267 2.252 2.237 1.904 1.890 1.876 1.723 1.718 1.703 1.684 1.675 1.656 1.646 1.639 1.627 1.607 1.593 1.580 1.391 1.378 1.362 1.348 1.319 1.287 1.277 1.266 1.259 1.253 1.240 1.234 0.911 0.889
N
N
O
OH
H
N
O
N
H
O
N
H
NH2
2
9
8
7
6
5
4
3
2
1
ppm

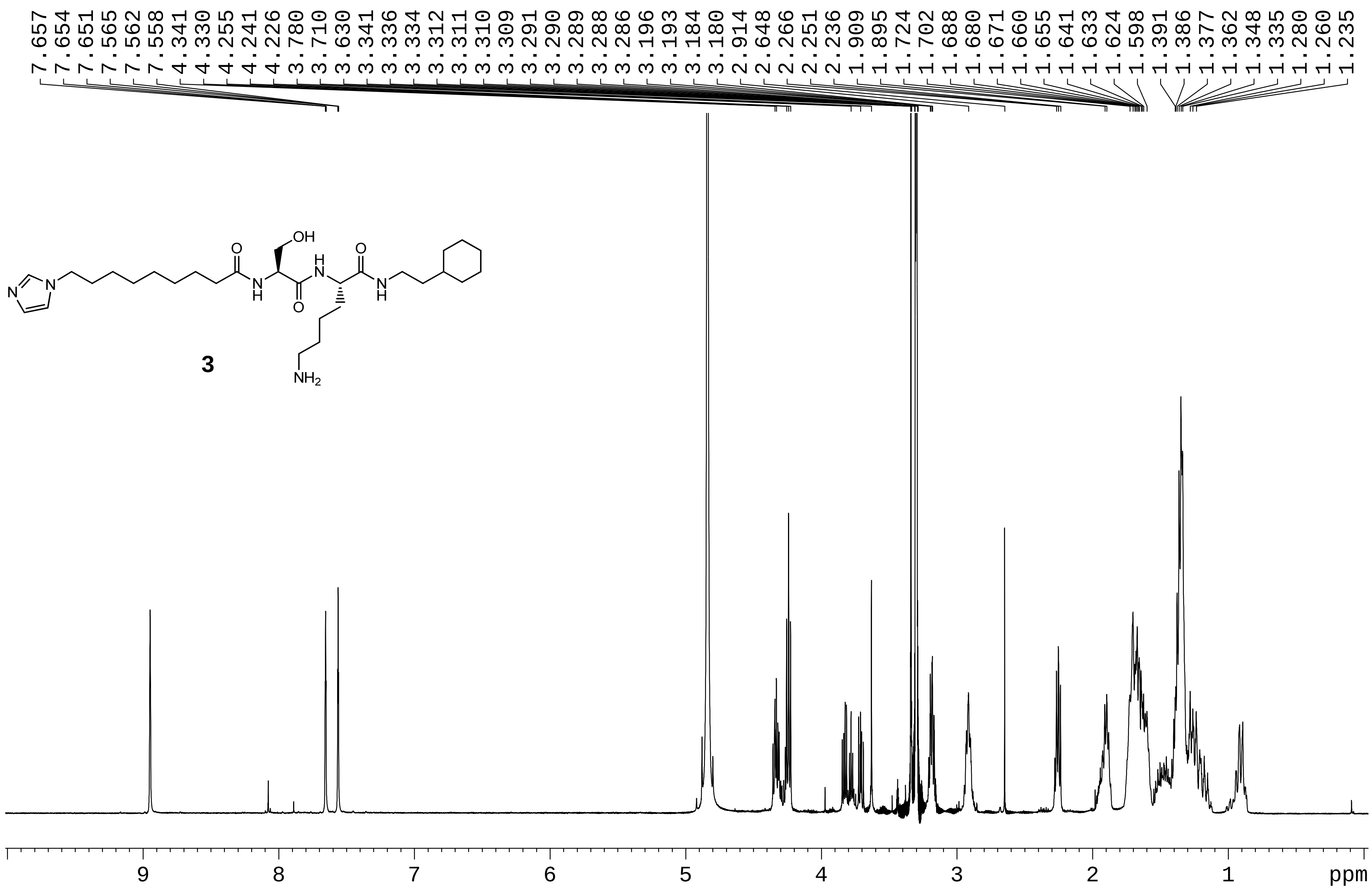
7.657 7.654 7.651 7.565 7.562 7.558 4.341 4.330 4.255 4.241 4.226 3.780 3.710 3.630 3.341 3.336 3.334 3.312 3.311 3.310 3.309 3.291 3.290 3.289 3.288 3.286 3.196 3.193 3.184 3.180 2.914 2.648 2.266 2.251 2.236 1.909 1.895 1.724 1.702 1.688 1.680 1.671 1.660 1.655 1.641 1.633 1.624 1.598 1.391 1.386 1.377 1.362 1.348 1.335 1.280 1.260 1.235
OH
O
N
N
H
N
H
O
O
N
H
NH2
3
9
8
7
6
5
4
3
2
1
ppm

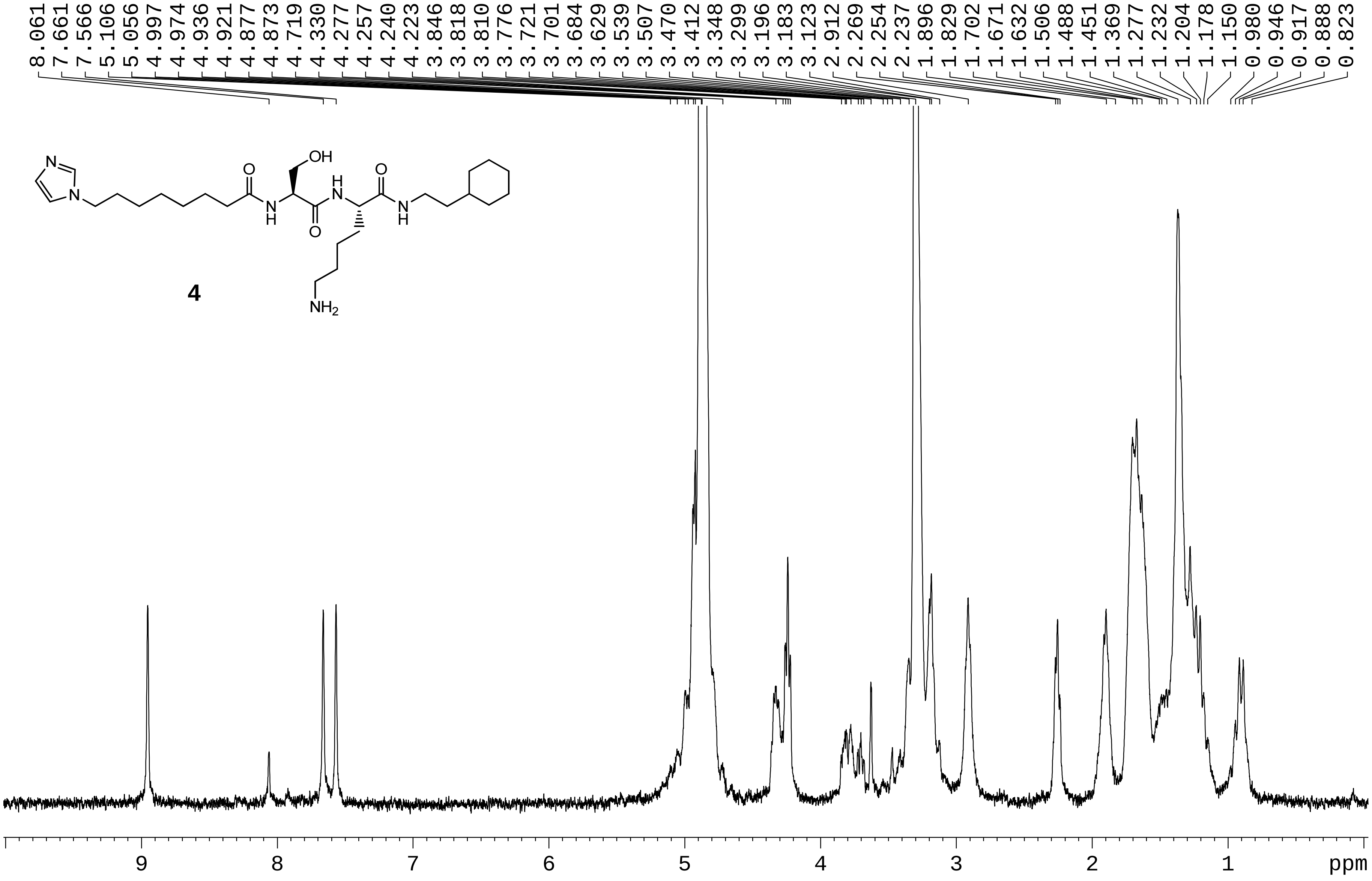
8.061
7.661
7.566
5.106
5.056
4.997
4.974
4.936
4.921
4.877
4.873
4.719
4.330
4.277
4.257
4.240
4.223
3.846
3.818
3.810
3.776
3.721
3.701
3.684
3.629
3.539
3.507
3.470
3.412
3.348
3.299
3.196
3.183
3.123
2.912
2.269
2.254
2.237
1.896
1.829
1.702
1.671
1.632
1.506
1.488
1.451
1.369
1.277
1.232
1.204
1.178
1.150
0.980
0.946
0.917
0.888
0.823
N
N
O
OH
H
N
O
N
H
N
H
O
NH2
4
9
8
7
6
5
4
3
2
1
ppm

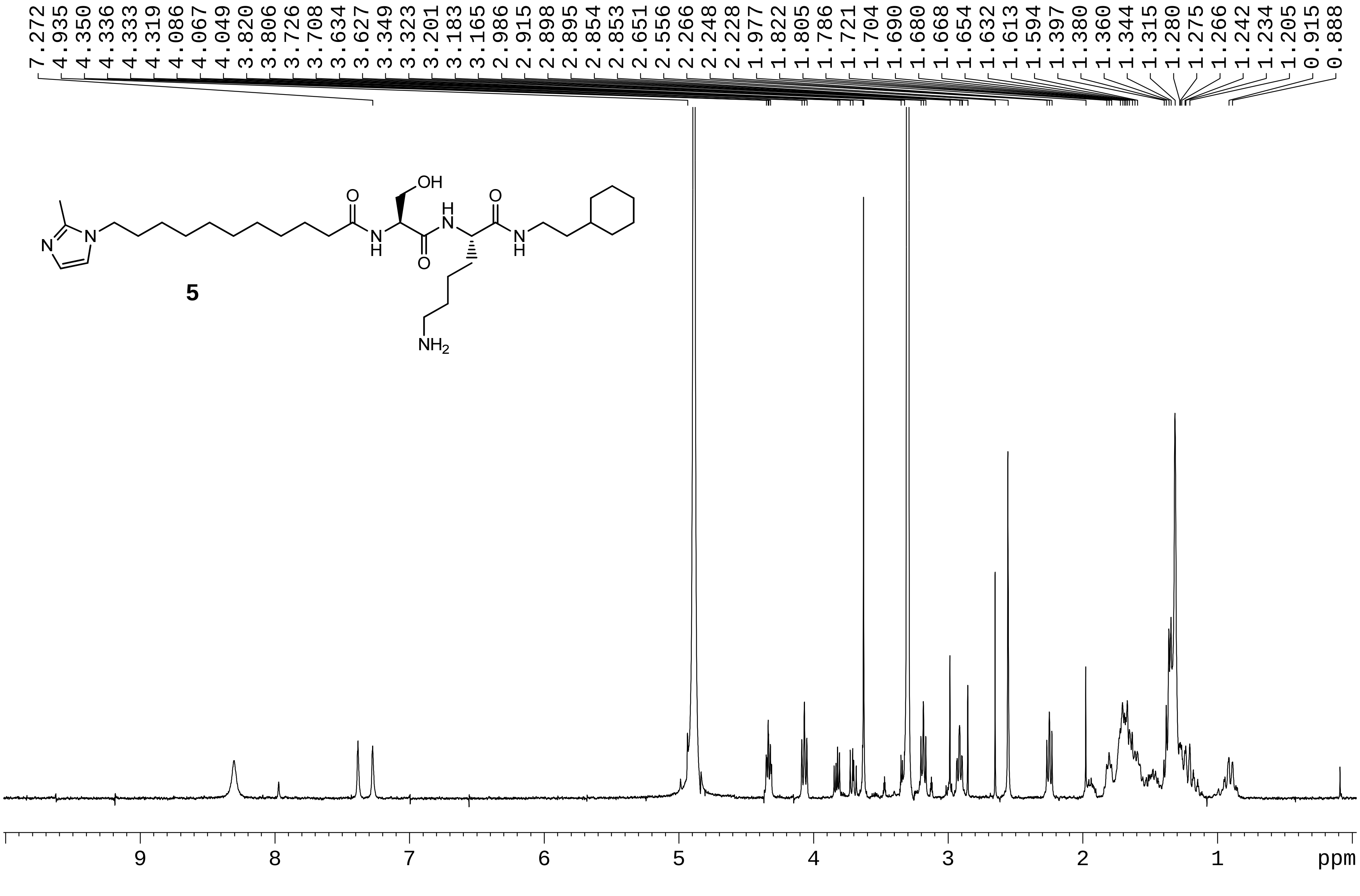
7.272
4.935
4.350
4.336
4.333
4.319
4.086
4.067
4.049
3.820
3.806
3.726
3.708
3.634
3.627
3.349
3.323
3.201
3.183
3.165
2.986
2.915
2.898
2.895
2.854
2.853
2.651
2.556
2.266
2.248
2.228
1.977
1.822
1.805
1.786
1.721
1.704
1.690
1.680
1.668
1.654
1.632
1.613
1.594
1.397
1.380
1.360
1.344
1.315
1.280
1.275
1.266
1.242
1.234
1.205
0.915
0.888
OH
O
N
N
H
O
H
N
O
N
H
NH2
5
9
8
7
6
5
4
3
2
1
ppm

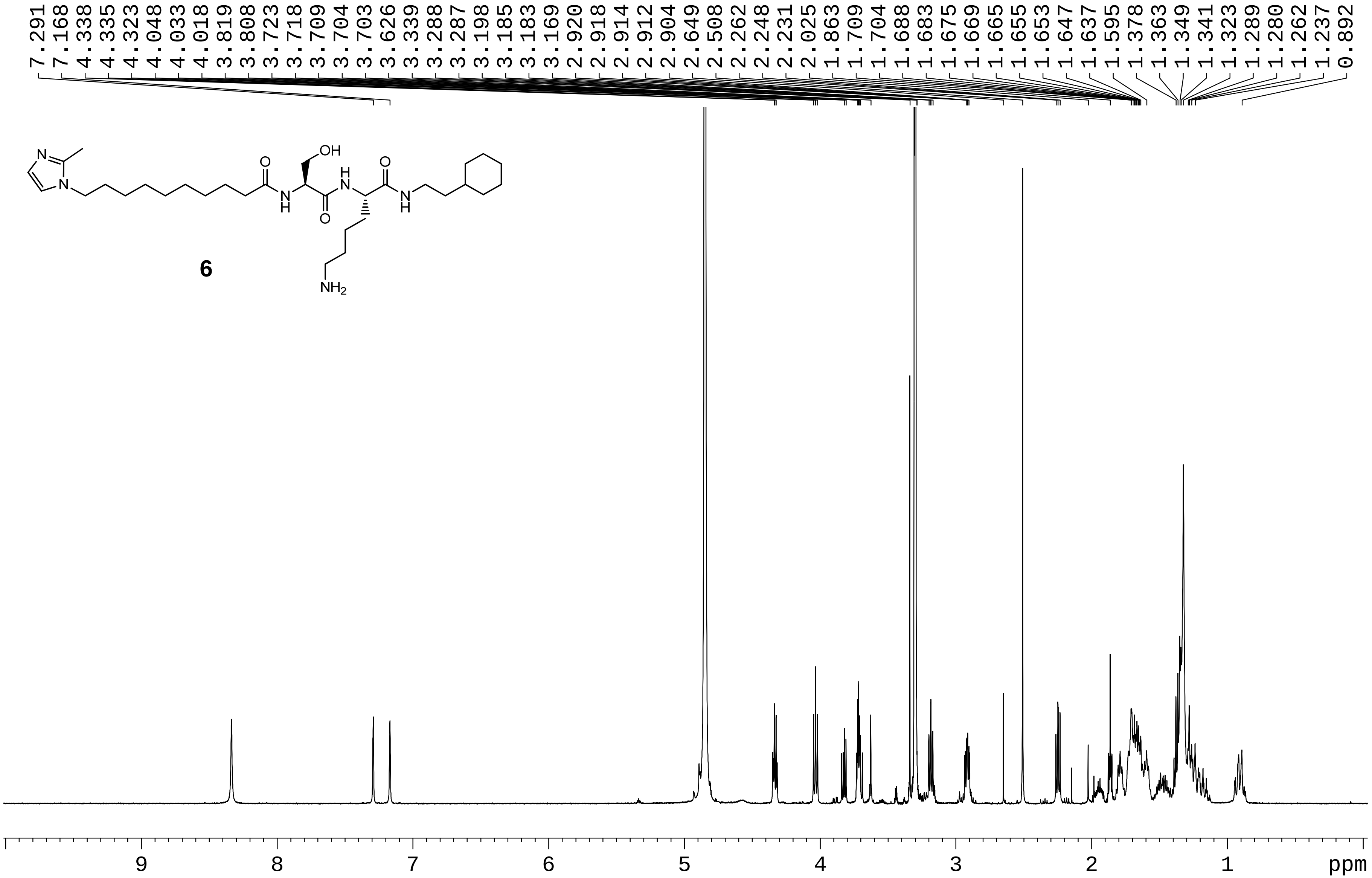
7.291
7.168
4.338
4.335
4.323
4.048
4.033
4.018
3.819
3.808
3.723
3.718
3.709
3.704
3.703
3.626
3.339
3.288
3.287
3.198
3.185
3.183
3.169
2.920
2.918
2.914
2.912
2.904
2.649
2.508
2.262
2.248
2.231
2.025
1.863
1.709
1.704
1.688
1.683
1.675
1.669
1.665
1.655
1.653
1.647
1.637
1.595
1.378
1.363
1.349
1.341
1.323
1.289
1.280
1.262
1.237
0.892
OH
N
N
O
H
N
H
N
O
O
H
N
NH2
6
9
8
7
6
5
4
3
2
1
ppm

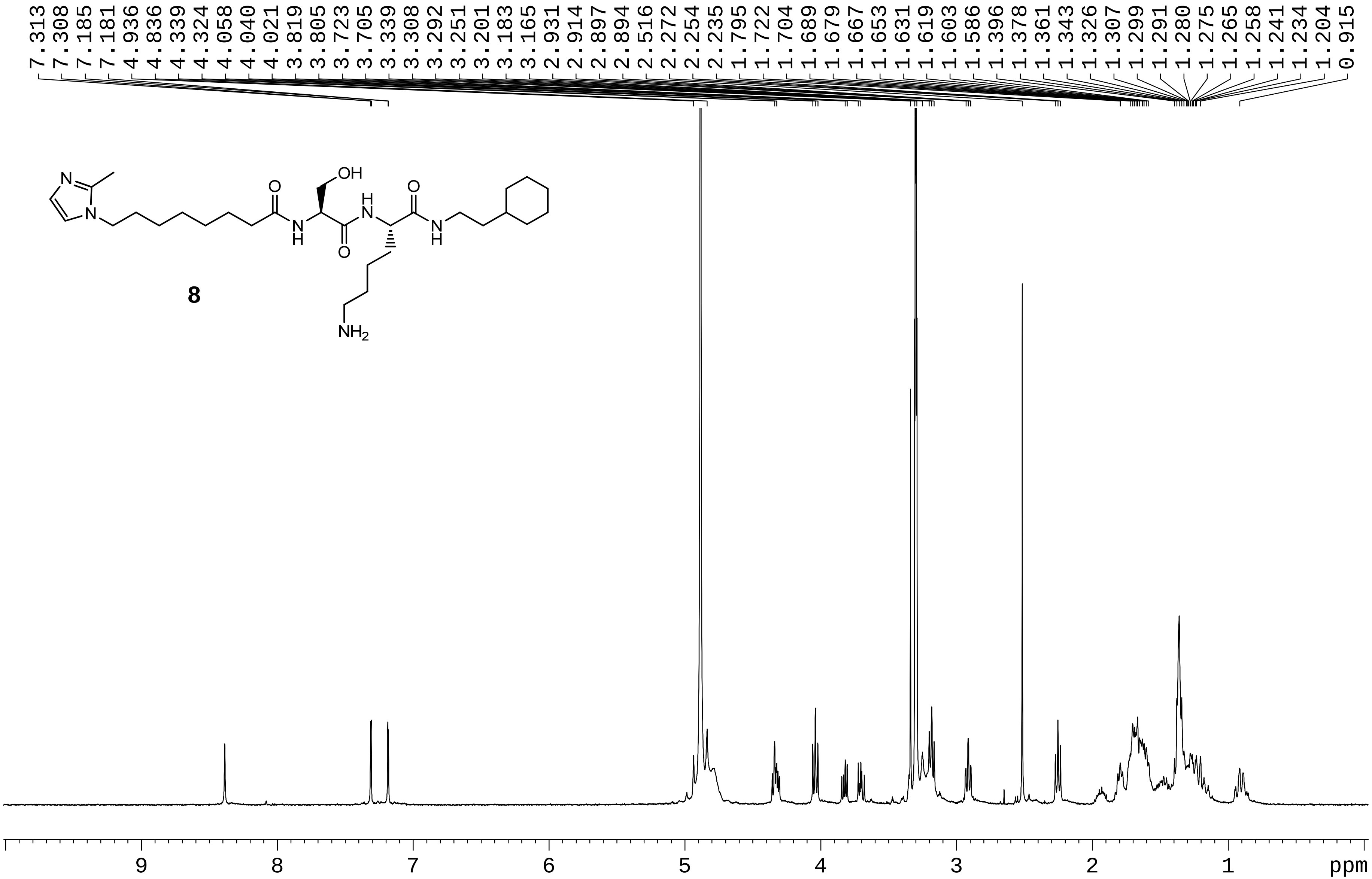
7.313 7.308 7.185 7.181 4.936 4.836 4.339 4.324 4.058 4.040 4.021 3.819 3.805 3.723 3.705 3.339 3.308 3.292 3.251 3.201 3.183 3.165 2.931 2.914 2.897 2.894 2.516 2.272 2.254 2.235 1.795 1.722 1.704 1.689 1.679 1.667 1.653 1.631 1.619 1.603 1.586 1.396 1.378 1.361 1.343 1.326 1.307 1.299 1.291 1.280 1.275 1.265 1.258 1.241 1.234 1.204 0.915
N
N
OH
O
H
N
O
N
H
N
H
O
NH2
8
9
8
7
6
5
4
3
2
1
ppm

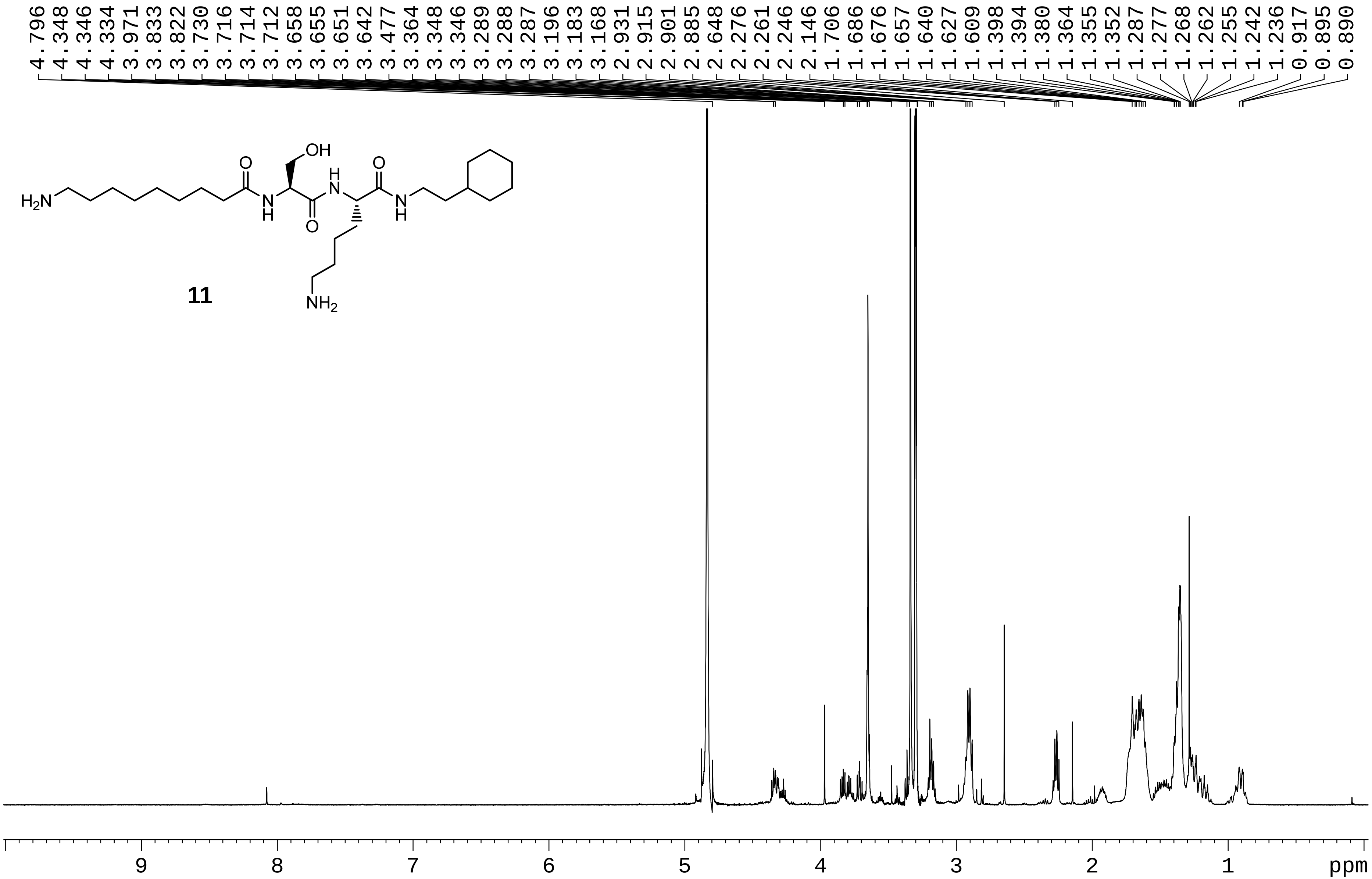
4.796 4.348 4.346 4.334 3.971 3.833 3.822 3.730 3.716 3.714 3.712 3.658 3.655 3.651 3.642 3.477 3.364 3.348 3.346 3.289 3.288 3.287 3.196 3.183 3.168 2.931 2.915 2.901 2.885 2.648 2.276 2.261 2.246 2.146 1.706 1.686 1.676 1.657 1.640 1.627 1.609 1.398 1.394 1.380 1.364 1.355 1.352 1.287 1.277 1.268 1.262 1.255 1.242 1.236 0.917 0.895 0.890
H2N
OH
O
N
H
O
NH2
11
9 8 7 6 5 4 3 2 1 ppm

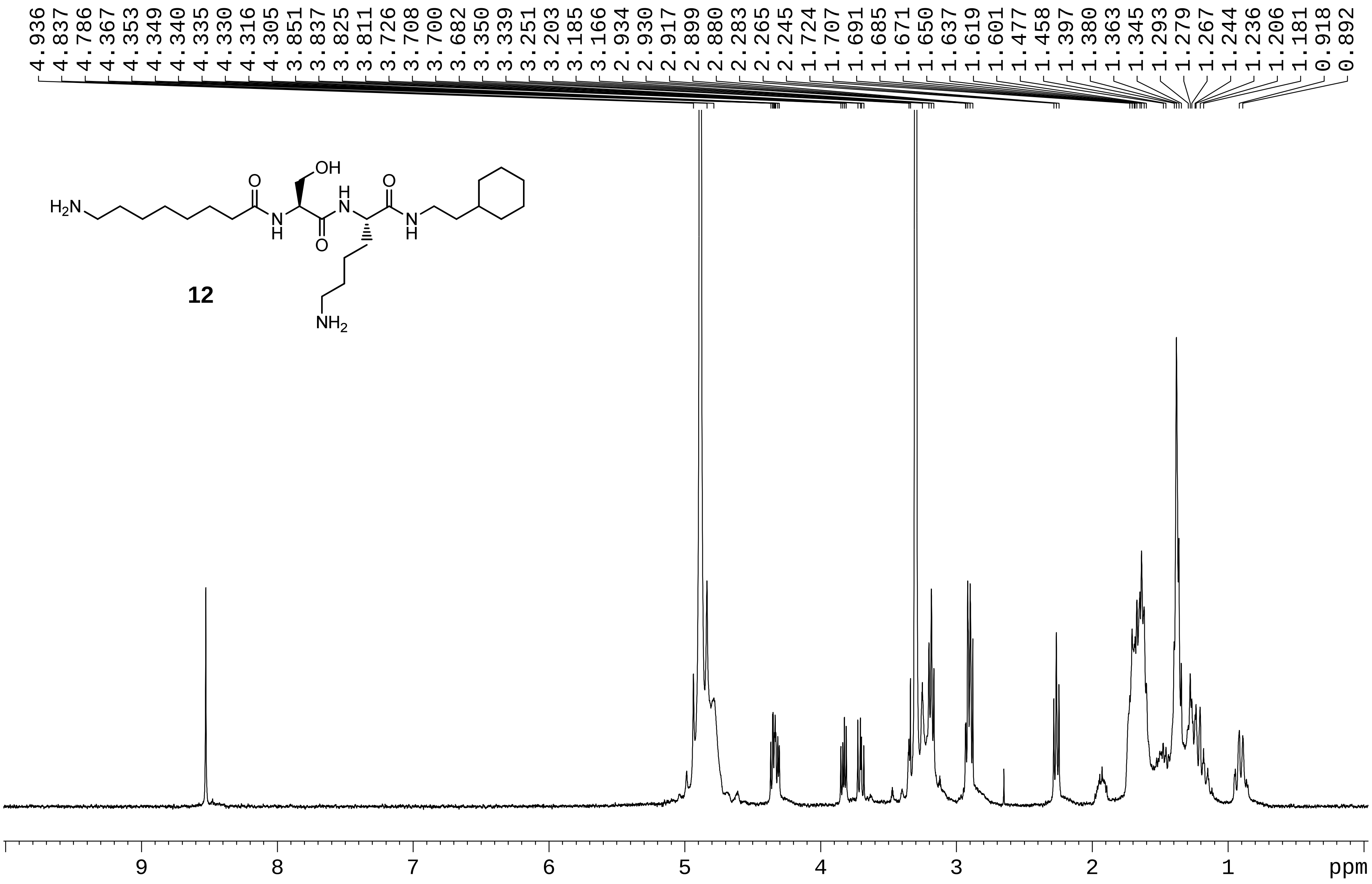
4.936 4.837 4.786 4.367 4.353 4.349 4.340 4.335 4.330 4.316 4.305 3.851 3.837 3.825 3.811 3.726 3.708 3.700 3.682 3.350 3.339 3.251 3.203 3.185 3.166 2.934 2.930 2.917 2.899 2.880 2.283 2.265 2.245 1.724 1.707 1.691 1.685 1.671 1.650 1.637 1.619 1.601 1.477 1.458 1.397 1.380 1.363 1.345 1.293 1.279 1.267 1.244 1.236 1.206 1.181 0.918 0.892
12
9 8 7 6 5 4 3 2 1 ppm

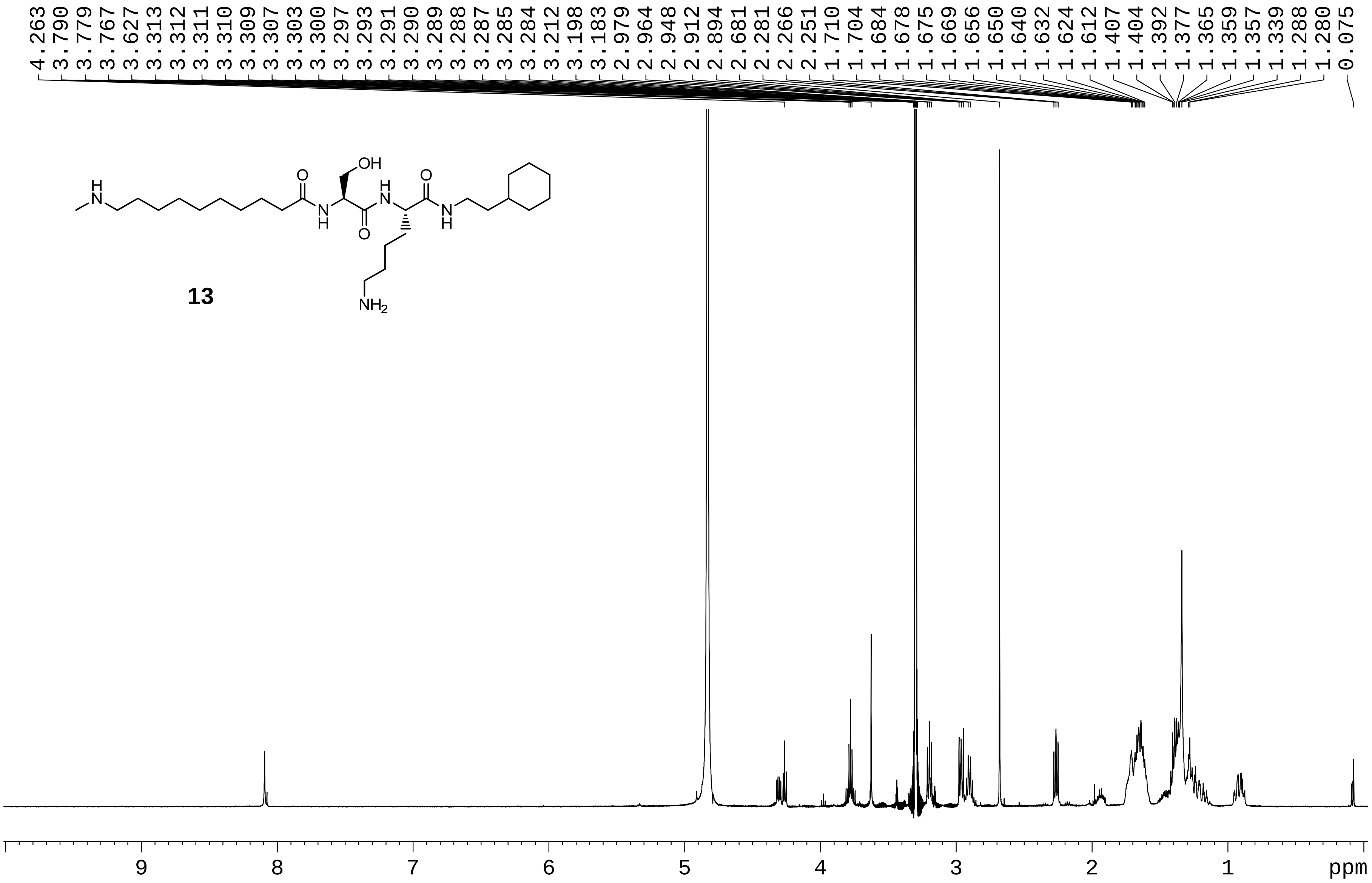
4.263 3.790 3.779 3.767 3.627 3.313 3.312 3.311 3.310 3.309 3.307 3.303 3.300 3.297 3.293 3.291 3.290 3.289 3.288 3.287 3.285 3.284 3.212 3.198 3.183 2.979 2.964 2.948 2.912 2.894 2.681 2.281 2.266 2.251 1.710 1.704 1.684 1.678 1.675 1.669 1.656 1.650 1.640 1.632 1.624 1.612 1.407 1.404 1.392 1.377 1.365 1.359 1.357 1.339 1.288 1.280 0.075
OH
H
N
O
NH2
13
9 8 7 6 5 4 3 2 1 ppm

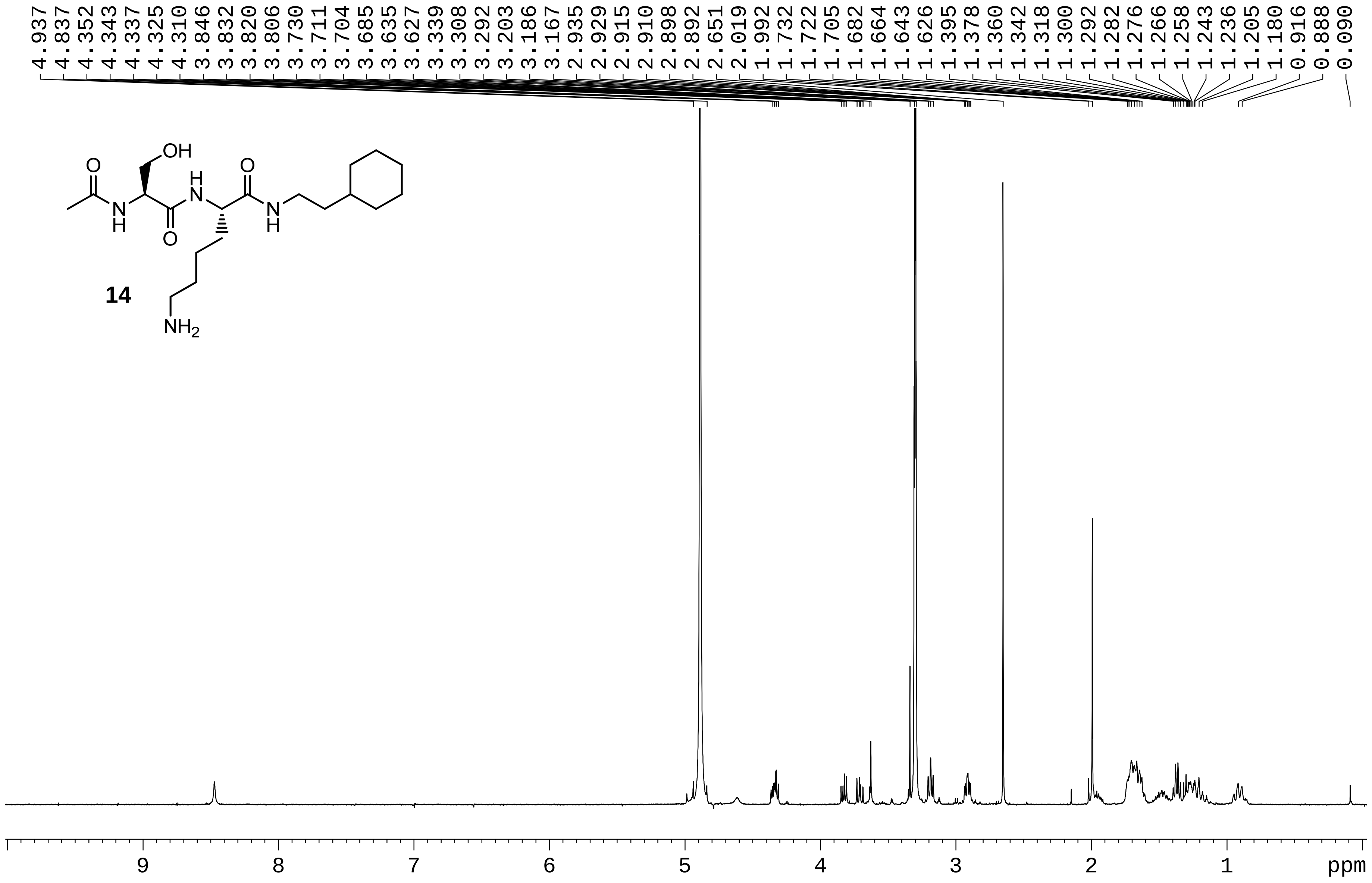
4.937
4.837
4.352
4.343
4.337
4.325
4.310
3.846
3.832
3.820
3.806
3.730
3.711
3.704
3.685
3.635
3.627
3.339
3.308
3.292
3.203
3.186
3.167
2.935
2.929
2.915
2.910
2.898
2.892
2.651
2.019
1.992
1.732
1.722
1.705
1.682
1.664
1.643
1.626
1.395
1.378
1.360
1.342
1.318
1.300
1.292
1.282
1.276
1.266
1.258
1.243
1.236
1.205
1.180
0.916
0.888
0.090
OH
O
N
H
H
N
O
N
H
O
NH2
14
9
8
7
6
5
4
3
2
1
ppm

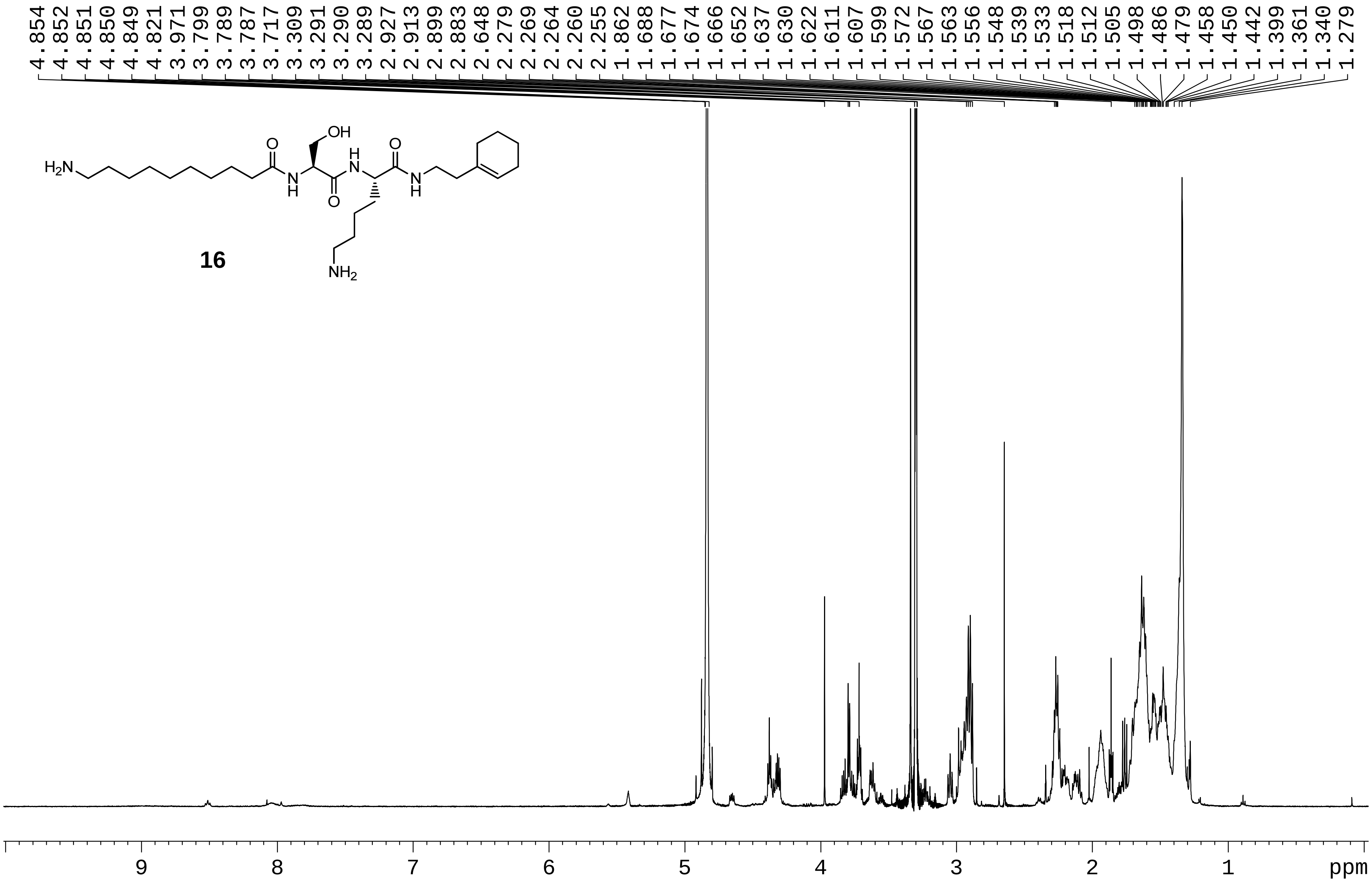
4.854 4.852 4.851 4.850 4.849 4.821 3.971 3.799 3.789 3.787 3.717 3.309 3.291 3.290 3.289 2.927 2.913 2.899 2.883 2.648 2.279 2.269 2.264 2.260 2.255 1.862 1.688 1.677 1.674 1.666 1.652 1.637 1.630 1.622 1.611 1.607 1.599 1.572 1.567 1.563 1.556 1.548 1.539 1.533 1.518 1.512 1.505 1.498 1.486 1.479 1.458 1.450 1.442 1.399 1.361 1.340 1.279
H2N
OH
NH2
16
9
8
7
6
5
4
3
2
1
ppm

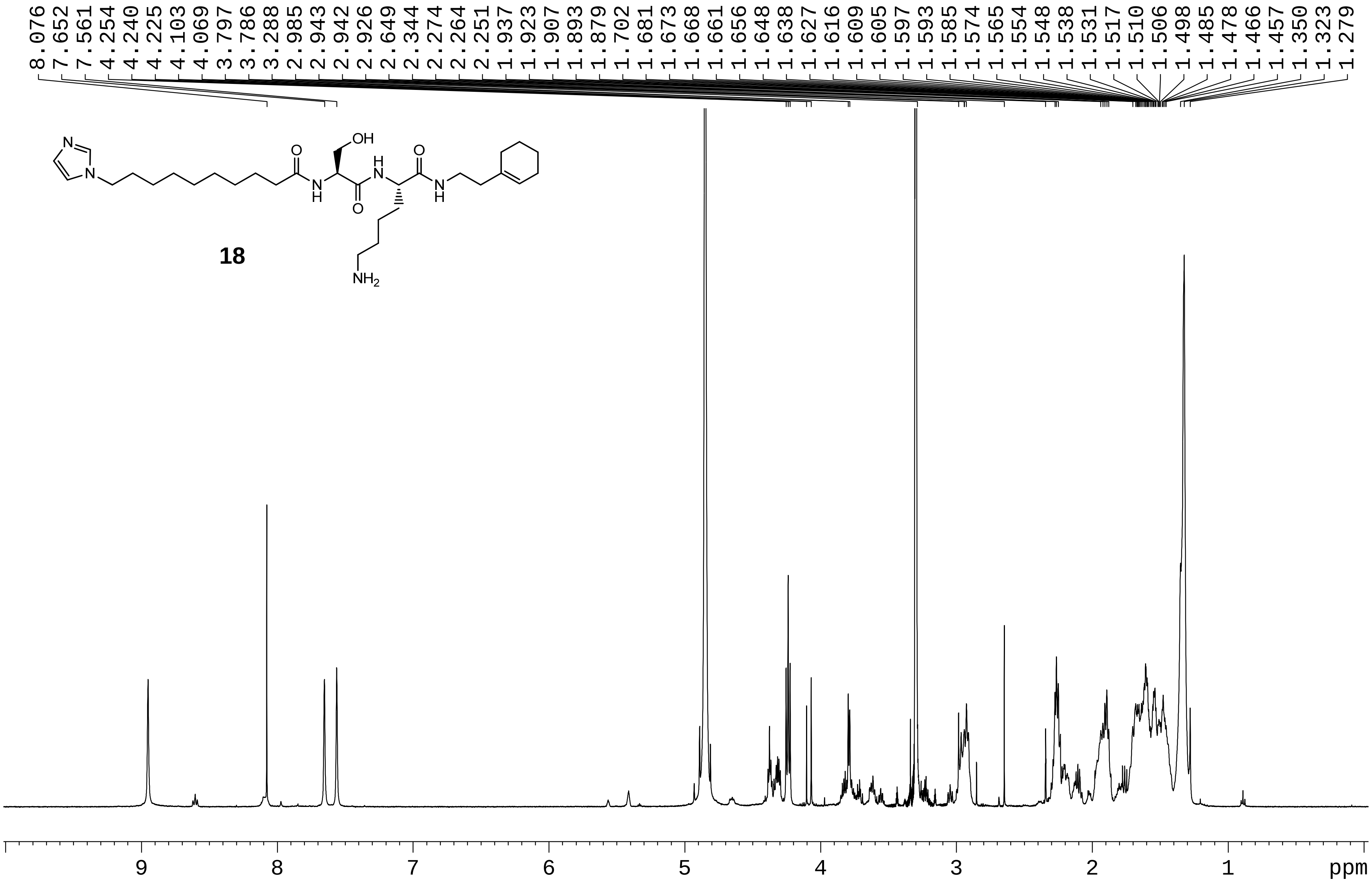
8.076 7.652 7.561 4.254 4.240 4.225 4.103 4.069 3.797 3.786 3.288 2.985 2.943 2.942 2.926 2.649 2.344 2.274 2.264 2.251 1.937 1.923 1.907 1.893 1.879 1.702 1.681 1.673 1.668 1.661 1.656 1.648 1.638 1.627 1.616 1.609 1.605 1.597 1.593 1.585 1.574 1.565 1.554 1.548 1.538 1.531 1.517 1.510 1.506 1.498 1.485 1.478 1.466 1.457 1.350 1.323 1.279
18
9
8
7
6
5
4
3
2
1
ppm

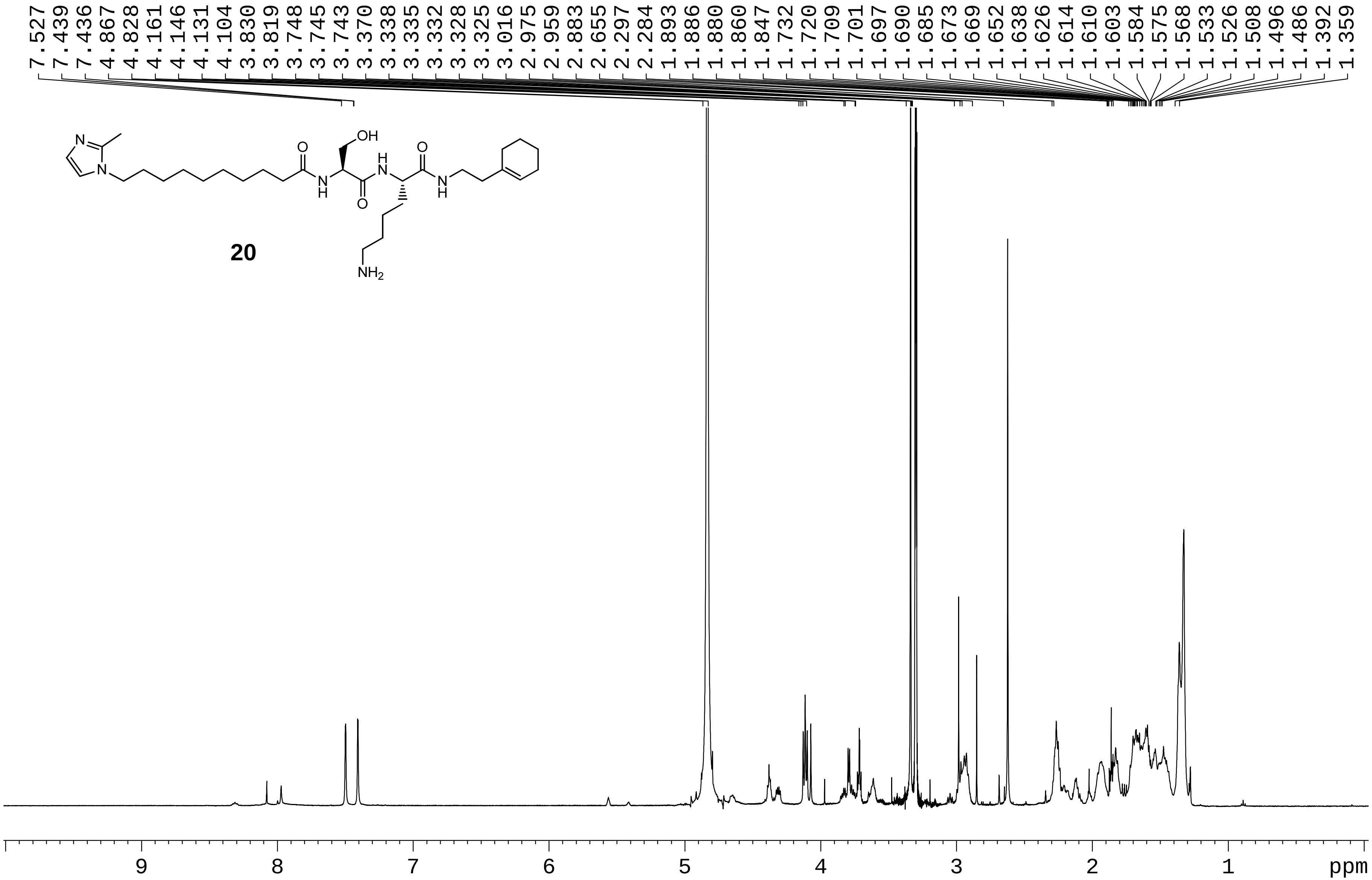
7.527 7.439 7.436 4.867 4.828 4.161 4.146 4.131 4.104 3.830 3.819 3.748 3.745 3.743 3.370 3.338 3.335 3.332 3.328 3.325 3.016 2.975 2.959 2.883 2.655 2.297 2.284 1.893 1.886 1.880 1.860 1.847 1.732 1.720 1.709 1.701 1.697 1.690 1.685 1.673 1.669 1.652 1.638 1.626 1.614 1.610 1.603 1.584 1.575 1.568 1.533 1.526 1.508 1.496 1.486 1.392 1.359
N
N
O
OH
H
N
H
O
O
N
H
NH2
20
9
8
7
6
5
4
3
2
1
ppm

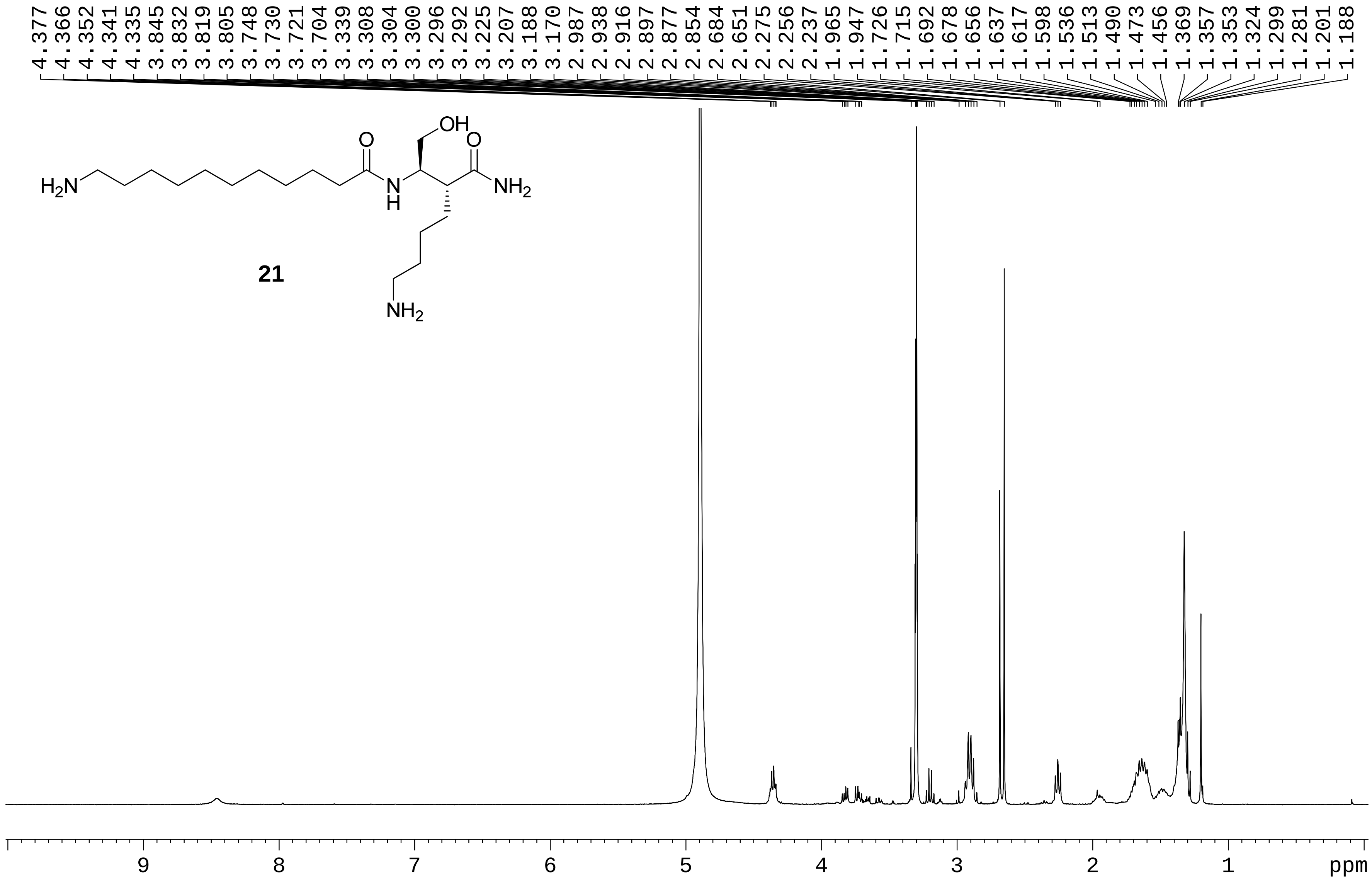
4.377
4.366
4.352
4.341
4.335
3.845
3.832
3.819
3.805
3.748
3.730
3.721
3.704
3.339
3.308
3.304
3.300
3.296
3.292
3.225
3.207
3.188
3.170
2.987
2.938
2.916
2.897
2.877
2.854
2.684
2.651
2.275
2.256
2.237
1.965
1.947
1.726
1.715
1.692
1.678
1.656
1.637
1.617
1.598
1.536
1.513
1.490
1.473
1.456
1.369
1.357
1.353
1.324
1.299
1.281
1.201
1.188
OH
O
O
H_2N
N
H
NH_2
21
NH_2
9
8
7
6
5
4
3
2
1
ppm

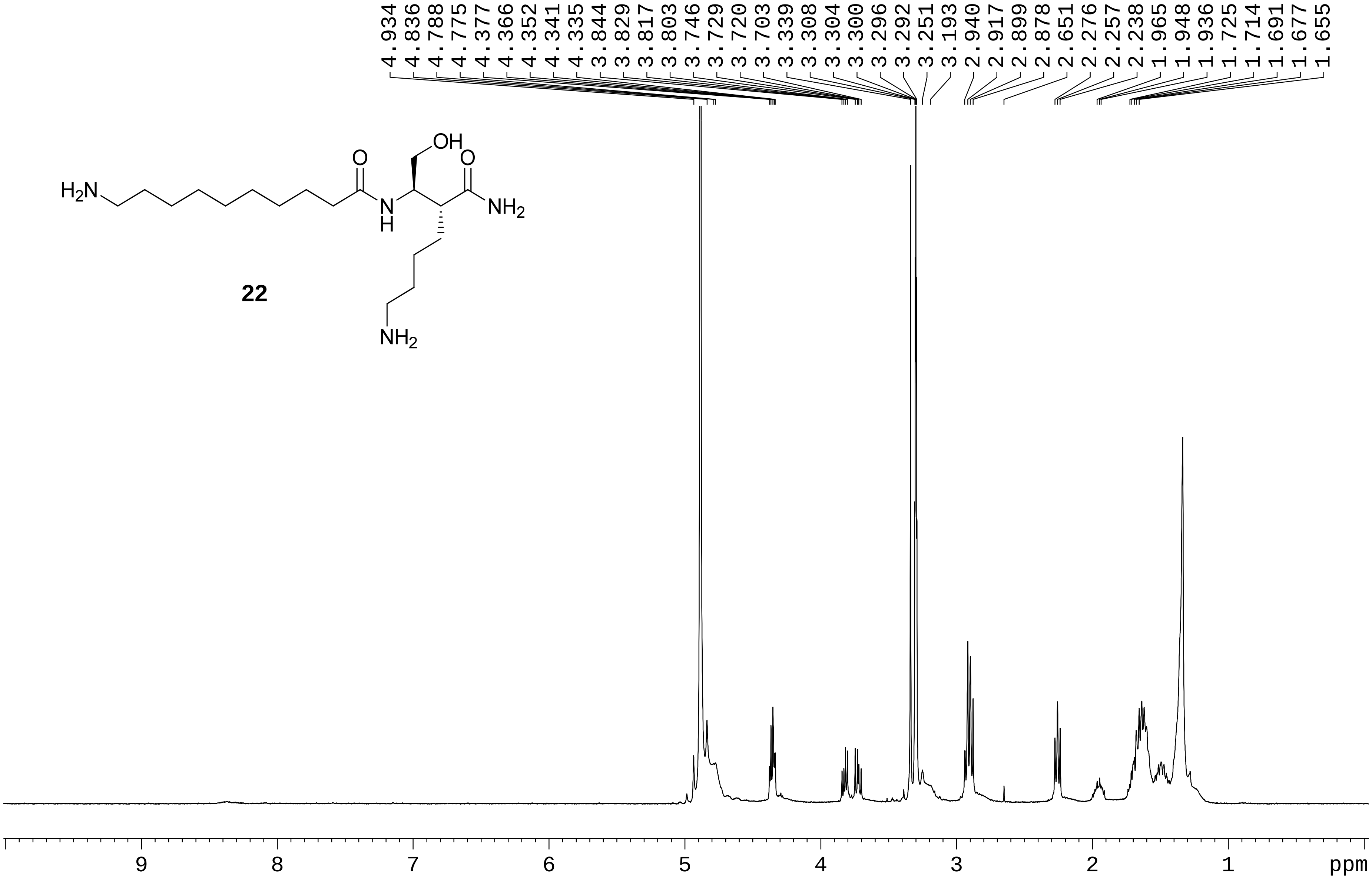
OH
O
O
H2N
N
H
NH2
NH2
4.934
4.836
4.788
4.775
4.377
4.366
4.352
4.341
4.335
3.844
3.829
3.817
3.803
3.746
3.729
3.720
3.703
3.339
3.308
3.304
3.300
3.296
3.292
3.251
3.193
2.940
2.917
2.899
2.878
2.651
2.276
2.257
2.238
1.965
1.948
1.936
1.725
1.714
1.691
1.677
1.655
9
8
7
6
5
4
3
2
1
ppm

22

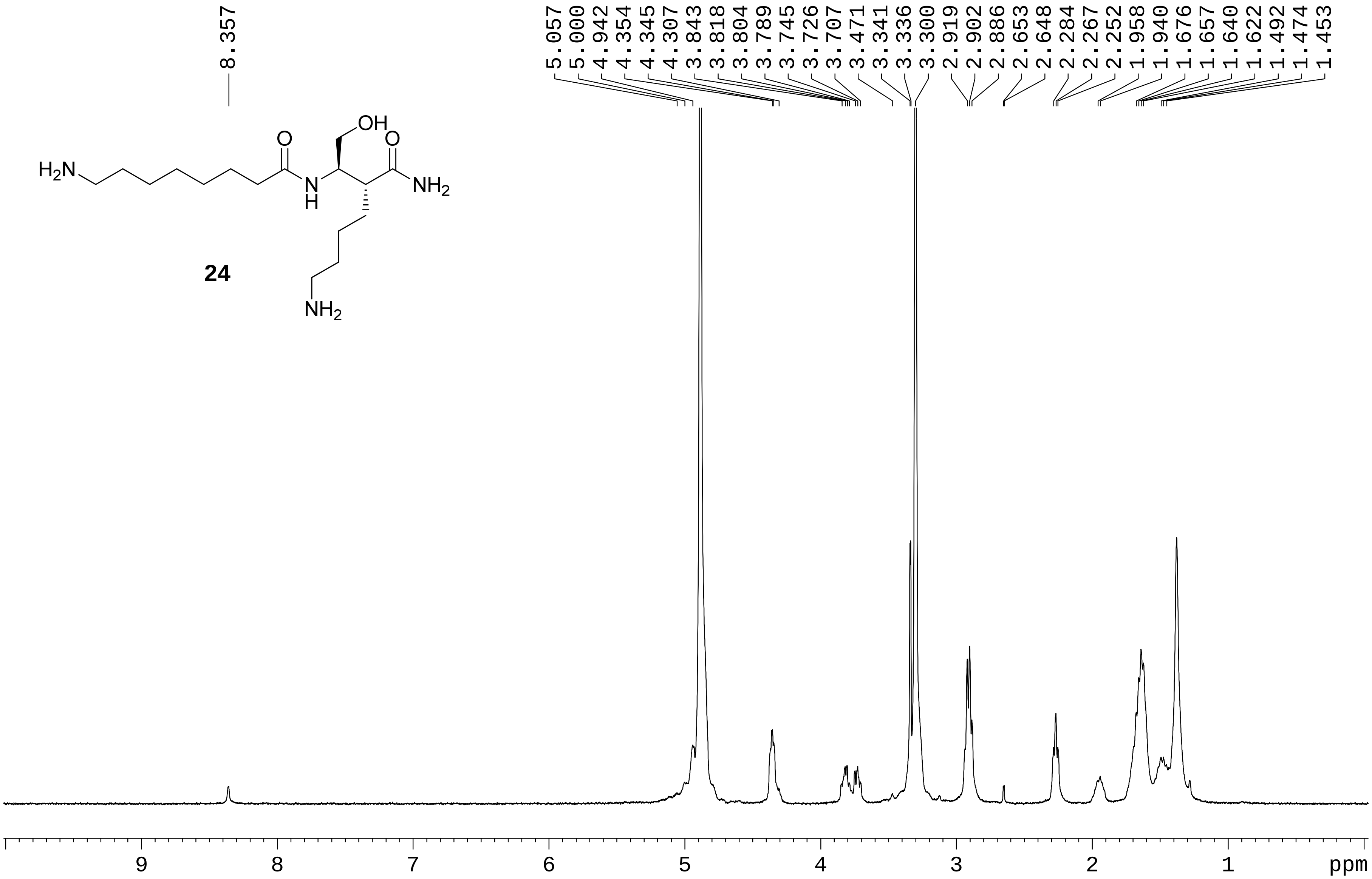
8.357
5.057
5.000
4.942
4.354
4.345
4.307
3.843
3.818
3.804
3.789
3.745
3.726
3.707
3.471
3.341
3.336
3.300
2.919
2.902
2.886
2.653
2.648
2.284
2.267
2.252
1.958
1.940
1.676
1.657
1.640
1.622
1.492
1.474
1.453
OH
O
O
H2N
N
H
NH2
NH2
24
9
8
7
6
5
4
3
2
1
ppm

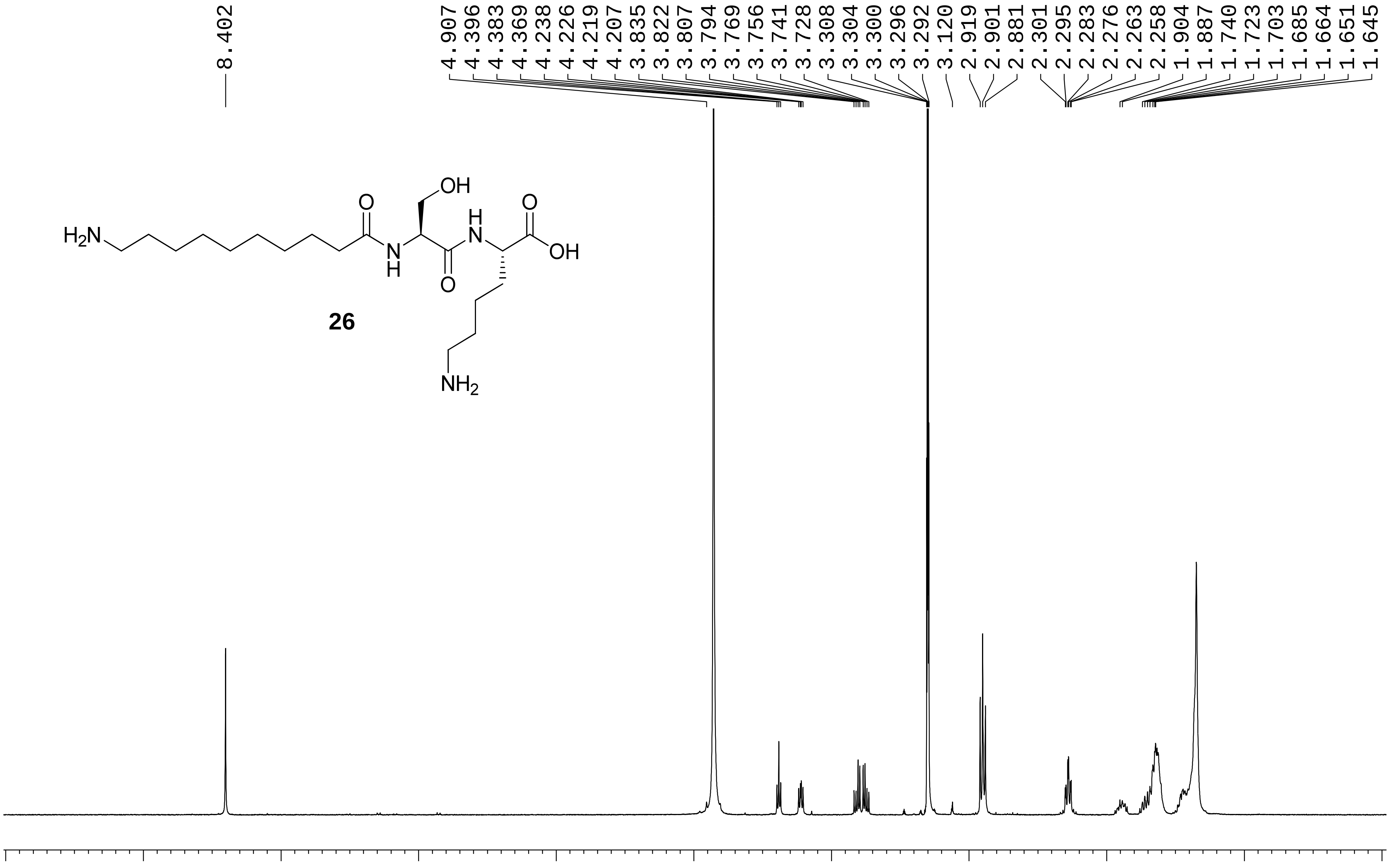
8.402
4.907
4.396
4.383
4.369
4.238
4.226
4.219
4.207
3.835
3.822
3.807
3.794
3.769
3.756
3.741
3.728
3.308
3.304
3.300
3.296
3.292
3.120
2.919
2.901
2.881
2.301
2.295
2.283
2.276
2.263
2.258
1.904
1.887
1.740
1.723
1.703
1.685
1.664
1.651
1.645
26
9 8 7 6 5 4 3 2 1 ppm

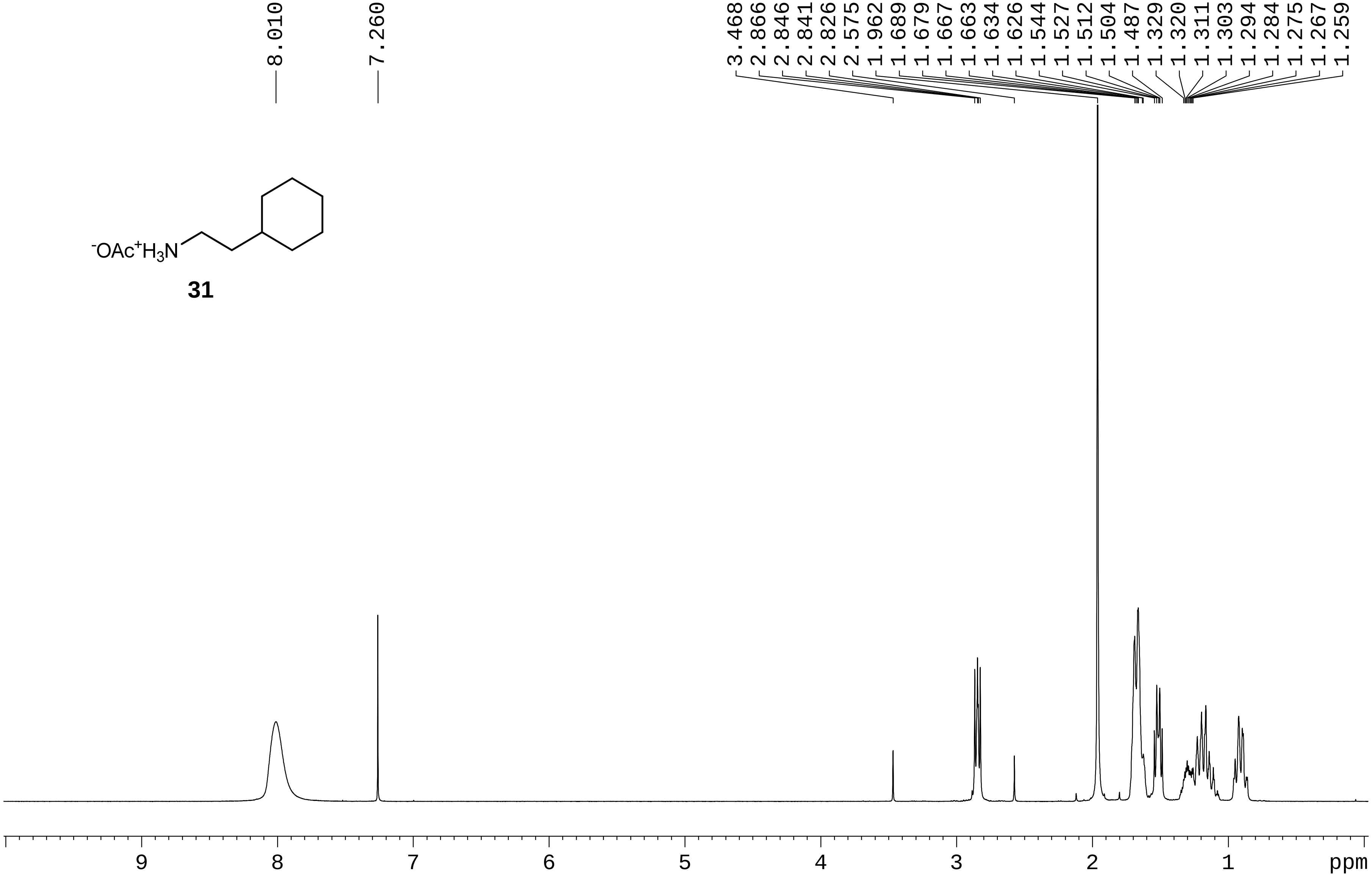
8.010
7.260
3.468
2.866
2.846
2.841
2.826
2.575
1.962
1.689
1.679
1.667
1.663
1.634
1.626
1.544
1.527
1.512
1.504
1.487
1.329
1.320
1.311
1.303
1.294
1.284
1.275
1.267
1.259
-OAc+H3N
31
9
8
7
6
5
4
3
2
1
ppm

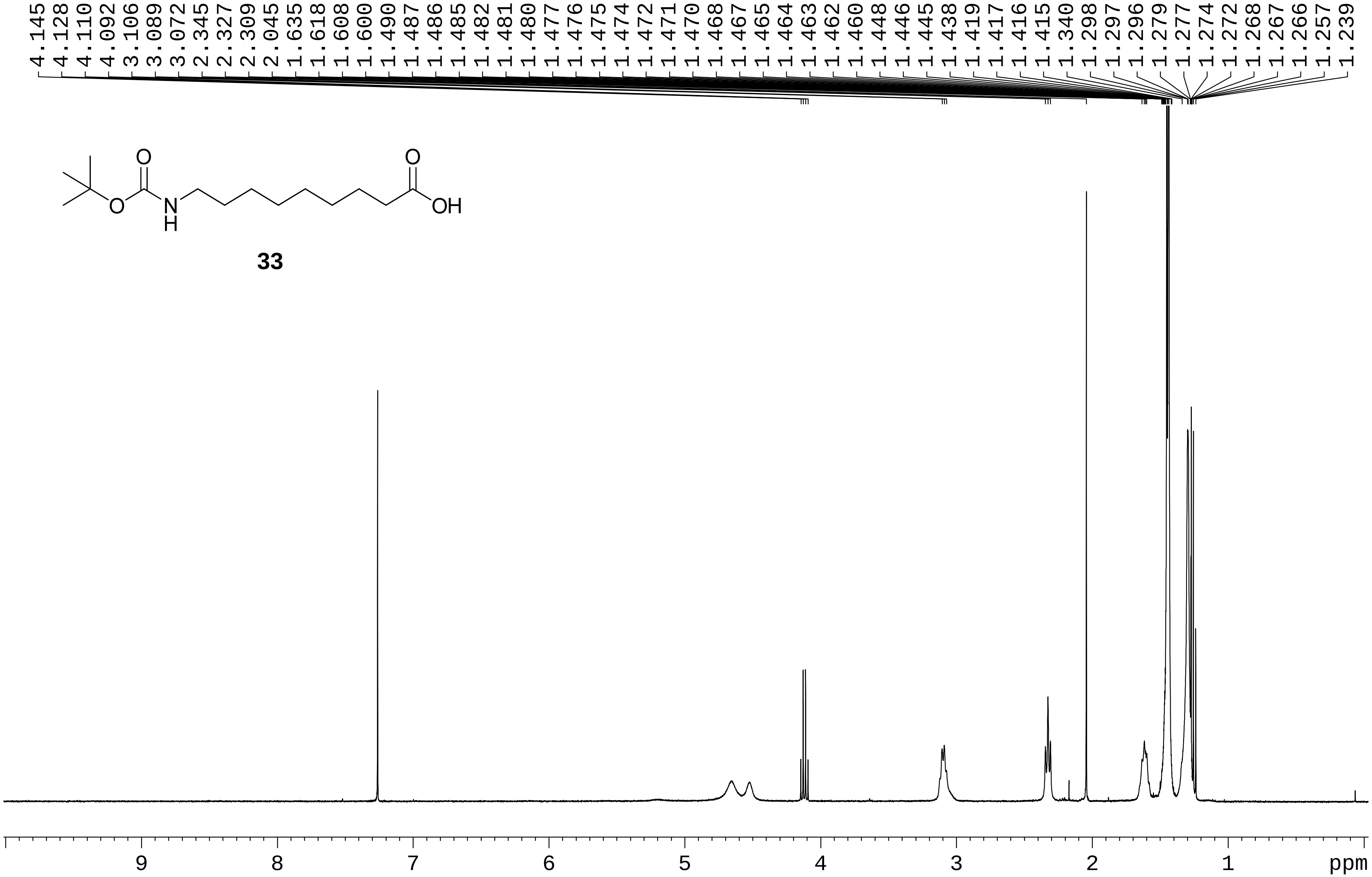

4.145
4.128
4.110
4.092
3.106
3.089
3.072
2.345
2.327
2.309
2.045
1.635
1.618
1.608
1.600
1.490
1.487
1.486
1.485
1.482
1.481
1.480
1.477
1.476
1.475
1.474
1.472
1.471
1.470
1.468
1.467
1.465
1.464
1.463
1.462
1.460
1.448
1.446
1.445
1.438
1.419
1.417
1.416
1.415
1.340
1.298
1.297
1.296
1.279
1.277
1.274
1.272
1.268
1.267
1.266
1.257
1.239
O
N
H
O
O
OH
33
9
8
7
6
5
4
3
2
1
ppm

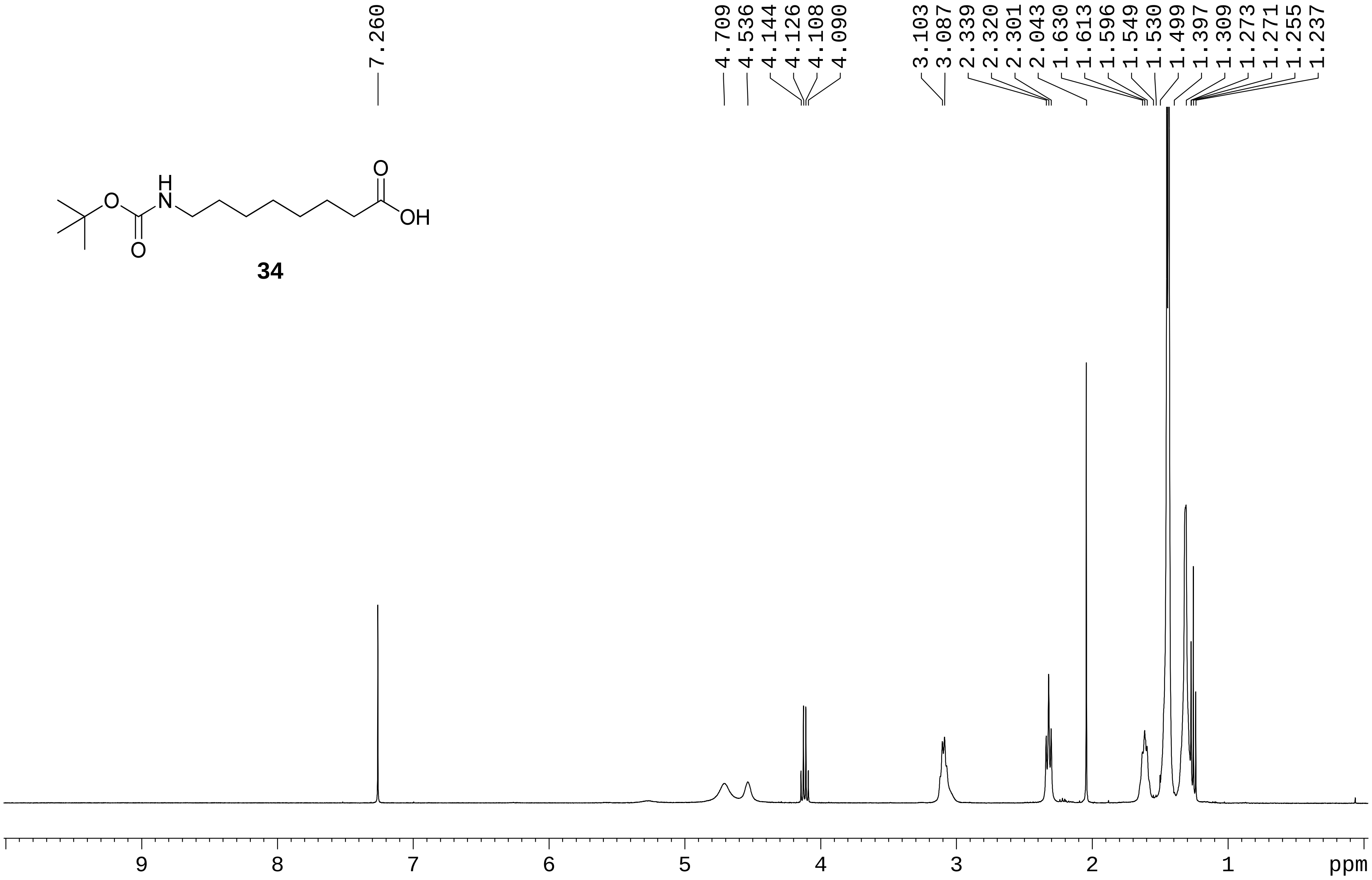

H
N
O
O
O
OH
34
7.260
4.709
4.536
4.144
4.126
4.108
4.090
3.103
3.087
2.339
2.320
2.301
2.043
1.630
1.613
1.596
1.549
1.530
1.499
1.397
1.309
1.273
1.271
1.255
1.237
9
8
7
6
5
4
3
2
1
ppm

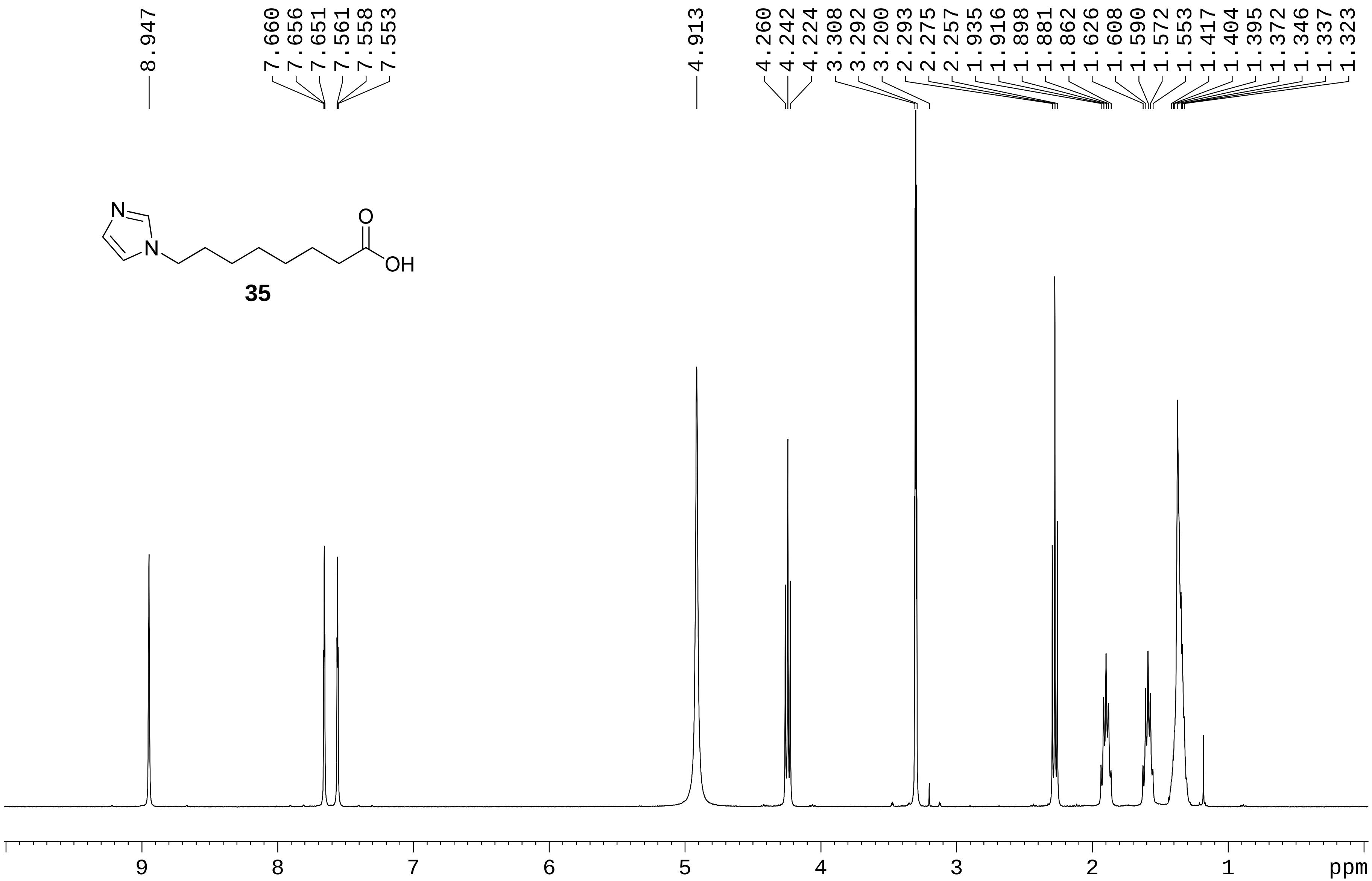
8.947
7.660
7.656
7.651
7.561
7.558
7.553
4.913
4.260
4.242
4.224
3.308
3.292
3.200
2.293
2.275
2.257
1.935
1.916
1.898
1.881
1.862
1.626
1.608
1.590
1.572
1.553
1.417
1.404
1.395
1.372
1.346
1.337
1.323
N
N
O
OH
35
9
8
7
6
5
4
3
2
1
ppm

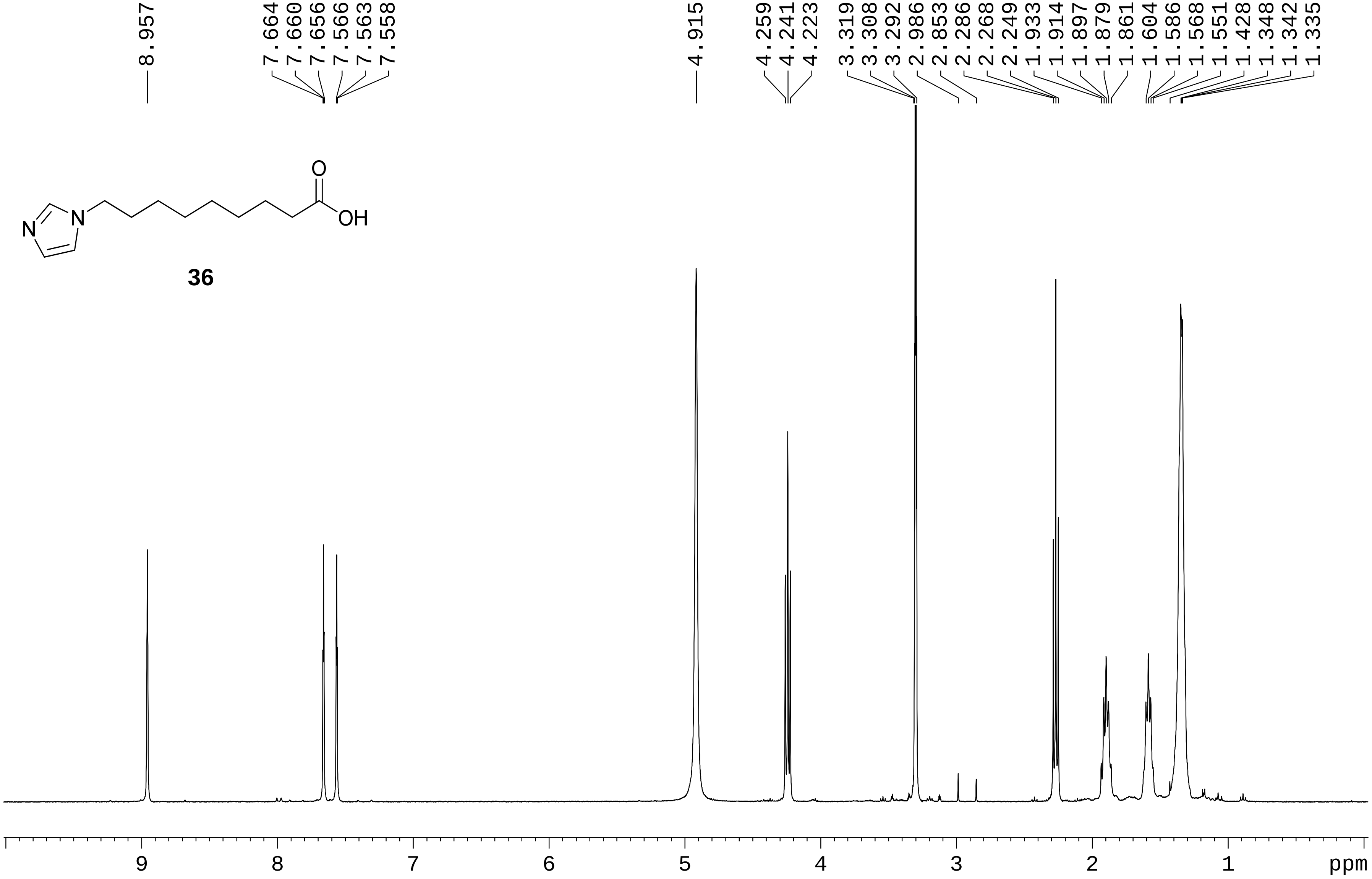
8.957
7.664
7.660
7.656
7.566
7.563
7.558
4.915
4.259
4.241
4.223
3.319
3.308
3.292
2.986
2.853
2.286
2.268
2.249
1.933
1.914
1.897
1.879
1.861
1.604
1.586
1.568
1.551
1.428
1.348
1.342
1.335
O
N
N
OH
36
9
8
7
6
5
4
3
2
1
ppm

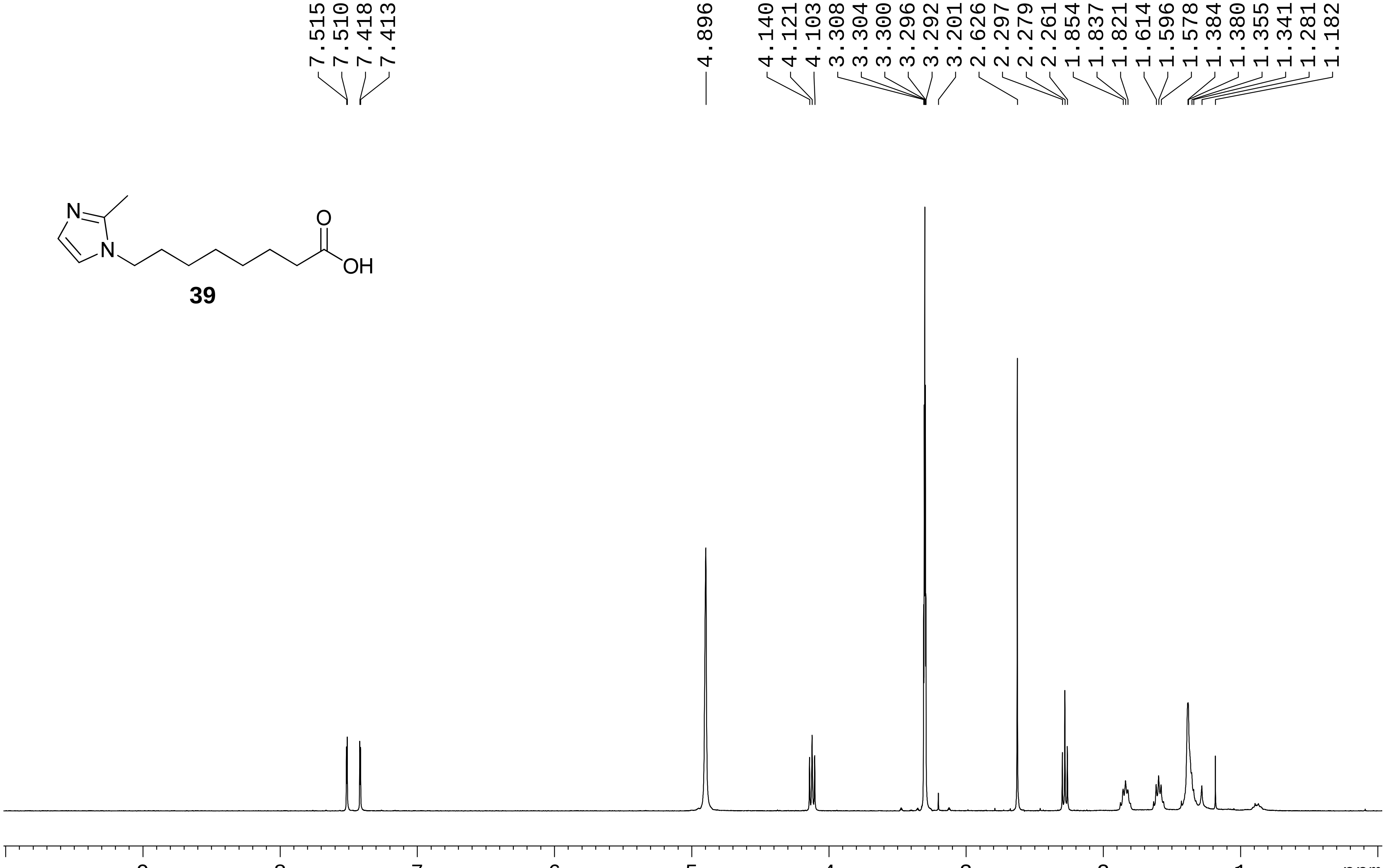
7.515
7.510
7.418
7.413
4.896
4.140
4.121
4.103
3.308
3.304
3.300
3.296
3.292
3.201
2.626
2.297
2.279
2.261
1.854
1.837
1.821
1.614
1.596
1.578
1.384
1.380
1.355
1.341
1.281
1.182
N
N
O
OH
39
9
8
7
6
5
4
3
2
1
ppm

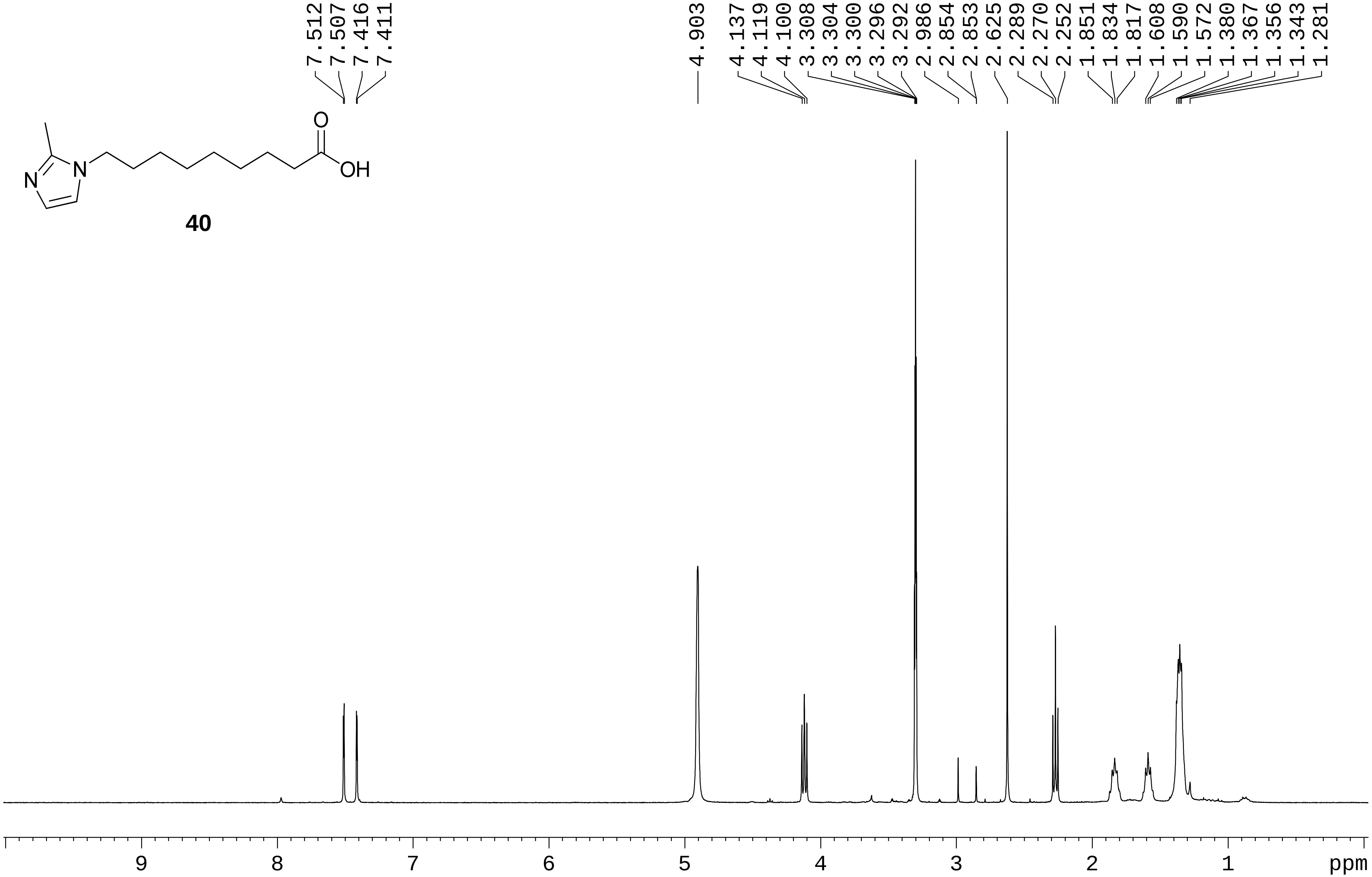

N
N
O
OH
7.512
7.507
7.416
7.411
4.903
4.137
4.119
4.100
3.308
3.304
3.300
3.296
3.292
2.986
2.854
2.853
2.625
2.289
2.270
2.252
1.851
1.834
1.817
1.608
1.590
1.572
1.380
1.367
1.356
1.343
1.281
9
8
7
6
5
4
3
2
1
ppm

40

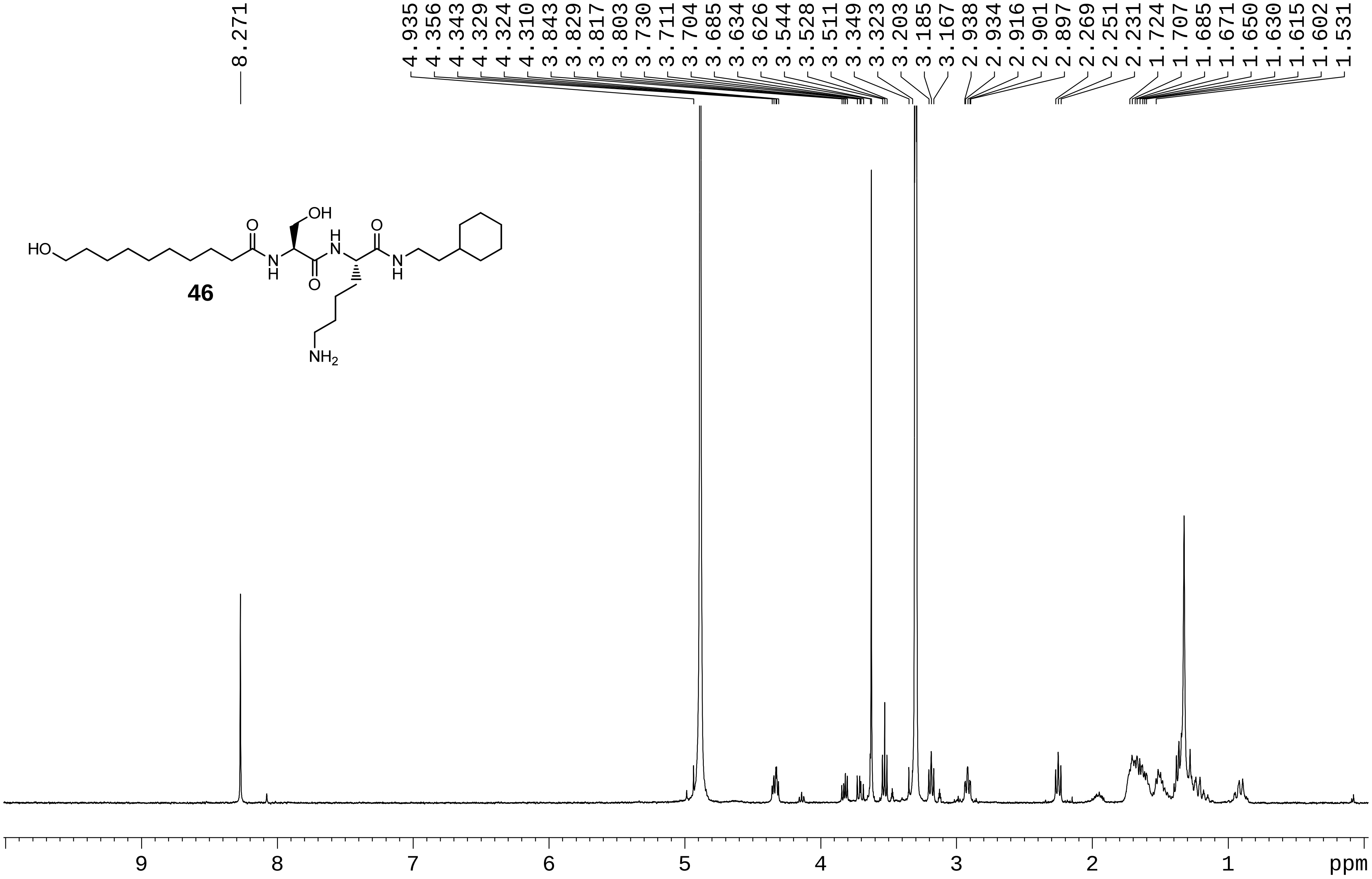
8.271
4.935
4.356
4.343
4.329
4.324
4.310
3.843
3.829
3.817
3.803
3.730
3.711
3.704
3.685
3.634
3.626
3.544
3.528
3.511
3.349
3.323
3.203
3.185
3.167
2.938
2.934
2.916
2.901
2.897
2.269
2.251
2.231
1.724
1.707
1.685
1.671
1.650
1.630
1.615
1.602
1.531
HO
O
N
H
OH
H
N
O
O
N
H
NH_2
46
9
8
7
6
5
4
3
2
1
ppm

1.3. Sequence alignments of NMTs from different species.

Alignment also shows residues within 6 Å of the peptide pocket **in blue**, and their equivalents in other NMT enzymes.

```
PvNMT   EDVRKDEYKLPPGYSWYVCDVKDEKDRSEIYTLLTDNYVEDDDNIFRFNYSAEFLLWALT 117
PfNMT   EDVRKEEYKLPSGYAWCVCDITKENDRSDIYNLLTDNYVEDDDNVFRFNYSSEFLLWALS 117
LdNMT   ADIPEEPYPIASTFEWWTPNMEAADDIHAIYELLRDNYVEDDDSMFRFNYSEEFLQWALC 102
HsNMT1  DNIRQEPYTLPQGFTWDALDLGDRGVLKELYTLLNENYVEDDDNMFRFDYSPEFLLWALR 200

PvNMT   SPNYLKTWHIGVKYDASNKLIGFISAIPTDICIHK--RTIKMAEVNFLCVHKTLRSKRLA 175
PfNMT   SPNYVKNWHIGVKYESTNKLVGFISAIPIDMCVNKN--IIKMAEVNFLCVHKSLRSKRLA 175
LdNMT   PPSYIPDWHVAVRRKADKKLLAFIAGVPVTLRMG----------------TPKYMKVKA 145
HsNMT1  PPGWLPQWHCGVRVVSSRKLVGFISAIPANIHIYDTEKKM--VEINFLCVHKKLRSKRVA 258

PvNMT   PVLIKEITRRINLENIWQAIYTAGVYLPKPVSDARYYHRSINVKKLIEIGFSSLNSRLTM 235
PfNMT   PVLIKEITRRINLESIWQAIYTAGVYLPKPISTARYFHRSINVKKLIEIGFSCLNTRLTM 235
LdNMT   PILIKEVTRRVNRTNVWQAVYTAGVLLPTPYASGQYFHRSLNPEKLVEIRFSGIPAQYQK 241
HsNMT1  PVLIREITRRVHLEGIFQAVYTAGVVLPKPVGTCRYWHRSLNPRKLIEVKFSHLSRNMTM 318

PvNMT   SRAIKLYRVEDTLNIKNMRLMKKKDVEGVHKLLGSYLEQFNLYAVFTKEEIAHWFLPIE- 294
PfNMT   SRAIKLYRIDDTLNIKNLRLMKKKDIDGLQKLLNEHLKQYNLHAIFSKEDVAHWFTPIDQ 294
LdNMT   AMLKRNYQLPNAPKNSGLREMKPSDVPQVRRILMNYLDNFDVGPVFSDAEISHYLLPRD- 305
HsNMT1  QRTMKLYRLPETPKTAGLRPMETKDIPVVHQLLTRYLKQFHLTPVMSQEEVEHWFYPQE- 377

PvNMT   ----NVIYTYVNE-ENGKIKDMISFYSLPSQILGNDKYSTLNAAYSFYNVTTT------- 342
PfNMT   -----VIYTYVNE-ENGEIKDLISFYSLPSKVLGNNKYNILNAAFSFYNITTTTTFKN— 347
LdNMT   ----GVVFTYVVENDKK-VTDFFSFYRIPSTVIGNSNYNILNAAYVHYYAATSMP----- 355
HsNMT1  ----NIIDTFVVENANGEVTDFLSFYTLPSTIMNHPTHKSLKAAYSFYNV-------HTQ 426

PvNMT   ATFKQLMQDAILLAKRNNFDVFNALEVMQNKSVFEDLKFGEGDGSLKYYLYNWKCASF-- 400
PfNMT   -----LIQDAICLAKRNNFDVFNALEVMDNYSVFQDLKFGEGDGSLKYYLYNWKCASC-- 400
LdNMT   --LHQLILDLLIVAHSRGFDVCNMVEILDNRSFVEQLKFGAGDGHLRYYFYNWAYPKIKP 413
HsNMT1  TPLLDLMSDALVLAKMKGFDVFNALDLMENKTFLEKLKFGIGDGNLQYYLYNWKCPSMGA 486

PvNMT   ------------APAHVGIVLL 410
PfNMT   ------------HPSKIGIVLL 410
LdNMT   --------------SQVALVML 421
HsNMT1  --------------EKVGLVLQ 496
```

2. X-ray crystallography

2.1. Protein purification and crystallization

Protein expression and purification of LmNMT was essentially as described for LdNMT[8] but using clone LmNMT_SGC:B1 (a kind gift from Ray Hui, Structural Genomics Consortium, Ontario, Canada). This clone encodes an N-terminal 6-histidine tag and cleavage site for TEV protease followed by residues 5-421 of LmNMT. Protein (10 mg/ml) was incubated at 4 °C overnight with 1/20th volume co-factor MyrCoA (10 mM in 50% DMSO) and crystallised by vapour diffusion using a mother liquor typically containing 30% PEG 1500 or 1450, 0.2 M NaCl, 0.1 M Na cacodylate (pH 5.5).[9] Ligand compound **10** (10 mM stock in 50% DMSO) was added to a stabilisation solution (33% PEG 1500, 0.22 M NaCl, 0.11 M Na cacodylate, pH 5.5) to give a final ligand concentration of 1 mM. Ligand solution was used to replace liquid in crystallisation drops containing LmNMT-MyrCoA crystals by careful pipetting, repeated three

times to completely wash away the original drop solution and left to soak for at least 20 hours. Protein expression of PvNMT and crystallisation of the ternary complex with the non-hydrolysable co-factor and compound **10** bound was essentially as described previously.[10]

2.2. Data collection and refinement.

X-ray diffraction data were collected on synchrotron beamlines at the Diamond Light Source and processed using XDS[11] and SCALA[12] implemented within xia2.[13] Data collection and refinement statistics are summarised in Table S1. For R_{free} calculations, 5% of the data were excluded. Cycles of refinement using maximum likelihood methods implemented in REFMAC[14] were interspersed with model building and adjustment using COOT.[15] LmNMT crystals have a single protein molecule in the assymetric unit, with only N-terminal residues preceding Ala11 (numbering as in full-length native LmNMT protein) not visible in the electron density. For PvNMT, complete chains (corresponding to residues 27 - 410, numbering as in full-length protein) can be traced for two of the three molecules in the asymmetric unit. N-terminal residues in all three chains and loop residues 227 - 238 in chain C have not been modeled and these are assumed to be disordered. The final protein structure models display good geometry with only 0.3% (corresponding to the equivalent amino acid residues PvNMT Phe336 and LmNMT His347) lying outside the preferred regions of the Ramachandran plot.

Table S1. X-ray data collection and refinement statistics.

**PDB accession code**	PvNMT-NHM-**10** **4c68**	LmNMT-MyrCoA-**10** **4c7h**	LmNMT-MyrCoA-**46** **4c7i**
Cell dimensions *a*, *b*, *c* (Å)	57.33, 121.43, 178.74	48.66, 91.13, 53.78	48.64, 91.06, 53.60
Cell angles □, □, □ (°)	90.0, 90.0, 90.0	90.0, 113.8, 90.0	90.0, 114.0, 90.0
Space Group	$P2_12_12_1$	$P2_1$	$P2_1$
**Data collection**			
Beamline / Wavelength (Å)	DLS i03 / 0.9763	DLS i041 / 0.9173	DLS i041 / 0.9200
Detector type	ADSC Q315	Pilatus CMOS	Pilatus CMOS
Images x oscillation (°)	900 x 0.2 & 360 x 0.5	1100 x 0.2	900 x 0.2
Resolution (Å)	42–1.38 (1.45–1.38) [a]	23–1.40 (1.43–1.40)	45–1.30 (1.32–1.30)
R_{sym} (%) [b]	10.8 (58.3)	6.4 (63.6)	3.7 (36.6)
$I/\sigma I$	13.4 (2.8)	10.2 (1.9)	14.5 (2.3)
Completeness (%)	99.8 (99.0)	98.7 (98.9)	96.2 (74.0)
Redundancy	8.1 (6.1)	4.2 (4.1)	3.3 (2.3)
**Refinement**			
No. unique reflections	256405	83119	100469
R_{work} / R_{free} [c]	22.0 / 26.0	17.3 / 20.5	18.5 / 23.9
No. atoms	11507	4223	4348
Protein	9882	3476	3650
Ligand	72	36 (51) [e]	36
Co-factor	192	63 (48) [e]	63
Water	1289	540	582
B-factors (Å^2)			
All atoms	13.8	20.2	16.3
Protein	12.0	18.6	14.6
Ligand	17.2	16.1 (16.5) [e]	13.6
Co-factor	9.4	13.5 (32.3) [e]	12.3
Water	21.1	30.6	27.7
R.m.s.deviations [d]			
Bond lengths (Å)	0.025	0.029	0.030
Bond angles (°)	2.490	2.737	2.745

[a] Highest resolution shell is shown in parentheses.
[b] $R_{sym} = \Sigma_{\mathbf{h}}\Sigma_l |I_{\mathbf{h}l} - \langle I_{\mathbf{h}}\rangle| / \Sigma_{\mathbf{h}}\Sigma_l\langle I_{\mathbf{h}}\rangle$, where I_l is the lth observation of reflection h and $\langle I_{\mathbf{h}}\rangle$ is the weighted average intensity for all observations l of reflection h.

[c] $R_{cryst} = \sum||F_o| - |F_c||/\sum|F_o|$ where F_o and F_c are the observed and calculated structure factor amplitudes, respectively. R_{free} is the R_{cryst} calculated with 5% of the reflections omitted from refinement.
[d] Root-mean-square deviation of bond lengths or bond angles from ideal geometry. [e] Values shown in parentheses relate to products of reaction for ligand or co-factor (CoA, see text).

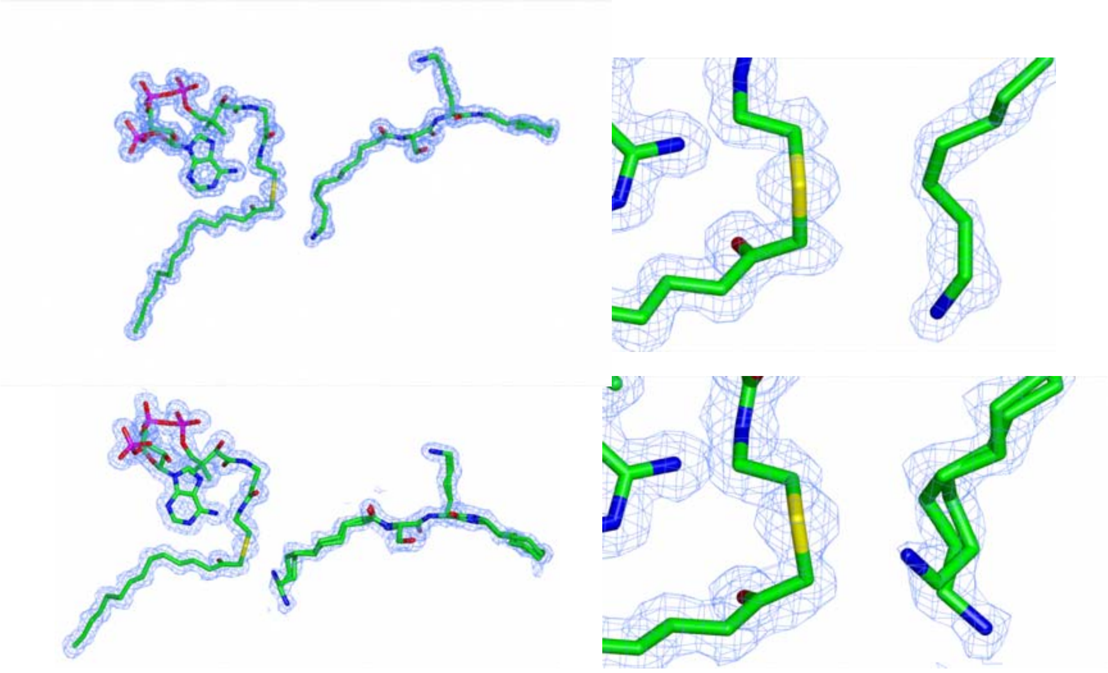

Figure S1: Electron density maps relating to co-factor analogue (NHM) and ligand **10** bound to PvNMT. The ligands are shown in stick representation, coloured by atom; carbon (green), oxygen (red), sulphur (yellow), phosphorus (fuchsia) and nitrogen (blue) with associated refined electron density map (2mF_O-dF_C) contoured at a level of 1 σ. The top panel pair shows ligands bound to protein chain B, with the right-hand side zooming in on a region between the S atom of the co-factor and the N atom at the end of the long ligand alkyl chain. This N-terminus of ligand **10** has been modelled in two slightly different alternate conformations when bound to protein chain A (bottom panel pair). The N-terminal amine (Gly-NH_2) of a substrate peptide would need to flip its carbonyl group to satisfy both equivalent positions. In the *cis* position to the carbonyl, the N-terminal amino group would be able to make a close contact to the carboxylate of Leu410. In the *trans* position the carbonyl could interact with the conserved active site residue Thr197 and the chain would adopt an orientation more similar to that seen in the ternary structure of LmNMT-MyrCoA-**10**. The requirement for conformational flexibility at the N-terminus of a substrate could explain the strict conservation of Gly at this position of the myristoylation sequence motif. There is no evidence from the electron density maps to suggest the presence of ligand **10** in the equivalent binding region of PvNMT protein chain C, which appears to be much more accessible to solvent due to crystal packing.

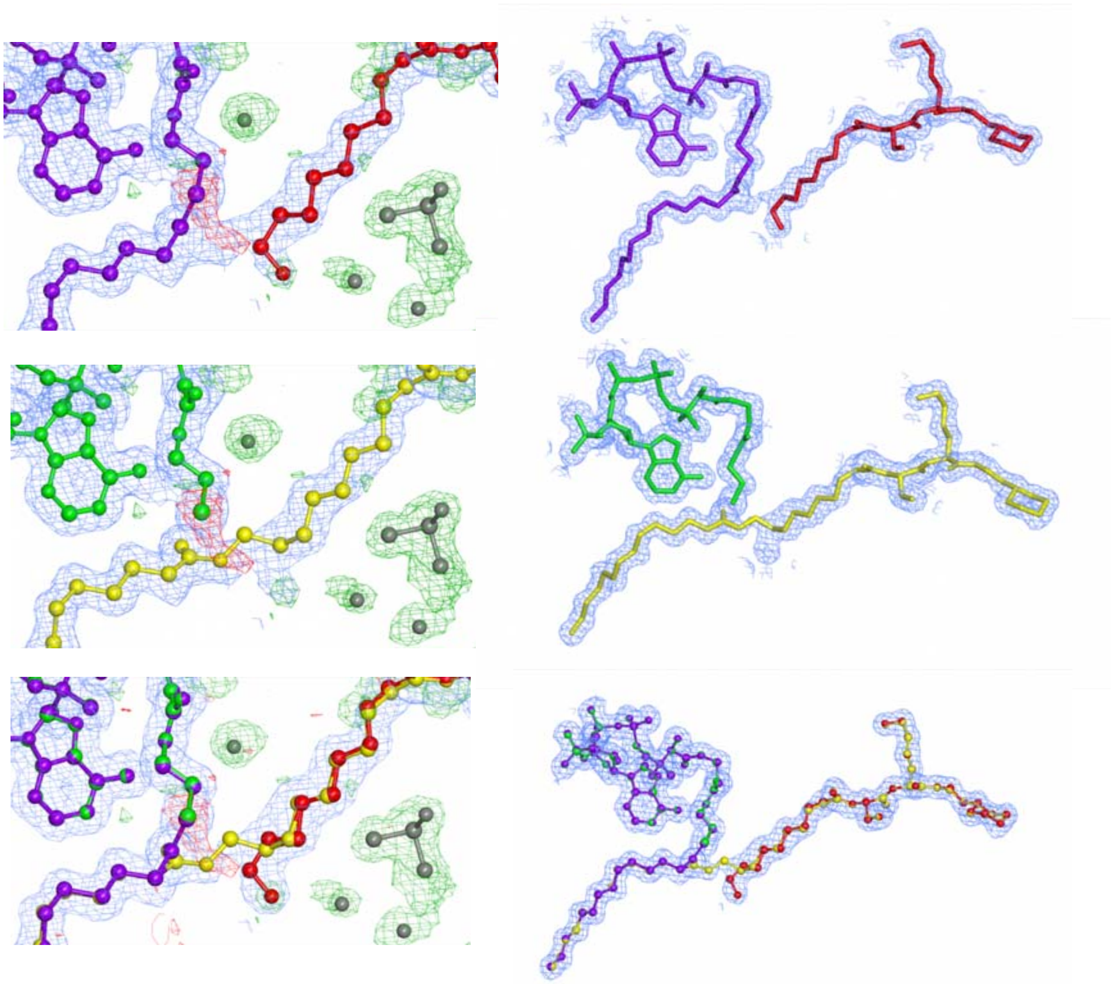

Figure S2: Electron density maps relating to MyrCoA and compound **10** bound to LmNMT. Initial electron density maps calculated at preliminary stages of refinement (left-hand panels) reveal regions of positive (red) and negative (green) density in the difference map (mF_O-dF_C) when MyrCoA and ligand **10** (purple and red ball-and-stick, respectively) were placed in the starting model. Most of the negative density regions were accounted for by the subsequent addition of water or DMSO co-solvent molecules (grey atoms). The electron density remaining was satisfied by modelling the presence of a small proportion (20%) of myristoyl-**10** (yellow) and CoA (green) as putative products of an enzyme-catalysed reaction. The right-hand panels show the final refined electron density map (2mF_O-dF_C) around the ligands contoured at a level of 0.5 σ and with associated individual ligand pairs (MyrCoA and compound **10** reactants or CoA and myristoyl-**10** products) in cylinder representation. The bottom panel shows the mixture of ligands present in the final refined model (80% reactant and 20% product) shown as ball-and-stick to aid identification. Electron density figures were made using the program CCP4mg.[16]

3. Kinetic studies of Compound 10.

As depicted in Figure S3, following kinetic studies, compound **10** showed characteristics similar to that of a competitive inhibitor for PvNMT.

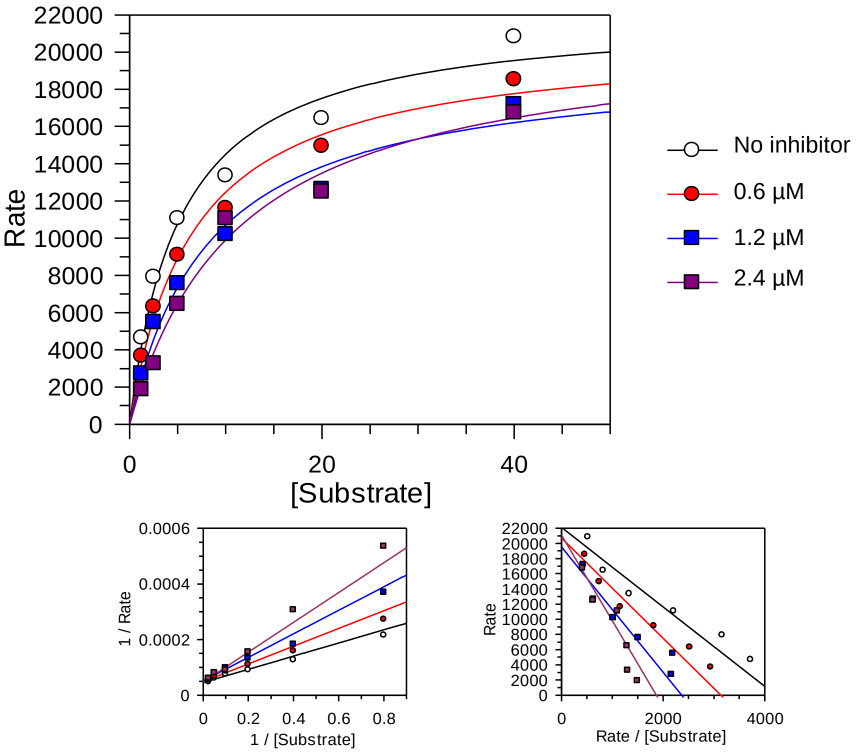

	No inhibitor	0.6 µM	1.2 µM	2.4 µM
V_{max}	22104.5 ± 1365.5	20701.0 ± 1047.4	19563.0 ± 1520.6	21120.3 ± 1651.4
K_M	5.2 ± 1.0	6.6 ± 1.0	8.3 ± 1.8	11.3 ± 2.2

Figure S3: Kinetic assay and Lineweaver-Burk plot studying the competitiveness of compound 10 for PvNMT.

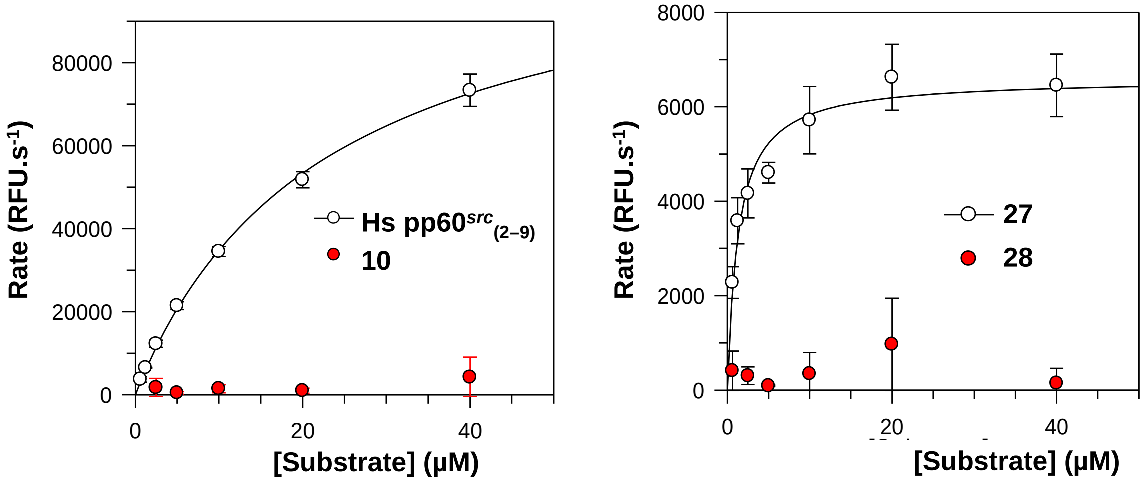

	Hs pp60^{src}$_{(2-9)}$	Compound 10	Compound 27	Compound 28
V_{max}	113616.7 ± 3962.0	Not measurable	6596.4 ± 281.4	Not measurable
K_M	22.6 ± 1.6	Not measurable	1.3 ± 0.3	Not measurable

Figure S4: Substrate characteristics of *H. sapiens* p60^{src}$_{(2\text{-}16)}$, compound 10 and modified inhibitors 27 and 28 in *Leishmania* NMT.

4. References

1. N. Amara, R. Mashiach, D. Amar, P. Krief, S. A. Spieser, M. J. Bottomley, A. Aharoni and M. M. Meijler, *J. Am. Chem. Soc.*, 2009, **131**, 10610-10619.
2. *US Pat.*, 026 415, 2007.
3. *US Pat.*, 6 204 278, 2001.
4. C. M. Jakobsen, S. R. Denmeade, J. T. Isaacs, A. Gady, C. E. Olsen and S. B. Christensen, *J. Med. Chem.*, 2001, **44**, 4696-4703.
5. S. Hack, B. Worlein, G. Hofner, J. Pabel and K. T. Wanner, *Eur. J. Med. Chem.*, 2011, **46**, 1483-1498.
6. *US Pat.*, 0 034 773, 2006.
7. B. Devadas, T. Lu, A. Katoh, N. S. Kishore, A. C. Wade, P. P. Mehta, D. A. Rudnick, M. L. Bryant, S. P. Adams, Q. Li, G. W. Gokel and J. I. Gordon, *J. Biol. Chem.*, 1992, **267**, 7224-7239.
8. J. A. Brannigan, B. A. Smith, Z. Yu, A. M. Brzozowski, M. R. Hodgkinson, A. Maroof, H. P. Price, F. Meier, R. J. Leatherbarrow, E. W. Tate, D. F. Smith and A. J. Wilkinson, *J. Mol. Biol.*, 2010, **396**, 985-999.
9. J. A. Frearson, S. Brand, S. P. McElroy, L. A. T. Cleghorn, O. Smid, L. Stojanovski, H. P. Price, M. L. S. Guther, L. S. Torrie, D. A. Robinson, I. Hallyburton, C. P. Mpamhanga, J. A. Brannigan, A. J. Wilkinson, M. Hodgkinson, R. Hui, W. Qiu, O. G. Raimi, D. M. F. van Aalten, R. Brenk, I. H. Gilbert, K. D. Read, A. H. Fairlamb, M. A. J. Ferguson, D. F. Smith and P. G. Wyatt, *Nature*, 2010, **464**, 728-732.
10. V. Goncalves, J. A. Brannigan, D. Whalley, K. H. Ansell, B. Saxty, A. A. Holder, A. J. Wilkinson, E. W. Tate and R. J. Leatherbarrow, *J. Med. Chem.*, 2012, **55**, 3578-3582.
11. W. Kabsch, *Acta Crystallogr. D Biol. Crystallogr.*, 2010, **66**, 125-132.
12. P. Evans, *Acta Crystallogr. D Biol. Crystallogr.*, 2006, **62**, 72-82.
13. G. Winter, *J. Appl. Crystallogr.*, 2010, **43**, 186-190.
14. G. N. Murshudov, A. A. Vagin and E. J. Dodson, *Acta Crystallogr. D Biol. Crystallogr.*, 1997, **53**, 240-255.
15. P. Emsley, B. Lohkamp, W. G. Scott and K. Cowtan, *Acta Crystallogr. D Biol. Crystallogr.*, 2010, **66**, 486-501.
16. S. McNicholas, E. Potterton, K. S. Wilson and M. E. M. Noble, *Acta Crystallogr. D Biol. Crystallogr.*, 2011, **67**, 386-394.
